# Supplementary material for: Effect of statins on inflammation and cardiac function in patients with chronic Chagas disease: A protocol for pathophysiological studies in a multicenter, placebo-controlled, proof-of-concept phase II trial
Source: PLoS One. 2023 Jan 13;18(1):e0280335. doi: 10.1371/journal.pone.0280335 (PMC9838836; doi:10.1371/journal.pone.0280335)
Supplement: S2 File — (PDF) [file pone.0280335.s002.pdf]

# Clinical Trial Protocol

1. **Project code:** FONDECYT 1210359
2. **Sponsor/Founder**: FONDECYT Grant 1210359
3. **Clinical trial title**

## Effect of statins on inflammation and cardiac function in patients with chronic Chagas disease: pathophysiological studies in a multicenter proof-of-concept clinical trial

4. **Research Team:**

**Principal investigator:**

Dr. Juan Diego Maya Arango. Facultad de Medicina, Universidad de Chile. Institutional address: Avenida Independencia 1027, Independencia, Santiago. Phone: 56 2 2978 6071; email: jdmaya@uchile.cl.

**Co-investigators:**

Marisol del Carmen Denegri Cartes: Departamento de Pediatría Occidente, Facultad de Medicina, Universidad de Chile; Hospital Félix Bulnes, Cerro Navia. email: marisoldenegri@hotmail.com

Miren Edurne Urarte Izeta. Departamento de Pediatría Occidente, Facultad de Medicina, Universidad de Chile; Hospital San Juan De Dios, Santiago. email: eurarte@med.uchile.cl

Litzi Villalón Quezada. Hospital Gustavo Fricke y Hospital San Martin de Quillota; email: litzi.villalon@redsalud.gob.cl

Ulrike Kemmerling Weis: Facultad de Medicina, Universidad de Chile. email: ukemmerling@uchile.cl

Carolina Campos Estrada: Facultad de Farmacia, Universidad de Valparaíso. email: carolina.campos@uv.cl

5. **Study phase:** Phase II proof-of-concept study

## 6. SUMMARY

**Clinical Trial Title**

Effect of statins on inflammation and cardiac function in patients with chronic Chagas disease: pathophysiological studies in a multicenter proof-of-concept clinical trial

**Abbreviated title:** Statins in Chagas disease: a multicenter proof-of-concept clinical trial

**Phase: Phase II proof-of-concept study**

**GOALS:**

**PRIMARY AIM**

- To evaluate whether the use of atorvastatin in combination with antiparasitic therapy (NFX or BZD) is safe and more effective than antiparasitic therapy alone in preventing the occurrence of cardiac disorders by reducing overall inflammation and improving endothelial and cardiac function

**SECONDARY AIMS**

- To evaluate the efficacy of the combination of ATO and antichagasic therapy to decrease:
    - Inflammation, as measured by plasma levels of the cytokines, TNF-α, IFN-γ, IL-10, IL-1B, IL-4, and IL-17A.
    - Endothelial activation, as measured by plasma levels of sCAM: sE-selectin, sICAM-1, and sVCAM-1.
    - Cardiac damage and function: measured by plasma levels of BNP, cTnT, 12-lead resting ECG, and 2D echocardiogram.
- To determine the safety and tolerability of the combination of ATO with antichagasic therapy, measured by the incidence of AEs (e.g., rhabdomyolysis) and treatment discontinuation.
- To evaluate the treatment response to the combination of ATO with antichagasic therapy, measured by quantitative PCR and serology, over a 10-month follow-up period.
- To determine the degree of adherence to therapies by measuring drug accountability throughout the study

**STUDY DESIGN:** This is a proof-of-concept Phase II clinical trial with three different groups: two groups will receive antichagasic therapy plus 40 or 80 mg/day of ATO, respectively. The third group will receive a placebo plus antichagasic therapy. Patients will first receive conventional antichagasic treatment for two months. After a washout of two weeks (or until normalization of liver enzymes), treatment with atorvastatin or placebo will be initiated for two months. These doses were chosen to minimize false negative results, thus providing the best hypothesis test and maximizing the pharmacodynamic effect on inflammation and cardiac and endothelial function. In addition, the treatment sequence aims to minimize adverse reactions that could be caused by the eventual combination of antichagasic drugs with atorvastatin. Study subjects will be randomized, and the three groups will be double-blinded (both clinical investigators and subjects will be blinded) to ATO or placebo. However, antichagasic therapy will be open-label. In addition, qPCR and the other laboratory evaluations, as well as ECG and 2D echocardiograms, will be performed with the clinical investigators blinded to ATO or placebo allocation.

As mentioned above, this study will be conducted at four centers: 1) Hospital San Juan de Dios and 2) Hospital Felix Bulnes in Santiago, Metropolitan Region, 3) Hospital Dr. Gustavo Fricke in Viña del Mar, and

4) Hospital San Martin de Quillota, Valparaiso Region.

**ABSTRACT:** Cardiac complications, including heart failure and arrhythmias, are major causes of disability and death in Chagas disease (CD). We postulate that statins ameliorate vascular and cardiac inflammation in patients with chronic CD, contributing to the recovery of endothelial and cardiac function and thereby improving the efficacy of antichagasic therapy.

CD, caused by the protozoan parasite Trypanosoma cruzi, affects 7 million people in Latin America, and its incidence is increasing in non-endemic countries due to migration. It is the second disease with the highest burden among tropical diseases, and its global economic cost exceeds US$ 7 billion per year. Diagnosis is based on clinical suspicion and serological detection of antibodies. Cardiac evaluation is essential to determine the functional status of the heart and the risk of mortality. Cardiac involvement is explained by parasite-dependent immune-mediated myocardial injury, microvascular abnormalities, and ischemia. Maya et al. demonstrated that a hallmark of T. cruzi-induced microvascular damage is an increase in vascular endothelial adhesion molecules, an effect counteracted by the cholesterol-lowering drug simvastatin.

Current treatment of early CD includes the administration of nifurtimox and benznidazole. In contrast, in the chronic phase, their efficacy is low and may induce severe adverse events, forcing discontinuation of therapy. Therefore, finding innovative approaches to treat this potentially fatal disease is of utmost importance. Thus, improving the efficacy of current antichagasic drugs by modifying the inflammatory response would make current treatment more effective.

In the most recent preclinical studies with chronically infected mice, simvastatin was shown to (i) decrease cardiac inflammation, (ii) reduce endothelial activation, and (iii) improve cardiac function. These effects require clinical confirmation. Thus, our proposal aims to analyze whether a statin, through the abovementioned changes, could improve antichagasic therapy in chronic CD through a phase II clinical trial. Therefore, we propose the coadministration of atorvastatin (ATO) as an experimental statin. Their therapeutic and safety profiles are well known, as well as their mechanism of action, which other members of the statin class share. Due to the low incidence of serious adverse effects and their efficacy, both are currently the most widely used statins.

This proposal aims to evaluate whether treatment with 40 or 80 mg of ATO, in combination with anti-statin therapy, is safe and more effective in reducing overall inflammation than an anti-statin therapy alone, allowing investigators to test the hypothesis that the anti-inflammatory effect of anti-inflammatory statins improves endothelial and cardiac functions.

This proof-of-concept trial will be a double-blind, randomized, multicenter, phase II design.

To achieve this goal, we will evaluate the efficacy of the combination of ATO and antichagasic therapy in reducing plasma levels of inflammatory cytokines and soluble endothelial cell adhesion molecules and confirm improvement in cardiac function by electrocardiogram and two-dimensional echocardiogram.

The study will establish the safety and tolerability of the combination of atorvastatin with antichagasic therapy by monitoring the incidence of adverse events and treatment discontinuation. This study will be conducted with a sample size of 300 adult patients in four centers of the National Program for the Control of Chagas Disease (PCC): 1) Hospital San Juan de Dios and 2) Hospital Felix Bulnes in Santiago, Metropolitan Region, and 3) Hospital Dr. Gustavo Fricke in Viña del Mar and 4) Hospital San Martin de Quillota, Valparaiso Region, coordinated by Drs. Edurne Urarte, Marisol Denegri and Litzi Villalon, respectively.

Adults with chronic CD are the population that most urgently requires new treatments since the most significant burden of the disease is found in these patients. Thus, improving host triggers, such as

inflammation, endothelial activation, and cardiac function, with the addition of ATO may increase the efficacy of conventional antichagasic therapy.

Given the paucity of evidence on treating this disease, this study will offer the opportunity to evaluate a novel combined therapeutic strategy on promising candidate markers of inflammation, endothelial activity, and cardiac function and to correlate the results with parasitological findings. This trial could be the first step toward evaluating a potential new therapy proposed to modify the course of chronic Chagas disease favorably.

**NUMBER OF STUDY CENTERS:**

Four centers:

1) Hospital San Juan de Dios and 2) Hospital Felix Bulnes in Santiago, Metropolitan Region, and.

3) Dr. Gustavo Fricke Hospital in Viña del Mar and 4) San Martín Hospital in Quillota, Valparaíso Region.

These four centers are part of the National Program for the Control of Chagas Disease.

**Duration of participation:** Each subject will participate in the trial for approximately 12 months from signing the informed consent form. After a screening phase of about 14 days, subjects who meet the inclusion criteria and have no exclusion criteria will be randomized to one of the three study groups.

**Duration of the study:** The total duration of patients' participation in the trial will be twelve months, considering two weeks for screening and evaluation according to PCC guidelines and eight weeks of treatment with antichagasic drugs in their respective groups. The administration of ATO and placebo will continue for an additional eight weeks. There will be follow-up visits up to 12 months after initiation of treatment.

**Sample size:** The trial contemplates a sample of 300 patients, randomly assigning 100 patients to each study group:

Atorvastatin 40 mg;

Atorvastatin 80 mg;

Placebo (nutritional supplement in tablets)

**Inclusion Criteria:**

Adults older than 18 years and younger than 50, weighing more than 40 kg, with a conventional confirmatory serology for T. cruzi infection from the Chilean Institute of Public Health (ISPCH) or an authorized reference center, and a positive qPCR will be selected to participate in this trial. Only patients with newly diagnosed Chagas disease in the chronic indeterminate phase will be included.

In addition, they must meet ALL of the criteria below to enter the study:

- Each subject must be over 18 and under 50 years of age.
- Each subject must have a positive serology for T. cruzi confirmed by the Chilean Institute of Public Health (ISPCH) or an authorized reference center
- Have a positive qPCR test for T. cruzi
- Have normal laboratory test values for the following parameters: total white blood cell count, platelet count, creatine kinase (CK), alanine aminotransferase (ALT), aspartate aminotransferase (AST), total bilirubin or creatinine, or a gamma-glutamyl transferase (GGT) <= 2 times the upper limit of normal (X ULN);
- Women of reproductive age must have a negative serum pregnancy test, not be breastfeeding, and

consistently use a highly effective method of contraception throughout the treatment phase.

- Have the ability to comply with all tests and follow-up visits specified in the protocol and have a permanent address;
- Sign the Informed Consent Form

**Exclusion Criteria:**

- Signs and symptoms of the digestive form of CD;
- Chronic cardiac CD stage II or higher;
- Acute or chronic health conditions such as acute infections, history of HIV infection, diabetes, liver and kidney disease;
- Hypothyroidism
- Family history of muscle disorders
- Pre-existing heart disease unrelated to Chagas disease;
- Formal contraindication to receive NFX or BZD,
- Known history of hypersensitivity, allergy, or severe adverse reactions to ATO, BZD, or NFX;
- History of previous treatment for CD;
- History of prior treatment with atorvastatin, lovastatin, rosuvastatin, simvastatin, or any other statin;
- Any concomitant use of antimicrobial agents;
- History of alcohol or drug abuse;
- Any condition that precludes oral medication;
- Concomitant or intended use of CYP3A4 modifiers;
- Medical history of familial short QT syndrome or concomitant therapy with medications that may shorten the QT interval.
- Abnormal laboratory test values for the following parameters: total white blood cell count, platelet count, creatine kinase (CK), alanine aminotransferase (ALT), aspartate aminotransferase (AST), total bilirubin or creatinine, or a gamma-glutamyl transferase (GGT) > 2 times the upper limit of normal (X ULN);
- Being pregnant or breastfeeding
- Refusing to use a highly effective contraceptive method during the treatment phase.

| **Prohibited drugs before administering study treatments and during the study** | **Waiting period[a]** |
|---|---|
| Systemic antifungal therapy. | 30 days |
| Other experimental drugs (new chemical or biological entities). | 30 days |
| Drugs with a known interaction with statins that could cause potentially dangerous adverse events: fibrates such as gemfibrozil, cyclosporine, and fenofibrate. | 24 hours |
| Proton pump inhibitors.[b] | 10 days |
| Drugs that modify statin plasma levels: carbamazepina, eritromicina, claritromicina, colchicina, rifampicina, hierba de san Juan (*Hypericum perforatum*). | 24 hours |
| Immunosuppressants or supraphysiologic doses of glucocorticoids.[c] | 24 hours |

CYP3A4 = P450 cytochrome 3A4; HMG-CoA= beta-hydroxy-beta-methyl-glutaryl-CoA

[a] These waiting times must be observed before initiation of study treatment. Concurrent use of these drugs is not permitted. The principal investigator must approve changes to these periods before using the study drugs or the prohibited agent.

[b] $H_2$ antagonists are allowed.

[c] Except for the short-term treatment of asthma, chronic obstructive pulmonary disease (COPD), airway hyperresponsiveness or hypersensitivity reactions, or allergic rashes.

**INVESTIGATIONAL PRODUCTS; DOSAGE, AND METHOD OF ADMINISTRATION**

**INVESTIGATIONAL PRODUCTS:**

Atorvastatin is administered orally once daily in doses of 40 or 80 mg.

**REFERENCE PRODUCTS:**

Placebo orally once daily. Placebo is justified as long as the patient receives complete conventional antichagasic therapy, and therefore, management of his Chagas disease is assured.

**Antichagasic therapy (nifurtimox or benznidazole) will be administered to all study subjects, regardless of the study group to which they are assigned.**

**Efficacy analysis:**

**Hypothesis**: Atorvastatin, in combination with conventional antichagasic therapy of nifurtimox or benznidazole, improves cardiac function in subjects with asymptomatic chronic-phase Chagas disease compared with placebo.

For analysis of the **primary outcome**, a one-sided Fisher's exact test of the proportion of patients with a significant decrease in biomarker levels in the ITT (primary analysis), and per protocol (secondary analysis) populations will be used. ANOVA test will be performed to analyze differences in variances between the different markers.

An exact test will be performed for all secondary comparisons of proportions between ATO versus placebo, including parasitological response to treatment. Latent class and multivariate analyses will assess parasitological response, biomarker changes, and ATO dose association.

If there are no significant differences between ATO treatments, these data will be collapsed into a single comparison group to obtain greater precision in the treatment effect estimate, given that this is a proof-of-concept study for statins as a class. In this case, no adjustment for multiple comparisons will be made.

**Safety Analysis**: Clinical assessments, electrocardiograms, and laboratory tests will be used to monitor the safety and tolerability of the drugs under study throughout the observation period of the subjects.

The proportion of patients presenting at least one AE will be described. The incidence rate and 95% confidence interval per study group will be presented. Otherwise, only descriptive statistics will be presented. Laboratory safety parameters (hematology and biochemistry) will also be described individually for each study group, showing the proportion of patients by degree of elevation relative to ULN and to baseline values and changes in blood levels over time.

**Experimental Design Diagram**

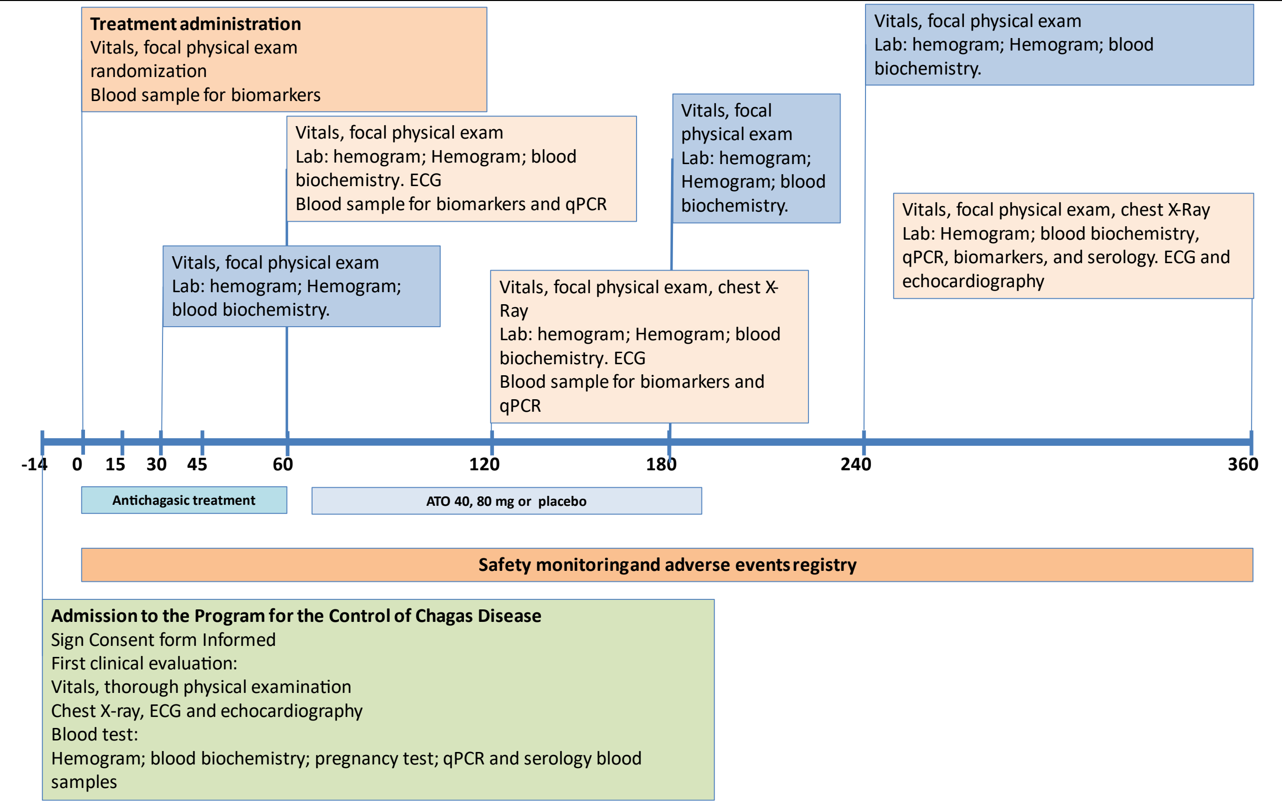


## Study Flowchart

| Phase | Pre-Randomization | | Post-Randomization | | | | | |
|---|---|---|---|---|---|---|---|---|
| **Period** | **Screening** | **baseline** | **treatment** | | | | **Follow-up** | |
| **Visit** | 1 | 2 | 3 | 4 | 5 | 6 | 7 | 8 |
| **Day(s)** | -14 a -1 | 0 | 30 | 60 | 120 | 180 | 240 | 360 |
| **Procedure** | | | | | | | | |
| Informed Consent | x | | | | | | | |
| Randomization | | x | | | | | | |
| Clinical Record | x | | | | | | | |
| Previous/concurrent drugs | ←-----------------------------------------------------→ | | | | | | | |
| Inclusion/Exclusion | x | x | | | | | | |
| Vital Signs | x | x | x | x | x | x | x | x |
| Complete physical exam | x | | | | x | | | x |
| Chest X-ray | x | | | | x | | | x |
| ECG | x | | | x | x | (x)[e] | (x)[e] | x |
| 2D Echocardiogram | x | | | | | | | x |
| Pregnancy test | x | | | | | x | x | |

| | | | | | | | | |
|---|---|---|---|---|---|---|---|---|
| Serology | x | | | | | | | x |
| Laboratory[a] | x | x | x | x | x | x | x | x |
| Quantitative PCR | x | | | | X[d] | | | x |
| Biomarkers[b] | | X | | x | x | | | x |
| Focused Physical Examination [c] | x | x | x | x | x | x | x | x |
| Adverse Events | | | ←--------------------------------→ | | | | | |
| Drug Accounting | | x | x | x | x | | | |

[a]Laboratory parameters will include: hemoglobin, total white blood cell count, differential white blood cell count, and platelet count. Laboratory biochemical parameters will include: CK, ALT, AST, GGT, alkaline phosphatase, total and direct bilirubin, a lipid profile: total cholesterol, c-HDL, and c-LDL, triglycerides, fasting blood glucose,e and creatinine;
[b]Biomarkers: BNP, cTnT, IFN-γ, IL-1β, IL-4, IL-17A, e IL-10, sICAM-1, sVCAM-1, sE-selectin;
[c]Physical examination focused only on the evaluation of adverse events.;
[d]PCR testing at these time points will be done with a single 10 ml sample;
[e]ECGs will be performed at these visits only in case of previously identified abnormalities

## Contents

**1. Project code** ........ 1
**2. Sponsor/Founder** ........ 1
**3. Clinical trial title** ........ 1
**4. Research Team** ........ 1
**6. SUMMARY** ........ 2
**7. Rationale and background** ........ 11
**7.1. Chagas Disease** ........ 11
**7.1.1. Clinical aspects of the acute and chronic phases of CD** ........ 11
**7.1.2. Diagnosis** ........ 11
**7.1.3. Chagas Disease is an inflammatory disease** ........ 12
**7.1.3.1. Parasite-dependent immune-mediated myocardial damage** ........ 12
**7.1.3.2. Microvascular involvement in CD** ........ 13
**7.1.3.3. Alterations of regional perfusion** ........ 13
**7.1.3.4. Endothelial dysfunction** ........ 13
**7.1.4. Treatment of chronic chagasic cardiomyopathy and strategies of improvement** ........ 14
**7.2. Role of statins in the inflammatory process during chronic Chagas disease** ........ 14
**8. OBJECTIVE AND GOALS OF THE TRIAL** ........ 16
**8.1. Primary Goal** ........ 16
**8.2. Secondary goals** ........ 17
**9. Study design** ........ 17
**9.1. Outcomes** ........ 17
**9.2. Statistical analysis** ........ 18
**9.3. Study description** ........ 18
**9.4. Study population** ........ 19
**9.5. Duration of the study and duration of subject participation** ........ 19
**9.6. Event Schedule for each phase during enrollment** ........ 19
**9.7. Assessments** ........ 20
**9.8. Trial design diagram** ........ 21
**10. SELECTION AND WITHDRAWAL OF SUBJECTS** ........ 22
**10.1. Inclusion criteria** ........ 22
**10.2. Exclusion criteria** ........ 22

**11. Subject treatments** ........ 23
**11.1.1. Doses and treatments regimens** ........ 23
**11.1.2. Prohibited drugs before administering study treatments and during the study** ........ 24
**11.2. Adverse events and withdrawal from the study** ........ 24
**11.2.1. Adverse events** ........ 24
**11.2.2. Serious adverse event (SAE)** ........ 25
**11.2.3. Rating of the severity of adverse events** ........ 25
**11.2.4. Evaluation of the causality of adverse events** ........ 26
**11.2.5. Withdrawal from the study** ........ 26
**11.2.6. Blind opening procedure** ........ 27
**12. Data analysis and statistical methods** ........ 27
**12.1. Sample size** ........ 27
**12.2. Randomization and treatment allocation** ........ 27
**12.3. Populations to be analyzed:** ........ 28
**12.4. Efficacy analysis** ........ 28
**12.5. Safety analysis:** ........ 28
**12.6. Criteria for early termination of the study** ........ 28
**13. Ethics** ........ 29
**14. Informed consent process** ........ 29
**14.1. Patient Costs** ........ 29
**15. Direct Access to Data and Source Documents:** ........ 29
**16. Quality Control and Quality Assurance** ........ 29
**16.1. Case Registration Forms (CRF)** ........ 30
**16.2. Source documents** ........ 30
**17. Data management and archiving of records** ........ 31
**18. Reports and publications** ........ 31
**19. References** ........ 32

## 7. Rationale and background:

We postulate that statins, such as atorvastatin, due to their anti-inflammatory effect, improve vascular and cardiac inflammation in patients with asymptomatic chronic Chagas disease, contributing to the recovery of endothelial and cardiac function and improving the efficacy of antichagasic drug treatment of Chagas disease.

### 7.1. Chagas Disease:

Significance of the problem: The flagellated protozoan *Trypanosoma cruzi* is the etiologic agent of Chagas disease (CD). This parasite is transmitted to humans by insect vectors (Triatoma infestans, aka 'vinchuca'), transfusion of infected blood, or from an infected mother to her child, which is currently the main route of transmission in Chile (MINSAL, 2017). It is a disease that affects 7 million people in 21 endemic countries in Latin America and is increasing in non-endemic countries due to migration (WHO, 2015). CD is the second disease with the highest burden among Tropical Diseases (WHO, 2011), with disability-adjusted life years (DALYs) of 806,170. Recent studies estimate that the global economic cost of CD worldwide exceeds USD$7 billion annually (Requena-Méndez et al., 2017). This figure exceeds that of rotavirus (USD$ 2 billion) and cervical cancer (USD$ 4.7 billion) (Lee et al., 2013; Schmunis, 2013). CD causes approximately 8,000 deaths annually, higher than malaria in the Americas (WHO, 2018). The primary CD control strategy includes vector control, systematic screening of blood donors in all endemic countries, detecting and treating congenital transmission, and treating infected children and acute cases (WHO, 2015). However, control programs are discontinuous and current therapy is limited due to low efficacy (WHO, 2015). This is the WHO goal of eradicating or controlling CD by 2020 was far from being achieved (Lenk et al., 2018).

#### 7.1.1. Clinical aspects of the acute and chronic phases of CD:

Clinically, CD presents an acute phase, diagnosed in less than 10% of cases, due to the absence or presence of mild symptoms. The clinical course, in most cases, is towards spontaneous recovery (Berna, 2015), but without the elimination of parasites. The chronic phase may be asymptomatic (indeterminate), characterized by positive serology and some degree of cardiac involvement evidenced by minimal but persistent inflammation. However, after 10 to 30 years, in about 30% of patients, it progresses to symptomatic forms of the disease, with involvement of the esophagus, colon, or heart (Rassi et al., 2010). Physical symptoms and signs of chronic chagasic cardiomyopathy (CCC) arise from heart failure, cardiac arrhythmias (such as ventricular arrhythmias, atrioventricular block, supraventricular tachycardia, or atrial fibrillation) (Jefferies and Towbin, 2010) and arterial or venous thromboembolism (Rassi et al., 2017a).

#### 7.1.2. Diagnosis:

The clinical diagnosis of CD in the acute phase is difficult as most infected persons have no symptoms. However, detecting the parasite in the patient's blood is possible by identifying trypomastigotes in the leukocyte coat or by conventional polymerase chain reaction (PCR). In the chronic phase, diagnosis is based on clinical and epidemiological data and serological tests such as a high-sensitivity IgG ELISA and IgG indirect immunofluorescence (Jercic and Oyarce, 2019; MINSAL, 2017). If gastrointestinal involvement is suspected, abdominal radiological studies should be

performed. Cardiac evaluation is essential when making the diagnosis to provide a functional classification and stratify a 10-year mortality risk (Nunes et al., 2018; Rassi et al., 2006; Rassi, 2010). The functional classification helps to explain the evolution of CD. It is based on clinical criteria, NYHA functional classification, chest X-ray, electrocardiogram (ECG), echocardiogram, Holter, thromboembolism, and sudden death criteria and describes stages I to IV (Rassi, 2010). The ECG is useful to detect conduction disturbances, such as atrioventricular or right bundle branch block, which may appear before symptoms manifest (Rojas et al., 2018). A 24-hour Holter should be performed in case of arrhythmias. A chest X-ray is helpful to determine the cardiothoracic index indicative of cardiomegaly. At least an echocardiographic evaluation is also recommended, especially when the ECG is abnormal (MINSAL, 2017).

Different biomarkers have been proposed to assess progression, prognosis, or response to treatment; however, none have demonstrated sufficient specificity to be incorporated as the gold standard for diagnosing Chagas disease (Cortes-Serra et al., 2020). However, brain natriuretic peptide (BNP) and cardiac troponin T (cTnT) have been proposed as useful biomarkers to predict the progression of left ventricular dysfunction (Echeverría et al., 2020). On the other hand, parasite DNA detection is a valuable marker only to determine treatment failure, as a negative result does not exclude parasite persistence (Alonso-Padilla et al., 2017; Parrado et al., 2019).

#### 7.1.3. Chagas Disease is an inflammatory disease:

The main pathogenic mechanisms explaining CCC are i) parasite-dependent immune-mediated myocardial damage, which is the most critical determinant of the disease (Rassi et al., 2017b), where the TH1/TH2/TReg response is a crucial feature (Acevedo et al., 2018); ii) microvascular abnormalities and ischemia (Borges et al., 2018); and iii) cardiac dysautonomia secondary to autoreactive immune mechanisms (Booney et al., 2019).

##### 7.1.3.1. Parasite-dependent immune-mediated myocardial damage:

During primo-infection, shortly after macrophage invasion, IL-12/TNF-α-stimulated NK cells produce a peak of IFN-γ secretion to control parasite replication at the expense of decreasing IL-10 production (Cardillo et al., 1996). IL-12 production further contributes to polarizing the immune response towards a wide diversity of pro- and anti-inflammatory profiles (Hasegawa et al., 2002), which explains, in part, the evasion of the immune response.

Due to the parasite's persistence, the host's fate is toward a low-grade but permanent state of immune system activation. In such a scenario, the T-cell response is essential for maintaining the typically low parasitemia in the chronic phase of the disease. However, CCC has a defective cytotoxic T cell response with the gradual depletion of the CD8+ T cell population (Perez-Anton et al., 2020). Furthermore, the TH1/TH2 balance is essential for progression to CCC. The parasite induces a combined response where the balance between excess proinflammatory (IFN-γ, TNF-α, IL-1β) and anti-inflammatory (IL-4, IL-10) cytokines may be critical in the development of CCC (Acevedo et al., 2018). Special mention deserves the TH17 subset as elevated levels of IL-17A may correlate with milder heart disease (Sousa et al., 2017).

*From the above, IFN-γ, IL-1β, IL-4, IL-17A, and IL-10 levels could help predict CD progression and outcome after drug therapy (Llaguno et al., 2019), and the inclusion of anti-inflammatory drugs could favorably incline toward a better immune response profile and improve treatment of CCC.*

#### 7.1.3.2. Microvascular involvement in CD:

Experimental and clinical evidence exists of coronary microvascular alterations leading to ischemic myocardial damage in *T. cruzi*-infected animals and patients with CCC (Rossi et al., 2010). It has been reported by the authors and others that microvascular alterations related to i) regional perfusion alterations (Lemos de Oliveira et al., 2018), ii) platelet activation (González-Herrera et al., 2017; Pengue et al., 2019), and iii) endothelial dysfunction (Campos-Estrada et al., 2015) in mouse hearts chronically infected with T. cruzi, are similar to those observed in ischemic cardiomyopathies (Borges et al., 2018).

#### 7.1.3.3. Alterations of regional perfusion:

Up to 20% to 30% of patients with CCC complain of chest pain that resembles angina pectoris in location and character, unrelated to exertion, and with healthy subepicardial arteries (Lemos de Oliveira et al., 2018). In addition, there are reversible myocardial perfusion defects, which can be reversed with dipyridamole (Tanaka et al., 2019). These defects can be attributed to inflammation around intramural coronary arteries causing redistribution of coronary flow from ischemic to non-ischemic regions, thus inducing the appearance of the clinical picture consistent with coronary microvascular dysfunction (Bestetti and Restini, 2014). This aspect may be correlated with autonomic denervation (Barizon et al., 2020), even before the appearance of fibrosis.

#### 7.1.3.4. Endothelial dysfunction:

Impaired perfusion may directly result from microvascular damage secondary to endothelial activation induced by *T. cruzi* infection and subsequent interaction with immune effector cells. Activated endothelial cells (ECs) produce various cytokines, including the vasoconstriction-inducing molecules endothelin-1 and thromboxane A2 (TXA2) (Hernandez et al., 2018). As we previously demonstrated, ECs also increase the production of cell adhesion molecules such as Intercellular Cell Adhesion Molecules type 1 (ICAM-1), Vascular Cell Adhesion Molecules (VCAM), and E-selectin, including a soluble form of cell adhesion molecules (sCAM) found in blood (Campos-Estrada et al., 2015; Gonzalez-Herrera et al., 2017; Molina-Berrios et al., 2013b). We also demonstrated that the induction of adhesion molecules is mediated by the activation of inflammatory signaling cascades (Campos-Estrada et al., 2015), amplifying the initial inflammatory input. In addition, cytokine production from immune and activated EC facilitates the migration of inflammatory cells and monocytes to the subendothelium, where they produce a local inflammatory response, increasing endothelial damage (González-Herrera et al., 2017; Marín-Neto et al., 2013) and the perfusion alterations mentioned above. All these molecules are potential biomarkers for predicting thrombotic or endothelial disorders (Cortes-Serra et al., 2020). In particular, E-selectin may be an excellent candidate, as it is exclusively expressed in vascular ECs (Dzikowska-Diduch et al., 2017).

Therefore, myocardial perfusion disorders, due to endothelial damage and microcirculatory alterations, contribute to the progression of LV segmental dysfunction observed in the chronic phase of CD (Hiss et al., 2009). Any therapeutic approach to improve left ventricular function or prevent these vascular alterations could benefit patients with CD.

*Overall, considering the role of the endothelium in initiating and propagating vascular wall injury in CD, it is necessary to evaluate pharmacological treatments capable of inhibiting EC activation, dysfunction, and vascular damage in patients with chronic CD.*

#### 7.1.4. Treatment of chronic chagasic cardiomyopathy and strategies of improvement:

In Chile, etiologic treatment of CD is with 5-10 mg/kg/day of nifurtimox (NFX) or 5 mg/kg/day of benznidazole (BZD) for 60 days (MINSAL, 2017). All patients should receive antichagasic treatment without exclusion or delay, except during pregnancy and lactation, although there is recent evidence suggesting that NFX may be safe in these conditions (Moroni et al., 2019). Also, hypersensitivity to trypanocidal drugs excludes its use. Other exclusions are severe renal, hepatic, or cardiac disease, including CCC with structural compromise, alcoholism (Antabuse effect of NFX), and severe megacolon or megaesophagus (MINSAL, 2017).

Treatment of CD is associated with an increased risk of serious adverse events (AEs), with a mean incidence of 16.20% (PAHO, 2018), a situation that may force discontinuation of therapy in up to 48% of patients receiving NFX (Crespillo-Andujar et al., 2018a; Crespillo-Andujar et al., 2018b; Jackson et al., 2020). AEs include weight loss greater than 5-15%, digestive disorders (nausea, vomiting, diarrhea, epigastralgia and dyspepsia, abdominal distension), headaches, irritability, insomnia, mood disorders, memory loss, fever, arthralgias, myalgias, severe or significant skin and mucosal rashes such as Stevens-Johnson syndrome, evidence of thrombocytopenia, pancytopenia or signs of bone marrow depression, elevated transaminases, leukopenia below 2500/mm3, CNS disorders such as paresis, paralysis or seizures, and behavioral changes, especially in the case of NFX.

Pharmacological treatment during the acute phase, congenital disease, and early indeterminate phase has satisfactory efficacy and is considered curative (Sales Junior et al., 2017). However, it is more difficult to declare a cure for chronic infection because the current evidence for drug efficacy in this phase is weak or controversial, especially when mortality is considered (PAHO, 2018). After five years of follow-up, BZD could not significantly reduce clinical cardiac deterioration in patients with mild heart disease (Morillo et al., 2015). Moreover, long-term follow-up is required to confirm seroreversion, making serology a weak surrogate as a criterion for cure in chronic CD (Sguassero et al., 2018). Novel therapies, such as the antifungal drugs posaconazole or ravuconazole, were not successful (Molina et al., 2014; Molina et al., 2017; Torrico et al., 2018). Thus, rigorous clinical trials have not demonstrated convincing evidence of clinical benefit or eradication of parasitemia in chronic CD.

Therefore, finding innovative approaches to treat this neglected life-threatening tropical disease is paramount. Three considerations support this claim: i) current therapy for CD in the chronic phase is unsuccessful and increases the risk of systemic toxicity, AE, and poor adherence to treatment or noncompliance; ii) none of the many potential trypanocidal compounds listed is superior to NFX or BZD (Ribeiro et al., 2020); and iii) most antiparasitic drugs are considered orphan drugs due to the low return of development costs to the pharmaceutical industry (Varela and Fernandes, 2020). *Therefore, improving the efficacy of current antiparasitic drugs by modifying host factors such as the inflammatory response - mainly by repurposing existing drugs - would make current treatment more effective*.

### 7.2. Role of statins in the inflammatory process during chronic Chagas disease

There are molecules involved in the natural resolution of inflammation. These specialized pro-resolution mediators include several lipids that control the magnitude and duration of local inflammation (Serhan and Chiang, 2013). These lipids are derived from essential fatty acids in the

plasma membrane, such as arachidonic acid or docosahexaenoic acid. Interestingly, aspirin and cholesterol-lowering statins, including atorvastatin (ATO), can S-nitrosylate the enzyme cyclooxygenase-2 (COX2) at amino acid 298, which allows it to produce precursors no longer for the synthesis of prostaglandins (which are pro-inflammatory), but epimers that, through the action of 5-lipooxygenase (5-LO), will give rise to more stable molecules, such as 15-epi-lipoxin A4 (LXA4) or aspirin-activated resolvin D1 (AT-RvD1)(Serhan, 2017; Spite and Serhan, 2010).

The responsible investigator and his team conducted several preclinical studies with results suggesting that aspirin and simvastatin decrease cardiac inflammation and endothelial activation in *T. cruzi* chronically infected mice (Lopez-Munoz et al., 2010; Molina-Berrios et al., 2013b; Molina-Berrios et al., 2013c). 15-epi-LXA4 mediates this action (Campos-Estrada et al., 2015; González-Herrera et al., 2017; Molina-Berrios et al., 2013a) in a process that involves a decrease in the activity of the proinflammatory NF-κB pathway (Campos-Estrada et al., 2015). Furthermore, simvastatin improves, independently of 15-epi-LXA4 activity, ventricular function in chronically infected BALB/c mice (Guzmán-Rivera et al., 2020), while ongoing studies show that aspirin/statin-triggered resolvin D1 (AT-RvD1) improves cardiac electrical function, in the absence of trypanocidal treatment.

It follows that *a combination of trypanocidal drugs and those that induce the resolution of the inflammatory process derived from the persistence of the parasite could be an excellent therapeutic strategy to prevent the chronic consequences of CD*.

Although the preclinical evidence suggests that a statin could benefit chronic CD, this matter has not been tested in the clinical setting. There is only one mention of the use of rosuvastatin in the treatment of CD, but the quality of the evidence provided is poor (Marti-Carvajal and Kwong, 2016). Other clinical trials that focused only on symptomatic treatment of heart failure associated with CD have tested amiodarone (Stein et al., 2018), carvedilol (Botoni et al., 2007), and angiotensin-modulating drugs (Souza-Silva et al., 2019). Other drugs studied in small clinical trials are allopurinol, itraconazole, or ketoconazole, with mixed results (Apt et al., 1998; Apt et al., 2013; Brener et al., 1993); unfortunately, more clinical trials with these drugs are lacking. A major obstacle to drug development is the poor translation of in vivo data to human disease. **Thus, our proposal aims to analyze whether a statin improves the efficacy of antichagasic therapy in the context of Chronic Chagas Disease through a double-blind phase II clinical trial and to evaluate the inflammatory parameters associated with the observed improvement**.

There is widespread agreement that adults with chronic indeterminate CD are the population with the most urgent need for developing new treatments due to the increased disease burden for these patients. Thus, improving host factors (e.g., provoked immune reaction) may increase the efficacy of conventional antichagasic therapy, probably by decreasing doses, reducing their duration, or both.

Given the paucity of evidence-based treatments for this disease, this study will provide an opportunity to assess the impact of a novel therapeutic strategy on a combination of promising candidate markers of inflammation, endothelial activity, and cardiac function associated with sustained improvement and correlate the results with parasitological outcomes. *Thus, this trial*

*could be the first step in evaluating a potential therapy proposed to modify the course of chronic indeterminate CD favorably*.

Although our preclinical studies were performed with simvastatin, we propose atorvastatin (ATO) as an experimental statin. As simvastatin is associated with ezetimibe, it will not be considered in this study. ATO's therapeutic and safety profiles are well known, as well as its mechanism of action and pharmacological actions, including its anti-inflammatory properties, which are shared by the other members of the statin class. Due to the low incidence of severe AEs and its efficacy, ATO is one of the most widely used statins. 20-80 mg/day of ATO are used to lower the so-called LDL cholesterol, implicated in the pathogenesis of the atherosclerotic cardiovascular disease. However, a clinical phase II proof-of-concept study is imperative because i) it is a way to translate the results obtained in the animal model, and ii) a minimum effective dose and safety need to be evaluated in the context of the patient undergoing treatment of chronic CD and, most importantly, iii) because statins are not currently approved for the treatment of CD.

Considering i) the high burden of disease; ii) the role of the endothelium and inflammation in the initiation and propagation of vascular wall injury in CD; iii) that current therapy for CD in the chronic phase is unsuccessful and increases the risk of systemic toxicity, AEs, and poor compliance; iv) that improving the efficacy of current antichagasic drugs by modifying host responses would make current treatment more effective; v) that preclinical evidence suggests that statins may have a role in preventing cardiac damage in CD; vi) that statins are safe and with a well-known pharmacological profile; then, a combination of trypanocidal drugs with those that induce resolution of the inflammatory process resulting from parasite persistence could be an effective therapeutic strategy to prevent the chronic consequences of CD. Consequently, it is proposed that the following **hypothesis**:

**In patients with chronic Chagas disease, statins improve the efficacy of antiparasitic treatment by decreasing inflammation and improving endothelial and cardiac function**

## 8. OBJECTIVE AND GOALS OF THE TRIAL

### 8.1. Primary Goal:

To evaluate the use of atorvastatin, in combination with antiparasitic therapy (NFX or BZD), is safe and more effective than antiparasitic therapy alone in preventing the occurrence of cardiac disturbances by reducing overall inflammation and in improving endothelial and cardiac functions.

To assess the primary endpoint, the number of patients with a change in the stage of chronic cardiomyopathy in 12 months from the beginning of antichagasic treatment will be considered.

We will evaluate whether the effect of atorvastatin in combination with antiparasitic treatment (NFX or BZD) is more effective than antiparasitic therapy alone (placebo group) in preventing the appearance of cardiac disorders determined by non-progression from stage A according to the First Latin American Guidelines for the diagnosis and treatment of chagasic cardiomyopathy (Andrade et al, Arq Bras Cardiol 2011;97 Suppl 3:1-48.) To make this assessment, significant changes in i) electrocardiogram (heart rate and QT interval duration, as well as the appearance of electrical conduction disorders, determined by QRS segment duration and morphology), ii) ejection fraction

(assessed by echocardiography), and iii) cardiac silhouette size (chest X-ray) will be taken into account

**8.2. Secondary goals:**

8.2.1. To evaluate the efficacy of the combination of ATO and antichagasic therapy to decrease:

- Inflammation, as measured by plasma levels of the cytokines, TNF-α, IFN-γ, IL-10, IL-1B, IL-4, and IL-17A.
- Endothelial activation, as measured by plasma levels of sCAM: sE-selectin, sICAM-1, and sVCAM-1.
- Cardiac damage and function: measured by plasma levels of BNP, cTnT, 12-lead resting ECG (heart rate, QT segment duration, and electrical conduction changes determined by QRS segment duration and morphology), and 2D echocardiogram (ejection fraction).

8.2.2. To determine the safety and tolerability of the combination of ATO with antichagasic therapy, measured by the incidence of AEs (e.g., rhabdomyolysis) and treatment discontinuation.

8.2.3. To evaluate the treatment response to the combination of ATO with antichagasic therapy, as measured by quantitative PCR and serology, over a 10-month follow-up period.

8.2.4. To determine the degree of therapy adherence by measuring drug accountability throughout the study.

## 9. Study design:

### 9.1. Outcomes:

As in any clinical trial, the results should be clearly defined beforehand. Thus, the **primary outcome** of this study at 12 months is the change in cardiac function in patients with chronic CAD. To this end, the number or proportion of patients presenting a change in the phase of chronic cardiomyopathy will be assessed. This phase change is determined by non-progression or reversion from or to phase A, according to the I Latin American Guidelines for the diagnosis and treatment of chagasic cardiomyopathy (Andrade et al., 2011), considering the consistent findings of structural alteration evidenced by changes in clinical, electrocardiographic, echocardiographic parameters and, additionally, of plasma levels of the cardiac function biomarkers BNP, cTnT.

**Chart 1 – Clinical classification of left ventricular dysfunction in chagasic cardiopathy**

| Acute phase | Chronic phase | | | | |
|---|---|---|---|---|---|
| | Indeterminate form | Cardiac form with no ventricular dysfunction | Cardiac form with ventricular dysfunction | | |
| | A | B1 | B2 | C | D |
| Patients with findings compatible with acute Chagas disease | Patients at risk for developing CHF. They have positive serology, neither structural cardiopathy nor CHF symptoms. No digestive changes | Patients with structural cardiopathy, evidenced by electrocardiographic or echocardiographic changes, but with normal global ventricular function and neither current nor previous signs and symptoms of CHF | Patients with structural cardiopathy characterized by global ventricular dysfunction, and neither current nor previous signs and symptoms of CHF | Patients with ventricular dysfunction and current or previous symptoms of CHF (NYHA FC I, II, III or IV) | Patients with refractory symptoms of CHF at rest, despite optimized clinical treatment, requiring specialized interventions |

Arq Bras Cardiol 2011; 97(2 supl.3): 1-48

The **secondary outcome** is determined by the change in plasma levels of biomarkers versus changes in parasite load.

Additional measures of scientific value: (a) change in plasma levels of biomarkers of inflammation and endothelial activation; (b) incidence and severity of AEs; (c) change in parasite burden and serologic response over the follow-up period, measured by quantitative PCR (qPCR); (d) changes in biomarker levels at different follow-up time points and correlation with parasite burden and ATO regimen; and (e) incidence of treatment discontinuation due to severe AEs.

**9.2. Statistical analysis:** see section 11.
Clinical events related to CD occur at a low incidence in patients with the indeterminate CD due to the pathogenesis and hysteresis of the disease, parasite characteristics, and host response times; therefore, extensive clinical trials would be needed to assess changes in incidence. In addition, serologic and clinical endpoints of cure would require several years to decades in chronic CD (Bern, 2015). Consequently, the criterion of cure as a primary outcome, determined by seroreversion, is not considered in this proposal.

**9.3. Study description:**
This is a proof-of-concept Phase II clinical trial with three different groups: Two groups will receive antichagasic therapy plus 40 or 80 mg/day of ATO. The third group will receive a placebo plus antichagasic therapy. The placebo is justified as long as the patient receives the complete conventional antichagasic treatment; therefore, the management of his Chagas disease is assured. In this clinical trial, the placebo will be a nutritional supplement tablet with an appearance similar to that of ATO. This product was chosen given the potential difficulties in manufacturing a small number of pills since the trial does not have a pharmaceutical industry sponsor capable of manufacturing it under Good Manufacturing Practice standards.

Nutritional supplements do not affect vascular or cardiac inflammatory parameters and therefore do not introduce confounding elements in the comparative analysis with atorvastatin.

Patients will first receive conventional antichagasic therapy for two months. After two weeks (or until normalization of liver enzymes), treatment with atorvastatin or placebo will be initiated for

four months. These doses were chosen to minimize false negative results, thus providing the best hypothesis test and maximizing the pharmacodynamic effect on inflammation and cardiac and endothelial function. In addition, the treatment sequence aims to minimize adverse reactions that could be caused by the eventual combination of antichagasic drugs with atorvastatin. Study subjects will be randomized, and the three groups will be double-blinded (both clinical investigators and subjects will be blinded) to ATO or placebo. However, antichagasic therapy will be open-labeled. In addition, qPCR and the other laboratory evaluations, as well as ECGs and 2D echocardiograms, will be performed with the clinical investigators blinded to ATO or placebo allocation.

This study will be conducted in four centers: 1) Hospital San Juan de Dios and 2) Hospital Felix Bulnes in Santiago, Metropolitan Region, 3) Hospital Dr. Gustavo Fricke in Viña del Mar, 4) Hospital San Martin de Quillota, Valparaiso Region. Each center annually admits 60 new patients to its Chagas Control Program (CCP). These four centers are part of the care network of the National Chagas Disease Control Program.

**9.4. Study population:**
Patients older than 18 years and younger than 50 with indeterminate chronic CD attending the PCC will be enrolled equally and randomly into the four study groups. According to PCC guidelines, patients older than 50 are not eligible for antichagasic therapy. For subject selection, see below. The choice of this target population for CD is primarily due to the unmet medical need for a new, safe and effective treatment for chronic indeterminate CD.

**9.5. Duration of the study and duration of subject participation:**
The total duration of patients' participation in the study will be twelve months, considering two weeks for screening and evaluation according to PCC guidelines and eight weeks of treatment with the antichagasic drugs. The administration of ATO and placebo will continue for an additional eight weeks in their respective groups. There will be follow-up visits up to 12 months after the start of treatment (see appendix for a graphical presentation of the study design).

Following voluntary written informed consent, patients will begin a 14-day evaluation phase. Once the patient is randomized and treatments are initiated, they will have follow-up visits during the treatment phase of the study on days 30, 60, 120, and 180, with a two-week rest period or until normalization of liver enzyme levels (± four-day allowable window), and three visits after the end of treatment (EOT) on days 180, 240 and 360 (± 14-day allowable window). In addition, patients will be advised to return on any day during the follow-up period if they have any medical incidents or AEs.

Recruitment is expected to be completed within 24 months from the start of recruitment. Therefore, the time frame between the first patient entering (FPI) and the last patient leaving (LPO) is 36 months. However, the total duration of the study is estimated at 48 months, from the initiation phase to the final study report.

**9.6. Event Schedule for each phase during enrollment:**

| Phase | Pre-Randomization | Post-Randomization |
|---|---|---|

| **Period** | **Screening** | **baseline** | **treatment** | | | | **Follow-up** | |
|---|---|---|---|---|---|---|---|---|
| **Visit** | 1 | 2 | 3 | 4 | 5 | 6 | 7 | 8 |
| **Day(s)** | -14 a -1 | 0 | 30 | 60 | 120 | 180 | 240 | 360 |
| **Procedure** | | | | | | | | |
| Informed Consent | x | | | | | | | |
| Randomization | | x | | | | | | |
| Clinical Record | x | | | | | | | |
| Previous/concurrent drugs | ←-------------------------------------------------------→ | | | | | | | |
| Inclusion/Exclusion | x | x | | | | | | |
| Vital Signs | x | x | x | x | x | x | x | x |
| Complete physical exam | x | | | | x | | | x |
| Chest X-ray | x | | | | x | | | x |
| ECG | x | | | x | x | (x)[e] | (x)[e] | x |
| 2D Echocardiogram | x | | | | | | | x |
| Pregnancy test | x | | | | | x | x | |
| Serology | x | | | | | | | x |
| Laboratory | x | x | x | x | x | x | x | x |
| Quantitative PCR | x | | | | X[d] | | | x |
| Biomarkersb | | X | | x | x | | | x |
| Focused Physical Examination c | x | x | x | x | x | x | x | x |
| Adverse Events | | | | ←-------------------------------→ | | | | |
| Drug Accounting | | x | x | x | x | | | |

[a]Laboratory parameters will include: hemoglobin, total white blood cell count, differential white blood cell count, and platelet count. Laboratory biochemical parameters will include: CK, ALT, AST, GGT, alkaline phosphatase, total and direct bilirubin, a lipid profile: total cholesterol, c-HDL, and c-LDL, triglycerides, fasting blood glucose, and creatinine;
[b]Biomarkers: BNP, cTnT, IFN-γ, IL-1β, IL-4, IL-17A, e IL-10, sICAM-1, sVCAM-1, sE-selectin;
[c]Physical examination focused only on the evaluation of adverse events.;
[d]PCR testing at these time points will be done with a single 10 ml sample;
[e]ECGs will be performed at these visits only in case of previously identified abnormalities

### 9.7. Assessments:

On admission to the PCC, patients will undergo a complete medical history with an emphasis on CD, demographic data and medication history, physical examination, body weight and height, vital signs, and body temperature.

Ten ml of blood will be collected, in separate tubes, for hematological and biochemical evaluations: hemoglobin, total and differential white blood cell count, platelet count, CK, ALT, AST, GGT, alkaline

phosphatase, total and direct bilirubin, a lipid profile: total cholesterol, c-HDL, and c-LDL, triglycerides, fasting blood glucose and creatinine. A serum pregnancy test will also be performed. This test will be performed in the clinical laboratories of each center.

A 3 mL blood sample will be collected for conventional CD serology at each center's clinical laboratories and for a confirmatory serology test to be performed at the ISPCH or an accredited laboratory.

5 mL of blood will be collected for qPCR. For clinical development and proof of concept, it has been proposed to use quantitative PCR parasitological tests as markers of efficacy in clinical studies of chronic indeterminate CD (Parrado et al., 2019). Thus, the real-time PCR technique described by Duffy et al. will be used in this assay (Duffy et al., 2013). Blood samples will be immediately added to a tube containing a volume (10mL) of a solution of guanidine/ClH 6M EDTA 0.2M 0.2M guanidine/ClH buffer 0.2M pH 8.0 (GEB) (Schijman et al., 2003). Samples with guanidine buffer can remain at room temperature for up to 30 days. For longer periods, it is necessary to store them in a refrigerator. After DNA extraction, samples will be processed using a commercial kit on an Applied 155 Biosystems 7300 RT-PCR system (Thermo Fisher).

An additional 3 ml of blood will be drawn for the biomarkers: BNP, cTnT, IFN-γ, IL-1β, IL-4, IL-17A, and IL-10, sICAM-1, sVCAM-1, sE-selectin. These markers were selected following a literature review (Cortes-Serra et al., 2020; Echeverría et al., 2020; Llaguno et al., 2019). Serum levels of these markers will be determined using a multiplex approach with a Luminex 200 (R&Dsystems). To establish biological reproducibility, a pilot study will be performed before the sample analyses of this assay.

Finally, a baseline resting ECG and a 2D echocardiogram will be performed. Both tests will be performed at each center. The ECG should be normal or with nonspecific changes (incomplete right bundle branch block, incomplete left anterior fascicular block, mild bradycardia, minor PR interval widening, and minor ST-T changes). Any clinically significant abnormality found on the electrocardiogram will automatically lead to the exclusion of the patient from this study (see exclusion criteria).

After randomization and before starting treatments, patients will undergo a new focused physical examination, and blood will be drawn for qPCR and biomarker determination.

## 9.8. Trial design diagram:

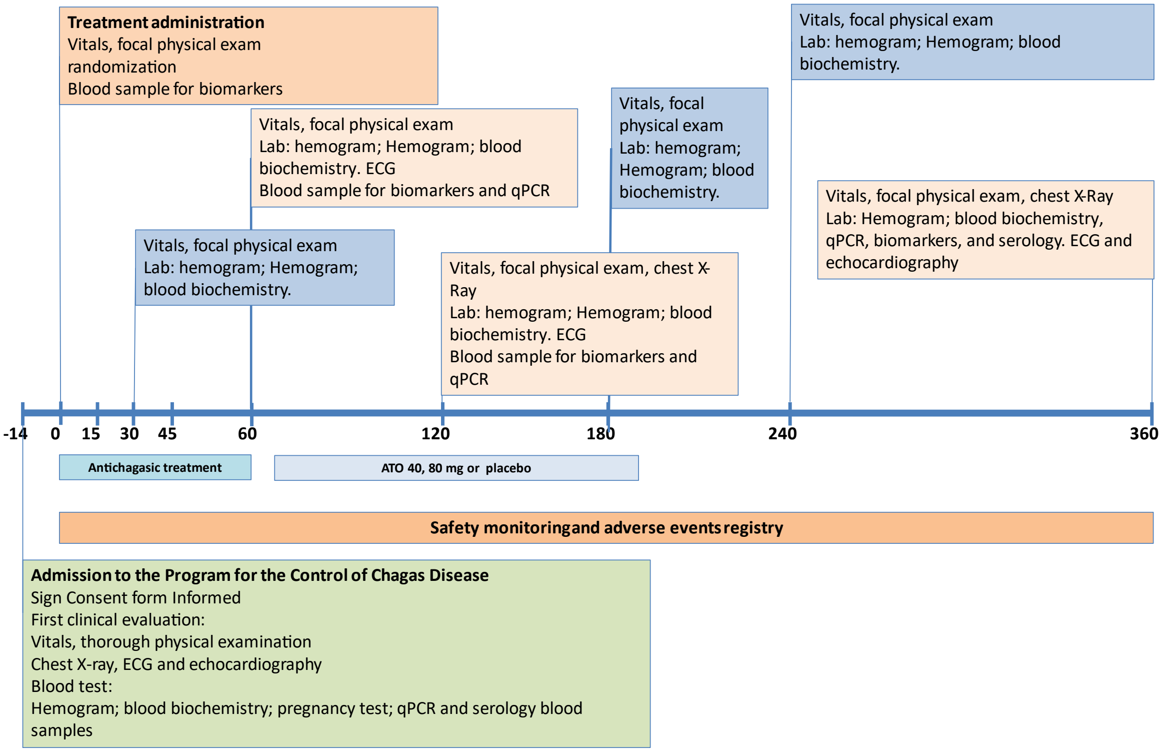


## 10.SELECTION AND WITHDRAWAL OF SUBJECTS

### 10.1. Inclusion criteria:

Subjects eligible to be enrolled in this trial must be adults older than 18 years and younger than 50 years, weighing more than 40 kg, with a conventional confirmatory serology for T. cruzi infection from the Instituto de Salud Pública de Chile (ISPCH). A positive qPCR will be selected to participate in this trial. Only patients with a new diagnosis of Chagas disease in the chronic indeterminate phase will be included. In addition, they must have normal values in laboratory tests for the following parameters: total white blood cell count, platelet count, creatine kinase (CK), alanine aminotransferase (ALT), aspartate aminotransferase (AST), total bilirubin or creatinine, or a gamma-glutamyl transferase (GGT) <= 2 times the upper limit of normal (X ULN); Women of reproductive age must have a negative serum pregnancy test, must not be breastfeeding, and must consistently use a highly effective method of contraception throughout the treatment phase.

Subjects must also meet all the following criteria: the ability to comply with all tests and follow-up visits specified in the protocol and have a permanent address; sign the written informed consent form.

### 10.2. Exclusion criteria:

Signs and symptoms of the digestive form of CD; stage II or higher chronic cardiac CD; acute or chronic health conditions such as acute infections, history of HIV infection, diabetes, liver and kidney disease; pre-existing heart disease unrelated to Chagas disease; hypothyroidism, and family history of muscle disorders; formal contraindication to receiving NFX or BZD, known history of hypersensitivity, allergy or severe adverse reactions to ATO, BZD or NFX; history of prior treatment

for CD; history of previous treatment with atorvastatin, lovastatin, rosuvastatin, simvastatin or any other statin; any concomitant use of antimicrobial agents; history of alcohol or drug abuse; any condition that precludes oral medication; concomitant or intended use of CYP3A4 modifiers; a medical history of familial short QT syndrome or concomitant therapy with drugs that may shorten the QT interval. Abnormal laboratory test values for the following parameters: total white blood cell count, platelet count, creatine kinase (CK), alanine aminotransferase (ALT), aspartate aminotransferase (AST), total bilirubin or creatinine, or a gamma-glutamyl transferase (GGT) > 2 times the upper limit of normal (X ULN); Pregnant or lactating women; refusal to use a highly effective method of contraception during the treatment phase.

Patients meeting the selection criteria will undergo the randomization procedure. Only subjects enrolled in this trial will receive the study drugs. Patients will be assigned, in ascending order, a trial identification number (TIN) according to the enrollment order. A patient is considered randomized when s/he receives a TIN.

## 11.Subject treatments

### 11.1.1. Doses and treatments regimens

Tablets of 40 and 80 mg of ATO will be purchased from a pharmaceutical laboratory with current sanitary registration recognized by the ISPCH, offering the active ingredient in a bioequivalent formulation. The placebo tablets will correspond to a nutritional supplement tablet supplied by a registered provider.

The purchase will be made through the Pharmacy of the Clinical Hospital of the University of Chile.

The ATO and placebo tablets will be repackaged in black plastic bottles of 200 ml capacity, previously sterilized with UV light, under aseptic conditions using a type II biosafety hood. The bottles will contain 120 tablets each and all from the same lot to allow the traceability of the drug.

The labeling of each bottle will be done as required by good manufacturing practices, including a) name and contact information (telephone and e-mail) of the principal investigator; b) dosage form, route of administration, number of units; c) lot number and code (assigned by the principal investigator) of the supplier laboratory; d) project code; e) instructions for use; g) randomization code; f) warning: "for clinical trial use only"; h) expiration date; i) the phrase: "Keep out of the reach of children.”

The antichagasic drugs NFX or BZD will be supplied by the Chilean Ministry of Health as part of the PCC. The antichagasic treatment will be administered according to the indications of the "Manual de Procedimientos para la Atención de Pacientes con Chagas" (MINSAL, 2017), and the administration of ATO, antichagasic drugs, and placebo treatment will be monitored at each of the patients' visits to determine tablet accounting and compliance with the drugs. BZD dosing will be 5 mg/kg/day every 12 hours (BID), and NFX dosing will be 5-10 mg/kg/day BID. ATO doses will be 40 and 80 mg/day for each group and will be administered in the morning.

At each visit, the patient will be required to bring all remaining study medications at the 30th, 60th, 120th, and 180$^{th}$-day visits to check compliance with the prescribed antichagasic and experimental treatment and perform medication accounting (Torrico et al., 2018). **If a subject develops**

**symptoms or signs of NFX intolerance, s/he can be switched to BZD without being withdrawn from the study**.

The ATO, placebo, and antichagasic drugs should not be used for purposes other than this trial.

At this point, it is necessary to indicate that if the patient manifests symptomatology suggestive of heart failure during follow-up visits, s/he will be referred to cardiological consultation for evaluation. The addition of specific therapy for managing heart failure will be recorded in the clinical record of the trial.

#### 11.1.2. Prohibited drugs before administering study treatments and during the study.

Concurrent use of systemic antifungal therapy or other experimental drugs (new chemical or biological entities) is not allowed. If the subject is on systemic antifungal therapy, s/he can only be included in the present study after a 30-day waiting period. In the case of proton pump inhibitors, the waiting period should be 10 days. The use of H2 antagonists is allowed. The concomitant use of drugs that modify statin levels such as carbamazepine, erythromycin, clarithromycin, colchicine, rifampicin, St. John's wort (Hypericum perforatum), as well as immunosuppressants or supraphysiological doses of glucocorticoids (except for short-term treatment of asthma, chronic obstructive pulmonary disease (COPD), airway hyperresponsiveness or hypersensitivity reactions or allergic rashes) is also not permitted.

### 11.2. Adverse events and withdrawal from the study

#### 11.2.1. Adverse events:

An AE will be defined as any adverse medical event (any unfavorable and unintended symptom, sign, or disease, including an abnormal laboratory or ECG finding) or worsening of any pre-existing condition occurring during the study, whether or not considered causally related to the study or study drugs. Abnormal laboratory (hematology and biochemistry) results will be reported as an AE if they: a) occur or worsen after initiation of study treatments, b) are considered a clinically significant adverse change by the clinical investigator, or c) are higher than Common Terminology Criteria for AEs (CTCEA) grade 1 (National Cancer Institute (USA), 2017), unless associated with an already reported clinical AE.

Clinical investigators, or appropriate site personnel (not involved in the study), will screen any subject experiencing an AE as soon as possible. The investigator will do whatever is medically necessary for the safety and well-being of the subject. The subject will remain under observation while receiving any trial drug and for two months following the last day of drug administration, or longer if medically indicated in the investigator’s opinion. All AEs observed or reported after administration of investigational treatments will be followed until resolved or until medically stable.

For this study, the clinical investigators at each center will report to the principal investigator, who, in turn, will be responsible for reporting AEs to the Ethics Committees of the Centers and the Human Research Ethics Committee of the Faculty of Medicine of the University of Chile and the clinical trials section of the ISPCH. Clinical investigators should report all directly observed AEs and all those spontaneously reported by subjects, using concise medical terminology. In addition, during each trial visit, subjects will be interviewed and undergo a specific physical examination for the evaluation of AEs. All adverse events should be recorded in the subject's clinical record and an ad hoc database.

### 11.2.2. Serious adverse event (SAE):

An adverse event will be defined as serious if it is

- fatal
- endangers the patient's life
- requires or prolongs hospitalization
- results in persistent or significant disability
- is a congenital anomaly/birth defect
- results in a major medical event that may not be immediately life-threatening or does not directly result in death or hospitalization but may jeopardize the patient's safety or may require intervention to prevent the other outcomes listed above.

Serious events include any other event defined as serious for the protocol's specific purposes or as serious by the ISPCH.

The clinical investigator should classify each adverse event as either serious or non-serious. This classification will determine the reporting procedure for the event.

All SAEs must be reported immediately (within 24 hours of knowledge of the SAE by the clinical investigator) to the Principal Investigator using the SAE reporting form. This includes a description of the event, date of onset and type, duration, severity, relationship to study drug, outcome, taken actions, and all other pertinent clinical and laboratory data. The initial report should be followed by submitting additional information (SAE follow-up form) as it becomes available. Follow-up reports should be submitted as soon as possible and ideally within five working days.

SAEs should also be reported on the clinical trial adverse event reporting form. It should be noted that the formulary for SAE reporting is not the same as the adverse event section of the CRF. Although the same data are collected, the two forms should be completed consistently, and the same medical terminology should be used.

Non-serious adverse events should be reported on the CRF and sent to the Principal Investigator as specified in section 14.1 of this protocol.

In addition to immediate reporting of SAEs to the Principal Investigator, investigators are responsible for reporting SAEs occurring at their site to their appropriate Clinical Ethics Committee and any periodic safety reports, following local site requirements. For this trial, the clinical investigator at each site and the Principal Investigator will assume responsibility for reporting GSDs to the ISPCH and ethics committees as necessary.

### 11.2.3. Rating of the severity of adverse events

Severity is a clinical determination of the intensity of an AE. The severity of an AE should be graded using the National Cancer Institute CTCEAs (National Cancer Institute (USA) 2017). For AEs that are not described in the CTCEA, the investigator will use the terminology MILD, MODERATE, or SEVERE to define the maximum severity of the adverse event as follows:

MILD: Does not interfere with the subject's normal functions; MODERATE: Interferes to some extent with the subject's normal functions; SEVERE: Significantly interferes with the subject's normal functions.

This information on the classification of AEs will be entered in the adverse events section of the CRF.

The distinction between the severity and seriousness of adverse events should be noted. A serious adverse event is not necessarily a serious event.

### 11.2.4. Evaluation of the causality of adverse events

For both serious and non-serious adverse events, the investigator should assess the potential relationship between the adverse event and the study drug, i.e., determine whether there is a reasonable possibility that the study drug caused or contributed to the adverse event. To facilitate decision-making in assessing causality, the following should be considered before making a decision:

- Medical history
- Lack of efficacy/worsening of existing disease
- Study medications
- Other medications (concomitant or prior)
- Withdrawal of study drug, especially after discontinuation of trial or after the termination of drug administration during the study
- Erroneous treatment with the study (or concomitant) medication
- Protocol-related procedures

The relationship of an AE to the investigational treatment is assessed and determined by the investigator after careful consideration of the event in terms of biological plausibility, possible unrelated causes, any pre-existing medical conditions or concomitant medication, the temporal relationship between the administration of the investigational treatment and the onset (or worsening) of the event, and known patterns of response to statins in general.

The assessment of relatedness is based on the following guidelines:

Unrelated: No temporal relationship exists with the investigational or control product or a plausible alternative explanation.

Related: All AEs are considered related if they are not judged unrelated or if there is no convincing alternative etiology.

The decision to suspend and resume treatment or permanently discontinue therapy due to an adverse event will be left to the appropriate clinical investigator.

### 11.2.5. Withdrawal from the study:

Patients will be considered to have withdrawn from the study if they have entered the study (i.e., given informed consent and received at least one dose of treatment) but have not completed the treatment phase of the study or the follow-up assessments after the EOT.

The following reasons may be considered to indicate patient withdrawal from the study: severe skin reactions or repeated moderate skin reactions; serum ALT greater than 3 X ULN at any time or serum

bilirubin elevation >2 X ULN; muscle weakness and increased CK > 1ULN; SAE or severe; any condition that the clinical investigator or any other primary care physician not involved in the study deems medically necessary to discontinue treatment and withdraw a patient from the study; significant protocol deviation; loss to follow-up; retraction of informed consent by the patient; termination of the study by the investigator. If a subject fails to return for a scheduled visit, every effort should be made to contact the subject. In all circumstances, if possible, every effort should be made to document the subject's outcome. If the subject withdraws consent, no further evaluations should be performed, and no attempt should be made to collect additional data, except safety data, which should be collected if possible. Data obtained from withdrawn patients before the withdrawal will continue to be considered. Subjects drawn from this study will not be replaced.

Discontinuation of treatment does not imply withdrawal from the study. In these cases, treatment may be interrupted for a few days; therefore, treatment will be considered incomplete or delayed. Treatment with ATO and antichagasic drugs may be resumed according to the assessment of the clinical investigator in charge of the patient. These patients should continue with study visits and evaluations as scheduled.

Subjects withdrawn from the study will not be replaced.

#### 11.2.6. Blind opening procedure

The blind opening will be performed only in the rare case of a medical emergency or unexpected, severe AEs, when the physician in charge of the patient (and not the investigator responsible for this project) considers that the patient cannot be adequately treated unless the treatment assignment is known. An appropriate blind-opening report should be raised along with completing the AE notification form provided by the ISPCH pharmacovigilance system.

Patients may receive concomitant treatment for medical incidences during the study. All concomitant treatment taken by the patient during the study, from the date of signing the informed consent until the last follow-up visit, will be recorded in the appropriate section of the CRF.

## 12.Data analysis and statistical methods:

### 12.1. Sample size:

A sample size of 75 patients per group is sufficient to achieve a power of 90% at an overall significance level of 5% (2-sided), assuming that the proportion of patients who improve electrocardiographic and echocardiographic parameters, as well as the levels of the cardiac function markers BNP and cTnT in the EOT in the experimental treatment group is 0.40. The proportion in the placebo group is 0.20, with an odds ratio of 2.5. Considering an estimated dropout rate of 25%, 100 patients per group (300 patients in total) would be recruited (Fleiss et al., 2003). The study will be sufficiently powered to provide evidence of the superior efficacy of either statin dose plus antichagasic therapy relative to antichagasic treatment alone.

### 12.2. Randomization and treatment allocation:

A computer-generated randomization list will be prepared and stratified by center using a block size of twelve. Each center will receive a list of randomization numbers and corresponding ATO or placebo packages. After patient enrollment, the following randomization number available at the

center will be assigned (in chronological order), and the corresponding treatment package will be delivered.

### 12.3. Populations to be analyzed:

Three populations will be included in the analysis: i) the intention-to-treat (ITT) population comprising all patients randomized by their assigned treatment groups, ii) the per-protocol population comprising all ITT patients without any significant deviation from protocol, and iii) the safety population comprising all patients randomized and having received at least one dose of study treatment.

Descriptive statistics for demographic data and baseline characteristics will be presented for the per-protocol and ITT populations.

### 12.4. Efficacy analysis:

For analysis of the primary outcome, a one-sided Fisher's exact test of the proportion of patients with a significant decrease in biomarker levels in the ITT (primary analysis) and per protocol (secondary analysis) populations will be used. ANOVA test will be performed to analyze differences in variances between the different markers. An exact test will be performed for all secondary comparisons of proportions between ATO versus placebo, including parasitological response to treatment. Latent class and multivariate analyses will assess the association between parasitological response, biomarker changes, and ATO doses.

### 12.5. Safety analysis:

The proportion of patients presenting at least one AE will be described. The incidence rate and 95% confidence interval per study group will be presented. Otherwise, only descriptive statistics will be presented. Laboratory safety parameters (hematology and biochemistry) will also be described individually for each study group, showing the proportion of patients by degree of elevation relative to ULN and to baseline values and changes in blood levels over time.

### 12.6. Criteria for early termination of the study

The principal investigator reserves the right to terminate the study at any time before including the planned number of subjects for valid scientific or administrative reasons. Reasons for study termination may include, but are not limited to, the following:

- Extremely low enrollment rate or lack of eligible patients
- High frequency of protocol violations
- Inaccurate or incomplete data
- Unsafe or unethical practices
- Following the recommendation of clinical ethics committees

If the study is terminated early, the clinical investigator is required to

- Complete all CRFs to the fullest extent possible and return all implements, CRFs, and related study materials provided to him/her.
- Respond to all principal investigator questions related to enrolled subjects' data at the site before study termination.
- Ensure that subjects enrolled in the study who have not yet reached a follow-up point receive necessary medical care.

- The investigator should provide written reasons for his or her decision to the national health authority and the sponsor

The investigator should provide written reasons for his or her decision to the national health authority and the ethics committee.

## 13. Ethics:
This study's experimental protocol was designed per the general ethical principles of the Declaration of Helsinki (2013).

The trial protocols (and their amendments and corrections) have been approved by the Human Research Ethics Committee of the Faculty of Medicine of the University of Chile, the Clinical Ethics Committee of the Hospital San Juan de Dios, and the Hospital Felix Bulnes and approved by the directors of the Hospitals Gustavo Fricke of Viña del Mar and San Martin de Quillota, by Law No. 20120, and its respective regulations. Each patient will give his/her informed consent, which will be recorded in the consent form

## 14. Informed consent process
Inclusion in the study will only occur if the subject gives written informed consent. The investigator is responsible for obtaining voluntary written informed consent from each individual participating in this study after adequately presenting the study objectives, methods, anticipated benefits, and potential hazards. The patient will be given time to discuss the information received with the community or family members before deciding on consent. The subject will be asked to provide written and signed consent.

If the subject is illiterate, he/she will include a checkmark on the form, and a literate witness must sign (this person should have no relationship with the research team and, if possible, should be selected by the participant).

If new safety information results in significant risk/benefit assessment changes, the consent form should be reviewed and updated if necessary. All subjects (including those already being treated) should be informed of the new information, receive a copy of the revised form, and give their consent to continue in the study.

### 14.1. Patient Costs:
Patients may be reimbursed for travel to and from the study site but will not receive any payment for participation in the trial.

## 15. Direct Access to Data and Source Documents:

The principal investigator will allow direct access to the data or source documents for monitoring, auditing, review by Ethics Committees, and inspection of the trial by health authorities.

## 16. Quality Control and Quality Assurance

The investigator should maintain adequate and accurate records that fully document the conduct of the trial and permit subsequent verification of trial data. These documents include the Investigator's Brochure, the trial protocol and its amendments, the informed consent form, the CRF, and approvals by ethics committees and health authorities. It will also contain drug accounting records, co-investigator CVs, and other appropriate documents/correspondence.

### 16.1. Case Registration Forms (CRF)

The data of each study participant will be collected by the responsible clinical researcher (RCI). Study-specific information will be entered into an electronic CRF (CRF), hosted on the RedCap platform of the Faculty of Medicine of the University of Chile, an encrypted electronic platform designed to safeguard clinical and biomedical research data.

All data entered into the CRF will be anonymous and identified exclusively by the unique patient number (NUP), assigned consecutively for each center as participants are recruited and is independent of the randomization code of the assigned treatment. This NUP is provided by the Principal Investigator (PI) of the study at the request of the ICR at each center at the time of recruiting the participant and after signing the Informed Consent Form. On the RedCap platform, the NUP will be replaced by a randomly generated code, breaking the link between the NUP and the platform. Similarly, the center and the clinical investigator will receive a unique, electronically generated code so that the patient's unique code will not be associated with the name of the clinical investigator or the study center.

The project's principal investigator will not collect personal information from study subjects in the CRF. Personal information is understood to be the name (including initials), residence address, and the commune in which the subject lives. Only the Region of origin will be recorded, nor will the contact telephone number, the exact date of birth, or any other information that would lead to the identification of the subject being recorded. Data such as family income, occupation, or educational level will not be registered, as they are not variables relevant to this study. However, the clinical investigator should maintain contact information for each participant so that all participants can be quickly contacted by the clinical investigator, if necessary.

The project clinical investigator must ensure the accuracy, completeness, legibility, and timely completion of all data reported to the PI on the CRFs and any additional information required. The PI must maintain the source documents (such as the clinical study record, laboratory, and interconsultation reports) for possible review and audit by the Scientific Ethics Committees or Regulatory Authorities. The PI is responsible for keeping all consent forms, CRFs, and the subjects' NUP list in a secure location. The documents in physical format will be kept under lock and key on the premises of the Molecular and Clinical Pharmacology program of the Faculty of Medicine of the University of Chile, with access restricted to authorized personnel only.

### 16.2. Source documents

Verification of CRF data should be performed by direct inspection of source documents. Source documents include the subject's clinical record, Clinical Investigator's notes, laboratory reports, radiology, ECG, echocardiography reports, special evaluation reports, signed informed consent forms, and subject screening and enrollment records. The records and, in general, all documentation related to the study will be stored for five years after the date of the LPO or according to the regulations in force at the time of approval of this protocol by the health authorities.

The investigator should maintain source documents (such as laboratory and consultation reports, history, and physical examination reports) for possible review and audit by Ethics Committees or Regulatory Authorities. The Clinical Investigator will record the date of each subject's visit along with a summary of the subject's status and progress in the study.

## 17. Data management and archiving of records

A CRF must be completed for all patients who have given informed consent. The present clinical trial will use an electronic CRF hosted on the RedCap platform of the Faculty of Medicine of the University of Chile, which also allows the storage of study data while maintaining confidentiality by current national legislation on patient data.

All entries in the CRF are the responsibility of the investigator or a qualified member of the designated staff. At the start of the trial, the investigator will certify in writing that his/her electronic signature is the legally binding equivalent of a written signature.

The data will be continuously reviewed by a designated clinical monitor for this purpose. Data queries will be generated, documented, and resolved on an ongoing basis during the trial.

Investigators must ensure subjects' anonymity, and their identities are protected from unauthorized parties. In CRFs or other documents, subjects should not be identified by their names but exclusively by an identification code. The principal investigator should maintain a subject enrollment list showing codes, names, and addresses. The principal investigator should maintain strict confidentiality of documents submitted for audit and written consent forms signed by the subject.

## 18. Reports and publications

This clinical trial will be registered in a recognized clinical trial registry, such as www.clinicaltrials.gov.

The results of this study may be published or presented at scientific meetings.

By standard editorial and ethical practice, publication of multicenter trials only in their entirety and not as individual center data.

## 19. References

Acevedo, G.R., M.C. Girard, and K.A. Gomez. 2018. The Unsolved Jigsaw Puzzle of the Immune Response in Chagas Disease. Front Immunol. 9:1929.

Alonso-Padilla, J., M. Gallego, A.G. Schijman, and J. Gascon. 2017. Molecular diagnostics for Chagas disease: up to date and novel methodologies. Expert Rev Mol Diagn. 17:699-710.

Andrade JP, Marin-Neto JA, Paola AA, Vilas-Boas F, Oliveira GM, Bacal F, Bocchi EA, Almeida DR, Fragata Filho AA, Moreira M da C, Xavier SS, Oliveira Junior WA, Dias JC. 2011. Sociedade Brasileira de Cardiologia. I Diretriz Latino Americana para o Diagnóstico e Tratamento da Cardiopatia Chagásica. Arq bras Cardiol. 97(2 Suppl 3):1-48.

Apt, W., X. Aguilera, A. Arribada, C. Perez, C. Miranda, G. Sanchez, I. Zulantay, P. Cortes, J. Rodriguez, and D. Juri. 1998. Treatment of chronic Chagas' disease with itraconazole and allopurinol. Am J Trop Med Hyg. 59:133-138.

Apt, W., A. Arribada, I. Zulantay, J. Rodriguez, M. Saavedra, and A. Munoz. 2013. Treatment of Chagas' disease with itraconazole: electrocardiographic and parasitological conditions after 20 years of follow-up. J Antimicrob Chemother. 68:2164-2169.

Barizon, G.C., M.V. Simoes, A. Schmidt, L.P. Gadioli, and L.O. Murta Junior. 2020. Relationship between microvascular changes, autonomic denervation, and myocardial fibrosis in Chagas cardiomyopathy: Evaluation by MRI and SPECT imaging. J Nucl Cardiol. 27:434-444.

Bern, C. 2015. Chagas' Disease. N Engl J Med. 373:456-466.

Bestetti, R.B., and C.B. Restini. 2014. Precordial chest pain in patients with chronic Chagas disease. Int J Cardiol. 176:309-314.

Booney, K.M., D.J. Luthringer, S.A. Kim, N.J. Garg, and D.M. Engman. 2019. Pathology and Pathogenesis of Chagas Disease Heart. Annu. Rev. Pathol. Mech. Dis. 14:421-447.

Borges, J.P., F. Mendes, G.O. Lopes, A.S. Sousa, M.F.F. Mediano, and E. Tibirica. 2018. Is endothelial microvascular function equally impaired among patients with chronic Chagas and ischemic cardiomyopathy? Int J Cardiol. 265:35-37.

Botoni, F.A., P.A. Poole-Wilson, A.L. Ribeiro, D.O. Okonko, B.M. Oliveira, A.S. Pinto, M.M. Teixeira, A.L. Teixeira, Jr., A.M. Reis, J.B. Dantas, C.S. Ferreira, W.C. Tavares, Jr., and M.O. Rocha. 2007. A randomized trial of carvedilol after renin-angiotensin system inhibition in chronic Chagas cardiomyopathy. Am Heart J. 153:544 e541-548.

Brener, Z., J.R. Cancado, L.M. Galvao, Z.M. da Luz, S. Filardi Lde, M.E. Pereira, L.M. Santos, and C.B. Cancado. 1993. An experimental and clinical assay with ketoconazole in the treatment of Chagas disease. Mem Inst Oswaldo Cruz. 88:149-153.

Brown BG, Zhao XQ, Chait A, Fisher LD, Cheung MC, Morse JS, Dowdy AA, Marino EK, Bolson EL, Alaupovic P, Frohlich J, Albers JJ. Simvastatin and niacin, antioxidant vitamins, or the combination for the prevention of coronary disease. N Engl J Med. 2001 Nov 29;345(22):1583-92

Campos-Estrada, C., A. Liempi, F. Gonzalez-Herrera, M. Lapier, U. Kemmerling, B. Pesce, J. Ferreira, R. Lopez-Munoz, and J.D. Maya. 2015. Simvastatin and Benznidazole-Mediated Prevention of Trypanosoma cruzi-Induced Endothelial Activation: Role of 15-epi-lipoxin A4 in the Action of Simvastatin. PLoS Negl Trop Dis. 9:e0003770.

Cardillo, F., J.C. Voltarelli, S.G. Reed, and J.S. Silva. 1996. Regulation of Trypanosoma cruzi infection in mice by gamma interferon and interleukin 10: role of NK cells. Infect Immun. 64:128-134.

Cortes-Serra, N., I. Losada-Galvan, M.J. Pinazo, C. Fernandez-Becerra, J. Gascon, and J. Alonso-Padilla. 2020. State-of-the-art in host-derived biomarkers of Chagas disease prognosis and early evaluation of anti-Trypanosoma cruzi treatment response. Biochim Biophys Acta Mol Basis Dis. 1866:165758.

Crespillo-Andujar, C., S. Chamorro-Tojeiro, F. Norman, B. Monge-Maillo, R. Lopez-Velez, and J.A. Perez-Molina. 2018a. Toxicity of nifurtimox as second-line treatment after benznidazole intolerance in patients with chronic Chagas disease: when available options fail. Clin Microbiol Infect. 24:1344 e1341-1344 e1344.

Crespillo-Andujar, C., E. Venanzi-Rullo, R. Lopez-Velez, B. Monge-Maillo, F. Norman, A. Lopez-Polin, and J.A. Perez-Molina. 2018b. Safety Profile of Benznidazole in the Treatment of Chronic Chagas Disease: Experience of a Referral Centre and Systematic Literature Review with Meta-Analysis. Drug Saf. 41:1035-1048.

Duffy, T., C.I. Cura, J.C. Ramirez, T. Abate, N.M. Cayo, R. Parrado, Z.D. Bello, E. Velazquez, A. Munoz-Calderon, N.A. Juiz, J. Basile, L. Garcia, A. Riarte, J.R. Nasser, S.B. Ocampo, Z.E. Yadon, F. Torrico, B.A. de Noya, I. Ribeiro, and A.G. Schijman. 2013. Analytical performance of a multiplex Real-Time PCR assay using TaqMan probes for quantification of Trypanosoma cruzi satellite DNA in blood samples. PLoS Negl Trop Dis. 7:e2000.

Dzikowska-Diduch, O., J. Domienik-Karlowicz, E. Gorska, U. Demkow, P. Pruszczyk, and M. Kostrubiec. 2017. E-selectin and sICAM-1, biomarkers of endothelial function, predict recurrence of venous thromboembolism. Thromb Res. 157:173-180.

Echeverria, L.E., L.Z. Rojas, M.C. Villamizar, C. Luengas, A.M. Chaves, J.A. Rodriguez, R. Campo, C. Clavijo, A.M. Redondo, L.A. Lopez, S.A. Gomez-Ochoa, C.A. Morillo, O.L. Rueda-Ochoa, and O.H. Franco. 2020. Echocardiographic parameters, speckle tracking, and brain natriuretic peptide levels as indicators of progression of indeterminate stage to Chagas cardiomyopathy. Echocardiography. 37:429-438.

Fleiss, J.L., B. Levin, and M.C. Paik. 2003. Statistical Methods for Rates and Proportions. Willey, New York, USA. 800 pp.

Gonzalez-Herrera, F., A. Cramer, P. Pimentel, C. Castillo, A. Liempi, U. Kemmerling, F.S. Machado, and J.D. Maya. 2017. Simvastatin Attenuates Endothelial Activation through 15-Epi-Lipoxin A4 Production in Murine Chronic Chagas Cardiomyopathy. Antimicrob Agents Chemother. 61.

Guzman-Rivera, D., A. Liempi, F. Gonzalez-Herrera, S. Fuentes, I. Carrillo, P. Abarca, C. Castillo, U. Kemmerling, B. Pesce, and J.D. Maya. 2020. Simvastatin improves cardiac function through Notch1 activation in BALB/c mice with chronic Chagas cardiomyopathy. Antimicrob Agents Chemother.

Hasegawa, S., T. Miura, S. Sasaki, H. Madarame, and A. Nakane. 2002. Dysregulation of interleukin-10 and interleukin-12 are involved in the reduced host resistance to Listeria monocytogenes infection in alymphoplastic aly mutant mice. FEMS Immunol Med Microbiol. 32:111-117.

Hernandez, M., S. Wicz, M.H. Santamaria, and R.S. Corral. 2018. Curcumin exerts anti-inflammatory and vasoprotective effects through amelioration of NFAT-dependent endothelin-1 production in mice with acute Chagas cardiomyopathy. Mem Inst Oswaldo Cruz. 113:e180171.

Hiss, F.C., T.F. Lascala, B.C. Maciel, J.A. Marin-Neto, and M.V. Simoes. 2009. Changes in myocardial perfusion correlate with deterioration of left ventricular systolic function in chronic Chagas' cardiomyopathy. JACC Cardiovasc Imaging. 2:164-172.

Jackson, Y., B. Wyssa, and F. Chappuis. 2020. Tolerance to nifurtimox and benznidazole in adult patients with chronic Chagas' disease. J Antimicrob Chemother. 75:690-696.

Jefferies, J.L., and J.A. Towbin. 2010. Dilated cardiomyopathy. Lancet. 375:752-762.

Jenkins DJA, Spence JD, Giovannucci EL, Kim YI, Josse RG, Vieth R, Sahye-Pudaruth S, Paquette M, Patel D, Blanco Mejia S, Viguiliouk E, Nishi SK, Kavanagh M, Tsirakis T, Kendall CWC, Pichika SC, Sievenpiper JL. 2021. Supplemental Vitamins and Minerals for Cardiovascular Disease Prevention and Treatment: JACC Focus Seminar. J Am Coll Cardiol. Feb 2;77(4):423-436

Jercic, M.I., and A. Oyarce. 2019. Recomendaciones técnicas para la selección de método para el tamizaje serológico de la enfermedad de Chagas. D.B.N.y.d.R.I.d.S.P.d.C. Sección Parasitología, editor. Instituto de Salud Publica, Ministerio de Salud, Gobierno de Chile.

Lee, B.Y., K.M. Bacon, M.E. Bottazzi, and P.J. Hotez. 2013. Global economic burden of Chagas disease: a computational simulation model. Lancet Infect Dis. 13:342-348.

Lemos de Oliveira, L.F., J.T. Thackeray, J.A. Marin Neto, M.M. Dias Romano, E.E. Vieira de Carvalho, J. Mejia, D.M. Tanaka, G. Kelly da Silva, D.R. Abdalla, C. Malamut, F.M. Bengel, M. de Lourdes Higuchi, A. Schmidt, E. Cunha-Neto, and M.V. Simoes. 2018. Regional Myocardial Perfusion Disturbance in Experimental Chronic Chagas Cardiomyopathy. J Nucl Med. 59:1430-1436.

Lenk, E.J., W.K. Redekop, M. Luyendijk, C. Fitzpatrick, L. Niessen, W.A. Stolk, F. Tediosi, A.J. Rijnsburger, R. Bakker, J.A.C. Hontelez, J.H. Richardus, J. Jacobson, E.A. Le Rutte, S.J. de Vlas, and J.L. Severens. 2018. Socioeconomic benefit to individuals of achieving 2020 targets for four neglected tropical diseases controlled/eliminated by innovative and intensified disease management: Human African trypanosomiasis, leprosy, visceral leishmaniasis, Chagas disease. PLoS Negl Trop Dis. 12:e0006250.

Llaguno, M., M.V. da Silva, L.R. Batista, D.A.A. da Silva, R.C. de Sousa, L. de Resende, V.J.D. da Silva, E. Lages-Silva, C.J.F. Oliveira, J.R. Machado, D.B.R. Rodrigues, D. Correia, and V. Rodrigues. 2019. T-Cell Immunophenotyping and Cytokine Production Analysis in Patients with Chagas Disease 4 Years after Benznidazole Treatment. Infect Immun. 87.

Lopez-Munoz, R., M. Faundez, S. Klein, S. Escanilla, G. Torres, D. Lee-Liu, J. Ferreira, U. Kemmerling, M. Orellana, A. Morello, A. Ferreira, and J.D. Maya. 2010. Trypanosoma cruzi: In vitro effect of aspirin with nifurtimox and benznidazole. Exp Parasitol. 124:167-171.

Marin-Neto, J.A., M.V. Simoes, and A. Rassi Junior. 2013. Pathogenesis of chronic Chagas cardiomyopathy: the role of coronary microvascular derangements. Rev Soc Bras Med Trop. 46:536-541.

Marti-Carvajal, A.J., and J.S. Kwong. 2016. Pharmacological interventions for treating heart failure in patients with Chagas cardiomyopathy. Cochrane Database Syst Rev. 7:CD009077.

MINSAL. 2017. Manual de procedimiento para la atención de pacientes con enfermedad de Chagas. D.d.P.y.C.E.M.d.S. Departamento de Enfermedades Transmisibles, editor. Ministerio de Salud Publica, Gobierno de Chile.

Molina-Berrios, A., C. Campos-Estrada, N. Henriquez, M. Faundez, G. Torres, C. Castillo, S. Escanilla, U. Kemmerling, A. Morello, R.A. Lopez-Munoz, and J.D. Maya. 2013a. Protective role of acetylsalicylic acid in experimental Trypanosoma cruzi infection: evidence of a 15-epi-lipoxin A(4)-mediated effect. PLoS Negl Trop Dis. 7:e2173.

Molina-Berrios, A., C. Campos-Estrada, M. Lapier, J. Duaso, U. Kemmerling, N. Galanti, J. Ferreira, A. Morello, R. Lopez-Munoz, and J.D. Maya. 2013b. Protection of vascular endothelium by aspirin in a murine model of chronic Chagas' disease. Parasitol Res. 112:2731-2739.

Molina-Berrios, A., C. Campos-Estrada, M. Lapier, J. Duaso, U. Kemmerling, N. Galanti, M. Leiva, J. Ferreira, R. Lopez-Munoz, and J.D. Maya. 2013c. Benznidazole prevents endothelial damage in an experimental model of Chagas disease. Acta Trop. 127:6-13.

Molina, I., J. Gomez i Prat, F. Salvador, B. Trevino, E. Sulleiro, N. Serre, D. Pou, S. Roure, J. Cabezos, L. Valerio, A. Blanco-Grau, A. Sanchez-Montalva, X. Vidal, and A. Pahissa. 2014. Randomized trial of posaconazole and benznidazole for chronic Chagas' disease. N Engl J Med. 370:1899-1908.

Molina, I., F. Salvador, A. Sanchez-Montalva, M.A. Artaza, R. Moreno, L. Perin, A. Esquisabel, L. Pinto, and J.L. Pedraz. 2017. Pharmacokinetics of Benznidazole in Healthy Volunteers and Implications in Future Clinical Trials. Antimicrob Agents Chemother. 61.

Morillo, C.A., J.A. Marin-Neto, A. Avezum, S. Sosa-Estani, A. Rassi, Jr., F. Rosas, E. Villena, R. Quiroz, R. Bonilla, C. Britto, F. Guhl, E. Velazquez, L. Bonilla, B. Meeks, P. Rao-Melacini, J. Pogue, A. Mattos, J. Lazdins, A. Rassi, S.J. Connolly, S. Yusuf, and B. Investigators. 2015. Randomized Trial of Benznidazole for Chronic Chagas' Cardiomyopathy. N Engl J Med. 373:1295-1306.

Moroni, S., M.E. Marson, G. Moscatelli, G. Mastrantonio, M. Bisio, N. Gonzalez, G. Ballering, J. Altcheh, and F. Garcia-Bournissen. 2019. Negligible exposure to nifurtimox through breast milk during maternal treatment for Chagas Disease. PLoS Negl Trop Dis. 13:e0007647.

Nunes, M.C.P., A. Beaton, H. Acquatella, C. Bern, A.F. Bolger, L.E. Echeverria, W.O. Dutra, J. Gascon, C.A. Morillo, J. Oliveira-Filho, A.L.P. Ribeiro, J.A. Marin-Neto, E. American Heart Association Rheumatic Fever, Y. Kawasaki Disease Committee of the Council on Cardiovascular Disease in the, C. Council on, N. Stroke, and C. Stroke. 2018. Chagas Cardiomyopathy: An Update of Current Clinical Knowledge and Management: A Scientific Statement From the American Heart Association. Circulation. 138:e169-e209.

OPS. 2018. Guía para el diagnóstico y el tratamiento de la enfermedad de Chagas. Organización Panamericana de la Salud, Washington D.C. U.S.A.

Parrado, R., J.C. Ramirez, A. de la Barra, C. Alonso-Vega, N. Juiz, L. Ortiz, D. Illanes, F. Torrico, J. Gascon, F. Alves, L. Flevaud, L. Garcia, A.G. Schijman, and I. Ribeiro. 2019. Usefulness of Serial Blood Sampling and PCR Replicates for Treatment Monitoring of Patients with Chronic Chagas Disease. Antimicrob Agents Chemother. 63.

Pengue, C., G. Cesar, M.G. Alvarez, G. Bertocchi, B. Lococo, R. Viotti, M.A. Natale, M.D. Castro Eiro, S.S. Cambiazzo, N. Perroni, M. Nunez, M.C. Albareda, and S.A. Laucella. 2019. Impaired frequencies and function of platelets and tissue remodeling in chronic Chagas disease. PLoS One. 14:e0218260.

Perez-Anton, E., A. Egui, M.C. Thomas, M. Simon, M. Segovia, and M.C. Lopez. 2020. Immunological exhaustion and functional profile of CD8(+) T lymphocytes as cellular biomarkers of therapeutic efficacy in chronic Chagas disease patients. Acta Trop. 202:105242.

Rassi, A., J.M. de Rezende, A.O. Luquetti, and A. Rassi. 2017a. 28 - Clinical phases and forms of Chagas disease. In American Trypanosomiasis Chagas Disease (Second Edition). J. Telleria and M. Tibayrenc, editors. Elsevier, London. 653-686.

Rassi, A., Jr., J.A.N. Marin, and A. Rassi. 2017b. Chronic Chagas cardiomyopathy: a review of the main pathogenic mechanisms and the efficacy of aetiological treatment following the BENznidazole Evaluation for Interrupting Trypanosomiasis (BENEFIT) trial. Mem Inst Oswaldo Cruz. 112:224-235.

Rassi, A., Jr., A. Rassi, W.C. Little, S.S. Xavier, S.G. Rassi, A.G. Rassi, G.G. Rassi, A. Hasslocher-Moreno, A.S. Sousa, and M.I. Scanavacca. 2006. Development and validation of a risk score for predicting death in Chagas' heart disease. N Engl J Med. 355:799-808.

Rassi, A., Jr., A. Rassi, and J.A. Marin-Neto. 2010. Chagas disease. Lancet. 375:1388-1402.

Rassi, A.J.R., A; Marin-Neto 2010. Chagas disease. Lancet. 375:1388-1402.

Requena-Mendez, A., S. Bussion, E. Aldasoro, Y. Jackson, A. Angheben, D. Moore, M.J. Pinazo, J. Gascon, J. Munoz, and E. Sicuri. 2017. Cost-effectiveness of Chagas disease screening in Latin

American migrants at primary health-care centres in Europe: a Markov model analysis. Lancet Glob Health. 5:e439-e447.

Ribeiro, V., N. Dias, T. Paiva, L. Hagstrom-Bex, N. Nitz, R. Pratesi, and M. Hecht. 2020. Current trends in the pharmacological management of Chagas disease. Int J Parasitol Drugs Drug Resist. 12:7-17.

Rojas, L.Z., M. Glisic, L. Pletsch-Borba, L.E. Echeverria, W.M. Bramer, A. Bano, N. Stringa, A. Zaciragic, B. Kraja, E. Asllanaj, R. Chowdhury, C.A. Morillo, O.L. Rueda-Ochoa, O.H. Franco, and T. Muka. 2018. Electrocardiographic abnormalities in Chagas disease in the general population: A systematic review and meta-analysis. PLoS Negl Trop Dis. 12:e0006567.

Rossi, M.A., H.B. Tanowitz, L.M. Malvestio, M.R. Celes, E.C. Campos, V. Blefari, and C.M. Prado. 2010. Coronary microvascular disease in chronic Chagas cardiomyopathy including an overview on history, pathology, and other proposed pathogenic mechanisms. PLoS Negl Trop Dis. 4.

Sales Junior, P.A., I. Molina, S.M. Fonseca Murta, A. Sanchez-Montalva, F. Salvador, R. Correa-Oliveira, and C.M. Carneiro. 2017. Experimental and Clinical Treatment of Chagas Disease: A Review. Am J Trop Med Hyg. 97:1289-1303.

Schijman, A.G., J. Altcheh, J.M. Burgos, M. Biancardi, M. Bisio, M.J. Levin, and H. Freilij. 2003. Aetiological treatment of congenital Chagas' disease diagnosed and monitored by the polymerase chain reaction. J Antimicrob Chemother. 52:441-449.

Schmunis, G. 2013. Status of and cost of Chagas disease worldwide. Lancet Infect Dis. 13:283-284.

Serhan, C.N. 2017. Discovery of specialized pro-resolving mediators marks the dawn of resolution physiology and pharmacology. Mol Aspects Med. 58:1-11.

Serhan, C.N., and N. Chiang. 2013. Resolution phase lipid mediators of inflammation: agonists of resolution. Curr Opin Pharmacol. 13:632-640.

Sguassero, Y., K.N. Roberts, G.B. Harvey, D. Comande, A. Ciapponi, C.B. Cuesta, C. Aguiar, A.M. Castro, E. Danesi, A.L. de Andrade, M. de Lana, J.M. Escriba, D.L. Fabbro, C.D. Fernandes, M. Flores-Chavez, A.M. Hasslocher-Moreno, Y. Jackson, C.D. Lacunza, G.F. Machado-de-Assis, M. Maldonado, W.S.F. Meira, I. Molina, M.M. Monje-Rumi, C. Munoz-San Martin, L. Murcia, C. Nery de Castro, O. Sanchez Negrette, M. Segovia, C.A.N. Silveira, A. Solari, M. Steindel, M.L. Streiger, N. Vera de Bilbao, I. Zulantay, and S. Sosa-Estani. 2018. Course of serological tests in treated subjects with chronic Trypanosoma cruzi infection: A systematic review and meta-analysis of individual participant data. Int J Infect Dis. 73:93-101.

Sousa, G.R., J.A. Gomes, M.P. Damasio, M.C. Nunes, H.S. Costa, N.I. Medeiros, R.C. Fares, A.T. Chaves, R. Correa-Oliveira, and M.O. Rocha. 2017. The role of interleukin 17-mediated immune response in Chagas disease: High level is correlated with better left ventricular function. PLoS One. 12:e0172833.

Souza-Silva, T.G., L.F. Diniz, A. Lia Mazzeti, A.A.S. Mendonca, R.V. Goncalves, and R.D. Novaes. 2019. Could angiotensin-modulating drugs be relevant for the treatment of Trypanosoma cruzi infection? A systematic review of preclinical and clinical evidence. Parasitology. 146:914-927.

Spite, M., and C.N. Serhan. 2010. Novel lipid mediators promote resolution of acute inflammation: impact of aspirin and statins. Circ Res. 107:1170-1184.

Stein JH, Carlsson CM, Papcke-Benson K, Aeschlimann SE, Bodemer A, Carnes M, McBride PE. The effects of lipid-lowering and antioxidant vitamin therapies on flow-mediated vasodilation of the brachial artery in older adults with hypercholesterolemia. J Am Coll Cardiol. 2001 Dec;38(7):1806-13.

Stein, C., C.B. Migliavaca, V. Colpani, P.R. da Rosa, D. Sganzerla, N.E. Giordani, S. Miguel, L.N. Cruz, C.A. Polanczyk, A.L.P. Ribeiro, and M. Falavigna. 2018. Amiodarone for arrhythmia in patients with Chagas disease: A systematic review and individual patient data meta-analysis. PLoS Negl Trop Dis. 12:e0006742.

Stone PH, Lloyd-Jones DM, Kinlay S, Frei B, Carlson W, Rubenstein J, Andrews TC, Johnstone M, Sopko G, Cole H, Orav J, Selwyn AP, Creager MA; Vascular Basis Study Group. Effect of intensive lipid lowering, with or without antioxidant vitamins, compared with moderate lipid lowering on myocardial ischemia in patients with stable coronary artery disease: the Vascular Basis for the Treatment of Myocardial Ischemia Study. Circulation. 2005 Apr 12;111(14):1747-55

Tanaka, D.M., L.F.L. de Oliveira, J.A. Marin-Neto, M.M.D. Romano, E.E.V. de Carvalho, A.C.L. de Barros Filho, F.F.F. Ribeiro, J.M. Cabeza, C.D. Lopes, C.G. Fabricio, N. Kesper, H.T. Moreira, L. Wichert-Ana, A. Schmidt, M.L. Higuchi, E. Cunha-Neto, and M.V. Simoes. 2019. Prolonged dipyridamole administration reduces myocardial perfusion defects in experimental chronic Chagas cardiomyopathy. J Nucl Cardiol. 26:1569-1579.

Torrico, F., J. Gascon, L. Ortiz, C. Alonso-Vega, M.J. Pinazo, A. Schijman, I.C. Almeida, F. Alves, N. Strub-Wourgaft, I. Ribeiro, and E.S. Group. 2018. Treatment of adult chronic indeterminate Chagas disease with benznidazole and three E1224 dosing regimens: a proof-of-concept, randomised, placebo-controlled trial. Lancet Infect Dis. 18:419-430.

Varela, M.T., and J.P.S. Fernandes. 2020. Natural Products: Key Prototypes to Drug Discovery Against Neglected Diseases Caused by Trypanosomatids. Curr Med Chem. 27:2133-2146.

WHO. 2011. Causes of Death in 2008. In Mortality and global health estimates. Vol. 2013. World Health Organization, Global Health Observatory Data Repository.

WHO. 2015. Investing to overcome the global impact of neglected tropical diseases: third WHO report on neglected tropical diseases. World Health Organization, Geneva. 191 p. pp.

WHO. 2018. Global Health Estimates 2016: Disease burden by Cause, Age, Sex, by Country and by Region, 2000-2016, Geneva.

Zabihi M, Askarian F, Hekmatimoghaddam S, Rashidi Nooshabadi M, Zabihi MS, Mousavinasab SR. Ascorbic Acid Significantly Decreases Creatine Kinase Plasma Levels in an Animal Model of Statin/Fibrate-Induced Myopathy. Adv Pharmacol Pharm Sci. 2021 Dec 29;2021:5539595
