# Supplementary material for: Effect of statins on inflammation and cardiac function in patients with chronic Chagas disease: A protocol for pathophysiological studies in a multicenter, placebo-controlled, proof-of-concept phase II trial
Source: PLoS One. 2023 Jan 13;18(1):e0280335. doi: 10.1371/journal.pone.0280335 (PMC9838836; doi:10.1371/journal.pone.0280335)
Supplement: S3 File — (PDF) [file pone.0280335.s003.pdf]

PROTOCOLO ENSAYO CLINICO

1. **Código del proyecto:** FONDECYT 1210359
2. **Patrocinador/Financiador**: FONDECYT grant N° 1210359
3. **Título del estudio Clínico:**

Efecto de las estatinas sobre la inflamación y la función cardíaca en pacientes con enfermedad de Chagas crónica: Estudios fisiopatológicos en un ensayo clínico multicéntrico de prueba de concepto

4. **Equipo de investigación:**

Investigador Principal:

Dr. Juan Diego Maya Arango. Facultad de Medicina, Universidad de Chile. Dirección de la Institución: Avenida Independencia 1027, Independencia, Santiago. Número de teléfono de la Institución: 56 2 2978 6071; correo electrónico: jdmaya@uchile.cl.

Coinvestigadoras:

Marisol del Carmen Denegri Cartes: Departamento de Pediatría Occidente, Facultad de Medicina, Universidad de Chile; Hospital Félix Bulnes, Cerro Navia. Correo Electrónico: marisoldenegri@hotmail.com

Miren Edurne Urarte Izeta. Departamento de Pediatría Occidente, Facultad de Medicina, Universidad de Chile; Hospital San Juan De Dios, Santiago. Correo Electrónico: eurarte@med.uchile.cl

Litzi Villalón Quezada. Hospital Gustavo Fricke y Hospital San Martin de Quillota; correo electrónico: litzi.villalon@redsalud.gob.cl

Ulrike Kemmerling Weis: Facultad de Medicina, universidad de Chile. Correo electrónico: ukemmerling@uchile.cl

Carolina Campos Estrada: Facultad de Farmacia, Universidad de Valparaíso. Correo electrónico: carolina.campos@uv.cl

5. **Fase del Estudio:** Estudio Fase II de prueba de concepto

6. RESUMEN

TITULO DEL ESTUDIO CLÍNICO

Efecto de las estatinas sobre la inflamación y la función cardíaca en pacientes con enfermedad de Chagas crónica: Estudios fisiopatológicos en un ensayo clínico multicéntrico de prueba de concepto

TITULO ABREVIADO: Estudio multicéntrico de fase 2 de Estatinas en enfermedad de Chagas crónica

FASE: Fase II – Prueba de Concepto

OBJETIVOS:

- **OBJETIVO PRIMARIO:**
- Evaluar si el uso de la atorvastatina en combinación con la terapia antiparasitaria (NFX o BZD), es segura y más eficaz que la terapia antiparasitaria sola en prevenir la aparición de alteraciones cardiacas mediante la reducción de la inflamación general y en la mejora de las funciones endotelial y cardíaca
- **OBJETIVOS SECUNDARIOS**
- Evaluar la eficacia de la combinación de ATO y terapia antichagásica para disminuir:
    - La inflamación, medida por los niveles plasmáticos de las citocinas, TNF-α, IFN-γ, IL-10, IL-1B, IL-4 e IL-17A.
    - La activación endotelial, medida por los niveles plasmáticos de sCAM: sE-selectina, sICAM-1 y sVCAM-1.
    - El daño y la función cardíaca: medidos por los niveles plasmáticos de BNP, y cTnT, el ECG de 12 derivaciones en reposo, y el ecocardiograma 2D.
- Determinar la seguridad y tolerabilidad de la combinación de ATO con la terapia antichagásica, medida por la incidencia de EA (por ejemplo, rabdomiólisis) y la interrupción de la terapia.
- Evaluar la respuesta al tratamiento de la combinación de ATO con la terapia antichagásica, medida por la PCR cuantitativa y la serología, durante un período de seguimiento de diez meses.
- Determinar el grado de cumplimiento de las terapias mediante la medición de la responsabilidad de los medicamentos a lo largo del estudio.

DISEÑO DEL ESTUDIO: Se trata de un ensayo clínico de fase II de prueba de concepto con tres grupos diferentes: dos grupos recibirán terapia antichagásica más 40 o 80 mg/día de ATO, respectivamente. El tercer grupo recibirá placebo más terapia antichagásica. Los pacientes recibirán primero la terapia antichagásica convencional durante dos meses y luego de un período de dos semanas (o hasta normalización de enzimas hepáticas) se iniciará el tratamiento con atorvastatina o placebo durante cuatro meses. Estas dosis se eligieron para minimizar los resultados falsos negativos, proporcionando así la mejor prueba de la hipótesis y maximizando el efecto farmacodinámico sobre la inflamación y la función cardíaca y endotelial. Además, la secuencia de los tratamientos está dirigida a minimizar las reacciones adversas que se podría originar por la eventual combinación de los fármacos antichagásicos con atorvastatina. Los sujetos del estudio serán aleatorizados, y los tres grupos serán doble ciego (tanto los investigadores clínicos como los sujetos serán ciegos) para ATO o placebo. Sin embargo, la terapia antichagásica será abierta. Además, el qPCR y las otras evaluaciones de laboratorio, así como los ECG y

ecocardiogramas 2D se realizarán con los investigadores clínicos ciegos a la asignación de ATO o placebo.

Como se mencionó anteriormente, este estudio se llevará a cabo en cuatro centros: 1) Hospital San Juan de Dios y 2) Hospital Félix Bulnes en Santiago, Región Metropolitana, 3) Hospital Dr. Gustavo Fricke de Viña del Mar, y 4) Hospital San Martín de Quillota, Región de Valparaíso.

ABSTRACTO: Las complicaciones cardíacas, incluyendo la insuficiencia cardíaca y las arritmias, son las principales causas de discapacidad y muerte en la enfermedad de Chagas (EC). Postulamos que las estatinas mejoran la inflamación vascular y cardíaca en pacientes con EC crónica, contribuyendo a la recuperación de la función endotelial y cardíaca y, por tanto, mejorando la eficacia del tratamiento antichagásico.

La EC, causada por el parásito protozoario *Trypanosoma cruzi*, afecta a 7 millones de personas en América Latina, y su incidencia está aumentando en los países no endémicos debido a la migración. Es la segunda enfermedad con mayor carga de morbilidad entre las enfermedades tropicales, y su coste económico global en el mundo supera los 7.000 millones de dólares anuales. El diagnóstico radica en la sospecha clínica y la detección serológica de anticuerpos. La evaluación cardíaca es esencial para determinar el estado funcional del corazón y el riesgo de mortalidad. El compromiso cardiaco se explica por una lesión miocárdica inmunomediada dependiente del parásito y por anomalías microvasculares e isquemia. Maya y colaboradores demostraron que un rasgo distintivo del daño microvascular inducido por *T. cruzi* es el aumento de las moléculas de adhesión endotelial vascular, un efecto contrarrestado por el fármaco reductor del colesterol simvastatina.

El tratamiento actual de la EC temprana incluye la administración de nifurtimox y benznidazol, mientras que en la fase crónica su eficacia es baja y puede inducir eventos adversos graves, lo que obliga a suspender la terapia. Por lo tanto, es de suma importancia encontrar enfoques innovadores para tratar esta enfermedad potencialmente mortal. Así, la mejora de la eficacia de los actuales fármacos antichagásicos mediante la modificación de la respuesta inflamatoria haría más eficaz el tratamiento actual.

En los estudios preclínicos más recientes con ratones infectados crónicamente, la simvastatina demostró (i) disminuir la inflamación cardíaca, (ii) reducir la activación endotelial y (iii) mejorar la función cardíaca; efectos que requieren confirmación clínica. Así, nuestra propuesta tiene como objetivo analizar si una estatina, a través de los cambios mencionados, podría mejorar la terapia antichagásica en el contexto de la EC crónica a través de un ensayo clínico de fase II. Por ello, proponemos la coadministración de atorvastatina (ATO) como estatinas experimentales. Sus perfiles terapéuticos y de seguridad son bien conocidos, así como su mecanismo de acción, que comparten los demás miembros de la clase de las estatinas. Debido a la baja incidencia de efectos adversos graves y a su eficacia, ambas son las estatinas más utilizadas en la actualidad.

Esta propuesta tiene como objetivo evaluar si el tratamiento con 40 u 80 mg de ATO, en combinación con la terapia antiparasitaria, es seguro y más eficaz en la reducción de la inflamación general que una terapia antiparasitaria sola, lo que permite a los investigadores probar la hipótesis de que el efecto antiinflamatorio de las estatinas antiinflamatoria mejora las funciones endotelial y cardíaca.

Este ensayo de prueba de concepto será doble ciego, aleatorio y multicéntrico, con un diseño de fase II.

Para lograr este objetivo, evaluaremos la eficacia de la combinación de ATO y la terapia antichagásica para reducir los niveles plasmáticos de citoquinas inflamatorias, las moléculas solubles de adhesión celular endotelial, y confirmaremos la mejora de la función cardíaca mediante electrocardiograma y ecocardiograma bidimensional.

El estudio establecerá la seguridad y tolerabilidad de la combinación de atorvastatina con la terapia

antichagásica mediante el seguimiento de la incidencia de eventos adversos y la interrupción de la terapia. Este estudio se realizará con un tamaño de muestra de 300 pacientes adultos en cuatro centros del programa nacional de Chagas: 1) Hospital San Juan de Dios y 2) Hospital Félix Bulnes en Santiago, Región Metropolitana, y 3) Hospital Dr. Gustavo Fricke en Viña del Mar y 4) Hospital San Martín de Quillota, Región de Valparaíso, coordinado por las doctoras Edurne Urarte, Marisol Denegri y Litzi Villalón, respectivamente.

Los adultos con EC crónica son la población que requiere con mayor urgencia nuevos tratamientos, ya que la mayor carga de la enfermedad se encuentra en estos pacientes. Así, la mejora de los factores desencadenantes del hospedero, como la inflamación, la activación endotelial y la función cardíaca, con la adición de ATO, puede aumentar la eficacia de la terapia antichagásica convencional.

Dada la escasa evidencia sobre el tratamiento de esta enfermedad, este estudio ofrecerá la oportunidad de evaluar una novedosa estrategia terapéutica combinada sobre prometedores marcadores candidatos de inflamación, actividad endotelial y función cardíaca, y de correlacionar los resultados con los resultados parasitológicos. Este ensayo podría ser el primer paso hacia la evaluación de una nueva terapia potencial propuesta para modificar favorablemente el curso de la enfermedad de Chagas crónica.

NUMERO DE CENTROS DEL ESTUDIO:

Cuatro centros:

1) Hospital San Juan de Dios y 2) Hospital Félix Bulnes en Santiago, Región Metropolitana, y

3) Hospital Dr. Gustavo Fricke en Viña del Mar y 4) Hospital San Martín de Quillota, Región de Valparaíso.

Estos cuatro centros hacen parte de la red de atención del Programa Nacional de Control de la Enfermedad de Chagas.

Duración de la participación: Cada sujeto participará en el ensayo durante aproximadamente 12 meses, contados a partir del momento de la firma del formulario de consentimiento informado. Luego de una fase de tamizaje de aproximadamente 14 días, los sujetos que cumplan con los criterios de inclusión y no tengan criterios de exclusión, serán aleatorizados para asignarlos a uno de los tres grupos del estudio.

Duración del estudio: La duración total de la participación de los pacientes en el estudio será de doce meses, considerando dos semanas para el tamizaje y la evaluación según las directrices del PCC, ocho semanas de tratamiento con ATO, placebo y fármacos antichagásicos en sus respectivos grupos. La administración de ATO y placebo continuará durante un período adicional de ocho semanas. Habrá visitas de seguimiento hasta 12 meses después del inicio del tratamiento.

Tamaño muestral: El ensayo contempla una muestra de 300 pacientes, asignando de manera aleatoria 100 pacientes a cada grupo del estudio:

Atorvastatina 40 mg;

Atorvastatina 80 mg;

Placebo (Suplemento nutricional en comprimidos)

Criterios de Inclusión:

Adultos mayores de 18 años y menores de 50, con un peso superior a 40 kg, con una serología confirmatoria convencional para la infección por *T. cruzi* del Instituto de Salud Pública de Chile (ISPCH) o un centro de referencia autorizado, y un qPCR positivo serán seleccionados para participar en este ensayo. Solo se incluirá pacientes con diagnóstico nuevo de enfermedad de Chagas en la fase crónica

indeterminada.

Además, deberán cumplir con TODOS los criterios que se indican a continuación para ingresar al estudio:

- Cada sujeto debe ser mayor de 18 y menor de 50 años
- Cada sujeto debe tener una serología positiva para *T. cruzi* confirmada por el Instituto de Salud Pública de Chile (ISPCH) o un centro de referencia autorizado
- Tener un examen de qPCR positivo para *T. cruzi*
- Tener valores normales en las pruebas de laboratorio para los siguientes parámetros: recuento total de glóbulos blancos, recuento de plaquetas, creatina kinasa (CK), alanina aminotransferasa (ALT), aspartato aminotransferasa (AST), bilirrubina total o creatinina, o una gamma-glutamil transferasa (GGT) <= 2 veces el límite superior de la normalidad (X ULN);
- Las mujeres en edad reproductiva deben tener una prueba de embarazo en suero negativa, no deben estar amamantando y deben utilizar sistemáticamente un método anticonceptivo altamente eficaz durante toda la fase de tratamiento.
- Tener la capacidad de cumplir con todas las pruebas y visitas de seguimiento especificadas en el protocolo y tener una dirección permanente;
- Firmar el Formulario de Consentimiento Informado

Criterios de Exclusión:

- Signos y síntomas de la forma digestiva de la EC;
- EC cardiaca crónica en estadio II o superior;
- Condiciones de salud agudas o crónicas como infecciones agudas, antecedentes de infección por VIH, diabetes, enfermedades hepáticas y renales;
- Hipotiroidismo
- Historia familiar de alteraciones musculares
- Enfermedad cardiaca preexistente no relacionada con la enfermedad de Chagas;
- Contraindicación formal para recibir NFX o BZD,
- Antecedentes conocidos de hipersensibilidad, alergia o reacciones adversas graves a ATO, BZD o NFX;
- Antecedentes de tratamiento previo para la EC;
- Antecedentes de tratamiento previo con atorvastatina, lovastatina, rosuvastatina, simvastatina o cualquier otra estatina;
- Cualquier uso concomitante de agentes antimicrobianos;
- Antecedentes de abuso de alcohol o drogas;
- Cualquier condición que impida la medicación oral;
- Uso concomitante o previsto de modificadores del CYP3A4;
- Antecedentes médicos de síndrome de QT corto familiar o terapia concomitante con medicamentos que puedan acortar el intervalo QT.
- Valores anormales en las pruebas de laboratorio para los siguientes parámetros: recuento total de glóbulos blancos, recuento de plaquetas, creatina kinasa (CK), alanina aminotransferasa (ALT), aspartato aminotransferasa (AST), bilirrubina total o creatinina, o una gamma-glutamil transferasa

(GGT) > 2 veces el límite superior de la normalidad (X ULN);

- Estar embarazada o en período de lactancia
- Negarse a usar un método anticonceptivo altamente eficaz durante la fase de tratamiento.

**Fármacos prohibidos antes de administrar los tratamientos del estudio y durante el estudio.**	**Período de espera[a]**
Terapia Sistémica antimicótica.	30 días
Otros fármacos en experimentación (nuevas entidades químicas o biológicas).	30 días
Medicamentos con interacción conocida con estatinas y que podrían provocar eventos adversos potencialmente peligrosos: fibratos como gemfibrozilo, ciclosporina, fenofibrato.	24 horas
Inhibidores de bomba de protones.[b]	10 días
Medicamentos que modifican los niveles de estatinas: carbamazepina, eritromicina, claritromicina, colchicina, rifampicina, hierba de san Juan (*Hypericum perforatum*).	24 horas
Inmunosupresores o dosis suprafisiológicas de glucocorticoides.[c]	24 horas

CYP3A4 = citocromo P450 3A4; HMG-CoA=beta-hidroxi-beta-metil-glutaril-CoA

[a] Estos tiempos de espera deben observarse antes del inicio del tratamiento del estudio. No se permite el uso concurrente de estos fármacos. Cualquier cambio en estos períodos deben ser aprobados por el investigador principal antes del uso de los fármacos del estudio o del agente prohibido.

[b] Se permite el uso de antagonistas H_2.

[c] Excepto para el tratamiento a corto plazo del asma, enfermedad pulmonar obstructiva crónica (EPOC), Hiperreactividad de las vías aéreas o reacciones de hipersensibilidad o erupciones alérgicas.

PRODUCTOS INVESTIGACIONALES; DOSIS Y FORMA DE ADMINISTRACION

PRODUCTOS INVESTIGACIONALES:

Atorvastatina administrada por vía oral una vez al día en dosis de 40 u 80 mg.

PRODUCTOS DE REFERENCIA:

Placebo por vía oral una vez al día**.** El placebo se justifica en tanto el paciente recibe el tratamiento antichagásico convencional completo y por lo tanto se asegura el manejo de su enfermedad de Chagas

La terapia antichagásica (nifurtimox o benznidazol) se administrará a todos los sujetos del estudio, indistintamente del grupo de estudio al cual estén asignados.

Análisis de eficacia:

Hipótesis: Atorvastatina en combinación con la terapia antichagásica convencional de nifurtimox o benznidazol mejora la función cardiaca en sujetos con enfermedad de Chagas en fase crónica asintomática en comparación con un placebo.

Para el **análisis del resultado primario**, se utilizará una prueba exacta de Fisher unilateral de la proporción de pacientes con una disminución significativa de los niveles de biomarcadores en las poblaciones ITT (análisis primario), y por protocolo (análisis secundario). Se realizará la prueba ANOVA para analizar las diferencias de las varianzas entre los distintos marcadores.

Para todas las **comparaciones secundarias** de proporciones entre ATO frente a placebo, incluyendo la respuesta parasitológica al tratamiento, se realizará una prueba exacta. Se utilizarán análisis de clases

latentes y multivariantes para evaluar la asociación entre la respuesta parasitológica, los cambios en los biomarcadores y la dosis de ATO.

Si no hay diferencias significativas entre los tratamientos con ATO, estos datos se colapsarán en un único grupo de comparación para obtener mayor precisión en el estimado del efecto del tratamiento, dado que este es un estudio de prueba de concepto para estatinas como clase. En este caso, no se hará ajustes para comparaciones múltiples.

Análisis de Seguridad: Las evaluaciones clínicas, el electrocardiograma, y la revisión de los exámenes de laboratorio serán usados para monitorear la seguridad y tolerancia a los fármacos bajo estudio a lo largo de todo el período de observación de los sujetos.

Se describirá la proporción de pacientes que presentan al menos un EA. Se presentará la tasa de incidencia y el intervalo de confianza del 95% por grupo del estudio. Por lo demás, sólo se presentarán estadísticas descriptivas. Los parámetros de seguridad de laboratorio (hematología y bioquímica) también se describirán individualmente por cada grupo del estudio, mostrando la proporción de pacientes por grado de elevación en relación con el ULN y con los valores iniciales, y los cambios de los niveles sanguíneos a lo largo del tiempo

Diagrama del Diseño Experimental

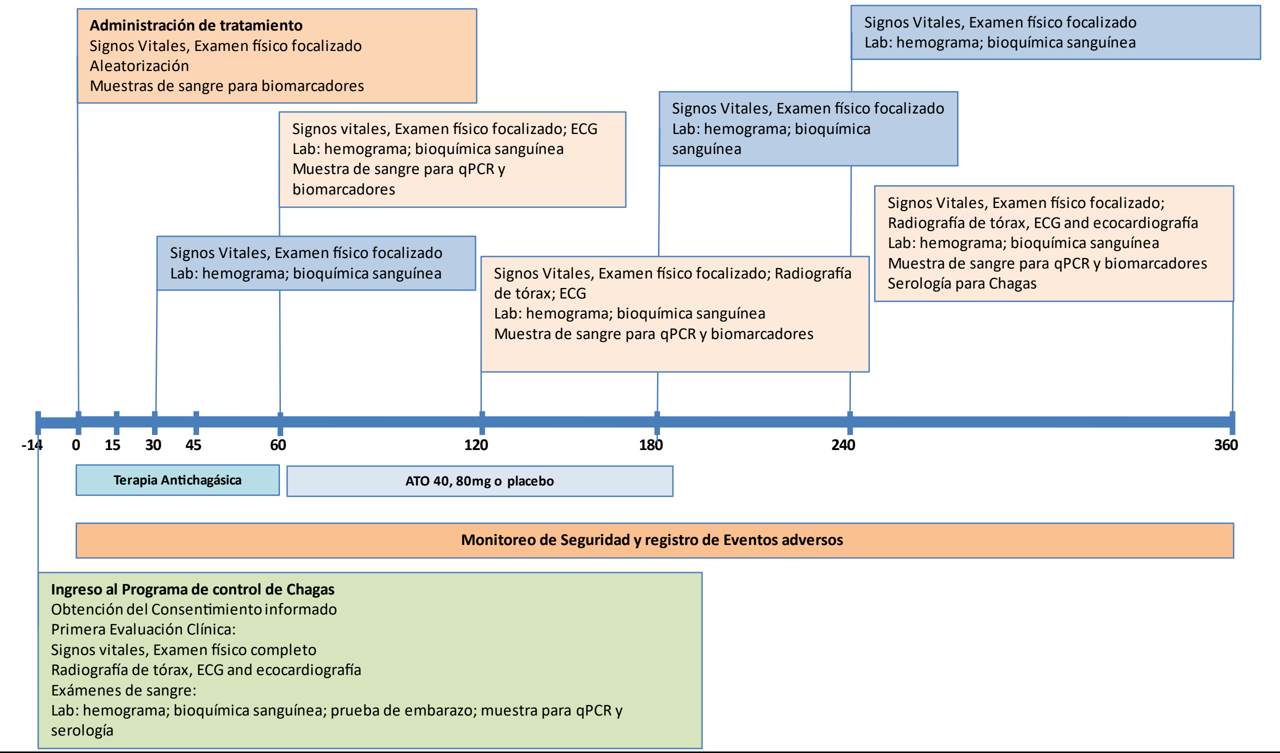

Diagrama de Flujo del ensayo

Fase	Pre-Aleatorización		Aleatorización					
Período	Tamizaje	Línea de base	Tratamiento				Seguimiento	
Visita	1	2	3	4	5	6	7	8
Día(s)	-14 a -1	0	30	60	120	180	240	360
**Procedimiento**								
Consentimiento Informado	x							

Aleatorización		x						
Ficha Clínica	x							
Fármacos previos/concurrentes	←---→							
Inclusión/Exclusión	x	x						
Signos Vitales	x	x	x	x	x	x	x	x
Examen físico completo	x				x			x
Radiografía de tórax	x				x			x
ECG	x			x	x	(x)[e]	(x)[e]	x
Ecocardiograma 2D	x							x
Prueba de Embarazo	x					x	x	
Serología	x							x
Laboratorio[a]	x	x	x	x	x	x	x	x
PCR cuantitativo	x				X[d]			x
Biomarcadores[b]		X		x	x			x
Examen Físico focalizado [c]	x	x	x	x	x	x	x	x
Eventos Adversos				←--------------------------------→				
Contabilidad de fármacos		x	x	x	x			

[a]Los parámetros de laboratorio incluirán: hemoglobina, recuento total de glóbulos blancos, recuento diferencial de glóbulos blancos y recuento de plaquetas. Los parámetros bioquímicos de laboratorio incluirán: CK, ALT, AST, GGT, fosfatasa alcalina, bilirrubina total y directa, un perfil lipídico: colesterol total, c-DHL y c-LDL, triglicéridos, glucemia en ayunas y creatinina;
[b]Biomarcadores: BNP, cTnT, IFN-γ, IL-1β, IL-4, IL-17A, e IL-10, sICAM-1, sVCAM-1, sE-selectina;
[c]Examen físico centrado sólo en la evaluación de eventos adversos;
[d]Las pruebas de PCR en estos puntos temporales se harán con una sola muestra de 10 ml;
[e]los ECG se realizarán en estas visitas sólo en caso de anomalías previamente identificadas

Contenido

1. Código del proyecto 1
3. Título del estudio Clínico 1
4. Equipo de investigación 1
6. RESUMEN 2
7. Justificación y antecedentes 12
7.1. La enfermedad de Chagas 12
7.1.1. Aspectos clínicos de las fases aguda y crónica de la EC 12
7.1.2. Diagnóstico 12
7.1.3. La cardiopatía chagásica es una enfermedad inflamatoria 13
7.1.3.1. Daño miocárdico inmunomediado dependiente del parásito 13
7.1.3.2. Compromiso microvascular en la EC 14
7.1.3.3. Alteraciones de la perfusión regional 14
7.1.3.4. Disfunción endotelial 14
7.1.4. Tratamiento de la miocardiopatía chagásica crónica y estrategias de mejora 15
7.2. Papel de las estatinas en el proceso inflamatorio durante la enfermedad de Chagas 16
8. OBJETIVO Y FINALIDAD DEL ENSAYO 18
8.1. Objetivo primario 18
8.2. Objetivos secundarios 18
9. Diseño del Estudio 19
9.1. Resultados 19
9.2. Análisis estadístico 20
9.3. Descripción del estudio 20
9.4. Población de estudio 21
9.5. Duración del estudio y duración de la participación de los sujetos 21
9.6. Calendario de eventos para cada sujeto durante la fase de reclutamiento 21
9.7. Evaluaciones 22
9.8. Diagrama general del diseño del estudio 24
10. SELECCIÓN Y RETIRADA DE SUJETOS 24
10.1. Criterios de inclusión 24
10.2. Criterios de exclusión 25
11. TRATAMIENTO DE LOS SUJETOS 25

11.1.1. Dosis y regímenes de tratamiento 25
11.1.2. Fármacos prohibidos antes de administrar los tratamientos del estudio y durante el estudio 26
11.2. Eventos adversos y retiro del estudio 26
11.2.1. Eventos adversos 26
11.2.2. Evento adverso grave (EAG) 27
11.2.3. Calificación de la gravedad de los eventos adversos 28
11.2.4. Evaluación de la causalidad de los eventos adversos 28
11.2.5. Retiro del estudio 29
11.2.6. Procedimiento de apertura del ciego 30
12. Análisis de datos y métodos estadísticos 30
12.1. Tamaño de la muestra 30
12.2. Aleatorización y asignación del tratamiento 30
12.3. Poblaciones a analizar: 30
12.4. Análisis de eficacia 30
12.5. Análisis de seguridad: 31
12.6. Criterios Término anticipado del estudio 31
13. Ética 31
14. Proceso de consentimiento informado 32
14.1. Costes de los pacientes 32
15. Acceso directo a los datos y documentos fuente: 32
16. Control y garantía de calidad 32
16.1. Formularios de Registro Individual (CRF) 32
16.2. Documentos fuente 33
17. Manejo de los datos y archivo de los registros 33
18. Informes y publicación 34
19. Bibliografía 35

7. Justificación y antecedentes:

Postulamos que las estatinas, como la atorvastatina, por su efecto antiinflamatorio, mejoran la inflamación vascular y cardíaca en pacientes con enfermedad de Chagas crónica asintomática, contribuyendo a la recuperación de la función endotelial y cardíaca y, mejorando la eficacia del tratamiento farmacológico antichagásico de la enfermedad de Chagas.

7.1. La enfermedad de Chagas:

Importancia del problema: El protozoo flagelado *Trypanosoma cruzi* es el agente etiológico de la enfermedad de Chagas (EC). Este parásito se transmite al ser humano por medio de insectos vectores (*Triatoma infestans*, alias 'vinchuca'), transfusión de sangre infectada o de una madre infectada a su hijo, que es actualmente la principal vía de transmisión en Chile (MINSAL, 2017). Es un mal que afecta a 7 millones de personas en 21 países endémicos de América Latina y está aumentando en los países no endémicos debido a la migración (OMS, 2015). La EC es la segunda enfermedad con mayor carga de morbilidad entre las Enfermedades Tropicales (OMS, 2011) con años de vida ajustados por discapacidad (AVAD) de 806.170. Estudios recientes estiman que el costo económico global de la EC en el mundo supera los USD$ 7 mil millones anuales (Requena-Méndez et al., 2017). Esta cifra supera la del rotavirus (USD$ 2 mil millones) y la del cáncer cervical (USD$ 4,7 mil millones) (Lee et al., 2013; Schmunis, 2013). La EC produce aproximadamente 8.000 muertes anuales, cifra superior a la de la malaria en las Américas (OMS, 2018). La principal estrategia de control de la EC incluye el control de los vectores, el tamizaje sistemático de los donantes de sangre en todos los países endémicos, la detección y el tratamiento de la transmisión congénita, y el tratamiento de los niños infectados y de los casos agudos (OMS, 2015). Sin embargo, los programas de control son discontinuos y la terapia actual es limitada debido a su baja eficacia (OMS, 2015). Esta es la razón por la que el objetivo de la OMS de erradicar o controlar la EC en 2020 estuvo lejos de alcanzarse (Lenk et al., 2018).

7.1.1. Aspectos clínicos de las fases aguda y crónica de la EC:

Clínicamente, la EC presenta una fase aguda, diagnosticada en menos del 10% de los casos, debido a la ausencia o presencia de síntomas leves. El curso clínico, en la mayoría de los casos, es hacia la recuperación espontánea (Berna, 2015), pero sin la eliminación del parásito. La fase crónica puede ser asintomática (indeterminada), dura de 10 a 30 años y se caracteriza por una serología positiva y cierto grado de afectación cardíaca evidenciada por una inflamación mínima pero persistente. Sin embargo, en alrededor del 30% de los pacientes, progresa a las formas sintomáticas de la enfermedad, con compromiso del esófago, colon o corazón (Rassi et al., 2010). Los síntomas y signos físicos de la miocardiopatía chagásica crónica (MCC) surgen por insuficiencia cardíaca, arritmias cardíacas (como arritmias ventriculares, bloqueo auriculoventricular, taquicardia supraventricular o fibrilación auricular) (Jefferies y Towbin, 2010) y el tromboembolismo arterial o venoso (Rassi et al., 2017a).

7.1.2. Diagnóstico:

El diagnóstico clínico de la EC en la fase aguda es difícil de realizar ya que la gran mayoría de las personas infectadas no presentan síntomas. Sin embargo, es posible detectar el parásito en la sangre del paciente, identificando los tripomastigotes en la capa leucocitaria o mediante una

reacción en cadena de la polimerasa (PCR) convencional. En la fase crónica, el diagnóstico se basa en datos clínicos y epidemiológicos, junto con pruebas serológicas como un ELISA IgG de alta sensibilidad e inmunofluorescencia indirecta IgG (Jercic y Oyarce, 2019; MINSAL, 2017). Si hay sospecha de compromiso gastrointestinal, se deben realizar estudios radiológicos abdominales. La evaluación cardíaca es esencial cuando se hace el diagnóstico para proporcionar una clasificación funcional y estratificar un riesgo de mortalidad a diez años (Nunes et al., 2018; Rassi et al., 2006; Rassi, 2010). La clasificación funcional ayuda a explicar la evolución de la EC. Se basa en criterios clínicos, la clasificación funcional de la NYHA, radiografía de tórax, electrocardiograma (ECG), ecocardiograma, Holter, tromboembolismo y criterios de muerte súbita, y describe los estadios I a IV (Rassi, 2010). El ECG es útil para detectar trastornos de la conducción, como el bloqueo auriculoventricular o de rama derecha, que pueden aparecer antes de que se manifiesten los síntomas (Rojas et al., 2018). Se debe realizar un Holter de 24 horas en caso de arritmias. Una radiografía de tórax es útil para determinar el índice cardiotorácico indicativo de cardiomegalia. También se recomienda al menos una evaluación ecocardiográfica, especialmente cuando el ECG es anormal (MINSAL, 2017).

Se han propuesto diferentes biomarcadores para evaluar la progresión, el pronóstico o la respuesta al tratamiento; pero, ninguno ha demostrado suficiente especificidad para ser incorporado como estándar de oro para el diagnóstico de la enfermedad de Chagas (Cortes-Serra et al., 2020). Sin embargo, el péptido natriurético cerebral (BNP) y la troponina T cardiaca (cTnT) se han propuesto como biomarcadores útiles para predecir la evolución hacia la disfunción ventricular izquierda (Echeverría et al., 2020). Por otro lado, la detección del DNA del parásito es un marcador útil sólo para determinar el fracaso del tratamiento, ya que un resultado negativo no excluye la persistencia del parásito (Alonso-Padilla et al., 2017; Parrado et al., 2019).

7.1.3. La cardiopatía chagásica es una enfermedad inflamatoria:

Los principales mecanismos patogénicos que explican la CCC son: i) daño miocárdico inmunomediado dependiente del parásito, que es el determinante más crítico de la enfermedad (Rassi et al., 2017b), donde la respuesta $TH_1/TH_2/T_{Reg}$ es una característica crucial (Acevedo et al., 2018); ii) anomalías microvasculares e isquemia (Borges et al., 2018); y iii) disautonomía cardíaca secundaria a mecanismos inmunitarios autorreactivos (Booney et al., 2019).

7.1.3.1. Daño miocárdico inmunomediado dependiente del parásito:

Durante la primoinfección, poco después de la invasión de los macrófagos, las células NK estimuladas con IL-12/TNF-α producen un pico de secreción de IFN-γ para controlar la replicación del parásito a expensas de disminuir la producción de IL-10 (Cardillo et al., 1996). La producción de IL-12 contribuye además a polarizar la respuesta inmunitaria hacia una amplia diversidad de perfiles pro y antiinflamatorios (Hasegawa et al., 2002), lo que explica, en parte, la evasión de la respuesta inmunitaria.

Debido a la persistencia del parásito, el destino del huésped es hacia un estado de activación del sistema inmunitario de bajo grado, pero permanente. En ese escenario, la respuesta de las células T es esencial para el mantenimiento de la parasitemia, típicamente baja, en la fase crónica de la enfermedad. Sin embargo, existe una respuesta citotóxica defectuosa de las células T en la CCC con el agotamiento gradual de la población de células T CD8+ (Pérez-Anton et al., 2020). Además,

el equilibrio TH1/TH2 es esencial para la evolución hacia la CCC. El parásito induce una respuesta combinada donde el equilibrio entre el exceso de citoquinas proinflamatorias (IFN-γ, TNF-α, IL-1β) y antiinflamatorias (IL-4, IL-10) puede ser crítico en el desarrollo del CCC (Acevedo et al., 2018). Mención especial merece el subconjunto TH17 ya que los niveles elevados de IL-17A pueden correlacionarse con una enfermedad cardíaca más leve (Sousa et al., 2017).

A partir de lo anterior, los niveles de IFN-γ, IL-1β, IL-4, IL-17A e IL-10 podrían ayudar a predecir la progresión de la EC y su resultado tras la terapia farmacológica (Llaguno et al., 2019), y la inclusión de fármacos antiinflamatorios podría inclinar favorablemente hacia un mejor perfil de respuesta inmune y mejorar la terapia de la CCC.

7.1.3.2. Compromiso microvascular en la EC:
Existen evidencias tanto experimentales como clínicas de alteraciones microvasculares coronarias que conducen a daño miocárdico isquémico en animales infectados con *T. cruzi* y en pacientes con CCC (Rossi et al., 2010). Se ha informado por los autores y otros que las alteraciones microvasculares relacionadas con i) alteraciones de la perfusión regional (Lemos de Oliveira et al., 2018), ii) la activación de las plaquetas (González-Herrera et al., 2017; Pengue et al., 2019), y iii) disfunción endotelial (Campos-Estrada et al., 2015) en corazones de ratones infectados crónicamente por *T. cruzi*, son similares a las observadas en las miocardiopatías isquémicas (Borges et al., 2018).

7.1.3.3. Alteraciones de la perfusión regional:
Hasta un 20% a 30% de los pacientes con CCC se quejan de dolor torácico que se asemeja a la angina de pecho en cuanto a su localización y carácter, sin relación con el esfuerzo, y con las arterias subepicárdicas sanas (Lemos de Oliveira et al., 2018). Además, existen defectos de perfusión miocárdica reversibles, que pueden revertirse con dipiridamol (Tanaka et al., 2019). Estos defectos pueden ser atribuidos a la presencia de inflamación alrededor de las arterias coronarias intramurales causando la redistribución del flujo coronario de las regiones isquémicas a las no isquémicas, induciendo así la aparición del cuadro clínico consistente con la disfunción microvascular coronaria (Bestetti y Restini, 2014). Este aspecto puede estar correlacionado con la denervación autonómica (Barizon et al., 2020), incluso antes de que aparezca la fibrosis.

7.1.3.4. Disfunción endotelial:
La alteración de la perfusión puede ser una consecuencia directa del daño microvascular secundario a la activación endotelial inducida por la infección por *T. cruzi* y la posterior interacción con las células efectoras inmunitarias. Las células endoteliales (CE) activadas producen diversas citoquinas, incluyendo las moléculas inductoras de vasoconstricción, endotelina-1 y tromboxano A2 (TXA2) (Hernández et al., 2018). Como demostramos anteriormente, las CE también aumentan la producción de moléculas de adhesión celular como las Moléculas de Adhesión Intercelular tipo 1 (ICAM-1), Moléculas de Adhesión Celular Vascular (VCAM) y E-selectina, incluyendo una forma soluble de las moléculas de adhesión celular (sCAM) que se encuentran en sangre (Campos-Estrada et al., 2015; González-Herrera et al., 2017; Molina-Berrios et al., 2013b). También demostramos que la inducción de las moléculas de adhesión está mediada por la activación de cascadas de señalización inflamatoria (Campos-Estrada et al., 2015), amplificando el aporte inflamatorio inicial. Además, la producción de citoquinas desde la CE inmune y activada facilita la

migración de células inflamatorias y monocitos al subendotelio, donde producen una respuesta inflamatoria local, aumentando el daño endotelial (González-Herrera et al., 2017; Marín-Neto et al., 2013) y las alteraciones de la perfusión mencionadas anteriormente. Todas estas moléculas son potenciales biomarcadores para la predicción de trastornos trombóticos o endoteliales (Cortes-Serra et al., 2020). En particular, la E-selectina puede ser un excelente candidato, ya que se expresa exclusivamente en las CE vasculares (Dzikowska-Diduch et al., 2017).

Por lo tanto, los trastornos de la perfusión miocárdica, debidos al daño endotelial y a las alteraciones microcirculatorias, contribuyen a la progresión de la disfunción segmentaria del VI observada en la fase crónica de la ECC (Hiss et al., 2009). Cualquier enfoque terapéutico para mejorar la función del ventrículo izquierdo o prevenir estas alteraciones vasculares podría ser beneficioso para los pacientes con EC.

En general, teniendo en cuenta el papel del endotelio en el inicio y la propagación de la lesión de la pared vascular en la EC, es necesario evaluar los tratamientos farmacológicos capaces de inhibir la activación, la disfunción y la lesión vascular de las CE en los pacientes con EC crónica.

7.1.4. Tratamiento de la miocardiopatía chagásica crónica y estrategias de mejora:

En Chile, el tratamiento etiológico de la EC se realiza con 5-10 mg/kg/día de nifurtimox (NFX) o 5 mg/kg/día de benznidazol (BZD) durante 60 días (MINSAL, 2017). Todos los pacientes deben recibir tratamiento antichagásico sin exclusión ni demora, excepto durante el embarazo y la lactancia, aunque hay evidencia reciente que sugiere que NFX podría ser seguro en estas condiciones (Moroni et al., 2019). Asimismo, la hipersensibilidad a los fármacos tripanocidas excluye su uso. Otras exclusiones son las enfermedades renales, hepáticas o cardíacas severas, incluyendo CCC con compromiso estructural, alcoholismo (efecto *Antabuse* de NFX) y megacolon o megaesófago severo (MINSAL, 2017).

El tratamiento de la EC se asocia a un aumento del riesgo de eventos adversos (EA) graves, con una incidencia media del 16,20% (OPS, 2018), situación que puede obligar a suspender la terapia hasta en el 48% de los pacientes que reciben NFX (Crespillo-Andujar et al., 2018a; Crespillo-Andujar et al., 2018b; Jackson et al., 2020). Los EA incluyen pérdida de peso superior al 5-15%, trastornos digestivos (náuseas, vómitos, diarrea, epigastralgia y dispepsia, distensión abdominal), cefaleas, irritabilidad, insomnio, trastornos del estado de ánimo, pérdida de memoria, fiebre, artralgias, mialgias, erupciones cutáneas y de mucosas grave o significativa como el síndrome de Stevens Johnson, evidencia de trombocitopenia, pancitopenia o signos de depresión de la médula ósea, elevación de las transaminasas, leucopenia por debajo de 2500/mm^3, trastornos del SNC como paresias, parálisis o convulsiones, y cambios de comportamiento, especialmente en el caso del NFX.

El tratamiento farmacológico durante la fase aguda, la enfermedad congénita y la fase indeterminada temprana tiene una eficacia satisfactoria y se considera curativo (Sales Junior et al., 2017). Sin embargo, es más difícil declarar la curación en la infección crónica porque la evidencia actual de la eficacia de los fármacos en esta fase es débil o controvertida, especialmente cuando se considera la mortalidad (OPS, 2018). Tras cinco años de seguimiento, el BZD no pudo reducir significativamente el deterioro clínico cardíaco en pacientes con enfermedad cardíaca leve (Morillo et al., 2015). Además, se requiere un seguimiento a largo plazo para confirmar la

seroreversión, por lo que la serología es un sustituto débil como criterio de curación en la EC crónica (Sguassero et al., 2018). Las terapias novedosas, como los antifúngicos posaconazol o ravuconazol, no tuvieron éxito (Molina et al., 2014; Molina et al., 2017; Torrico et al., 2018). Así, los ensayos clínicos rigurosos no han demostrado evidencia convincente de beneficio clínico o erradicación de la parasitemia en la EC crónica.

Por lo tanto, es de suma importancia encontrar enfoques innovadores para tratar esta enfermedad tropical desatendida que amenaza la vida. Tres consideraciones apoyan esta afirmación: i) la terapia actual para la EC en la fase crónica es infructuosa y aumenta el riesgo de toxicidad sistémica, EA y mala adherencia al tratamiento o incumplimiento; ii) ninguno de los muchos compuestos tripanocidas potenciales enumerados es superior al NFX o al BZD (Ribeiro et al., 2020); y iii) la mayoría de los fármacos antiparasitarios se consideran huérfanos debido al bajo retorno de los costes de desarrollo para la industria farmacéutica (Varela y Fernandes, 2020). *Por lo tanto, la mejora de la eficacia de los actuales fármacos antiparasitarios mediante la modificación de los factores del huésped, como la respuesta inflamatoria -principalmente mediante la reutilización de fármacos existentes- haría que el tratamiento actual fuera más eficaz*.

7.2. Papel de las estatinas en el proceso inflamatorio durante la enfermedad de Chagas crónica

Existen moléculas que participan en la resolución natural de la inflamación. Estos mediadores pro-resolución especializados incluyen varios lípidos que controlan la magnitud y la duración de la inflamación local (Serhan y Chiang, 2013). Estos lípidos se derivan de ácidos grasos esenciales presentes en la membrana plasmática, como el ácido araquidónico o el ácido docosahexaenoico. Curiosamente, la aspirina y las estatinas reductoras del colesterol, incluida la atorvastatina (ATO), pueden S-nitrosilar la enzima ciclooxigenasa-2 (COX2), en el aminoácido 298 que le permite producir precursores ya no para la síntesis de prostaglandinas (que son proinflamatorias), sino epímeros que, mediante la acción de la 5-lipooxigenasa (5-LO), darán lugar a moléculas más estables, como la 15-epi-lipoxina A4 (LXA4) o la resolvina D1 activada por la aspirina (AT-RvD1)(Serhan, 2017; Spite y Serhan, 2010).

El investigador responsable y su equipo realizaron varios estudios preclínicos con resultados que sugieren que la aspirina y la simvastatina disminuyen la inflamación cardíaca y la activación endotelial en ratones infectados crónicamente por *T. cruzi* (López-Munoz y otros, 2010; Molina-Berrios y otros, 2013b; Molina-Berrios y otros, 2013c). La 15-epi-LXA4 media esta acción (Campos-Estrada et al., 2015; González-Herrera et al., 2017; Molina-Berrios et al., 2013a), en un proceso que implica una disminución de la actividad de la vía proinflamatoria NF-κB (Campos-Estrada et al., 2015). Además, la simvastatina mejora, independientemente de la actividad de 15-epi-LXA4, la función ventricular en ratones BALB/c infectados crónicamente (Guzmán-Rivera et al., 2020), mientras que los estudios en curso muestran que la resolvina D1 gatillada por aspirina/estatina (AT-RvD1) mejora la función eléctrica cardíaca, en ausencia de tratamiento tripanocida.

De lo anterior se desprende que *una combinación de fármacos tripanocidas y aquellos que inducen la resolución del proceso inflamatorio derivado de la persistencia del parásito podría ser una buena estrategia terapéutica para prevenir las consecuencias crónicas de la EC*.

Aunque las evidencias preclínicas presentadas sugieren que una estatina podría tener un efecto beneficioso sobre la EC crónica, esta materia no ha sido probada en el ámbito clínico. Solo hay una

mención al uso de rosuvastatina en el tratamiento de la EC, pero la calidad de la evidencia aportada es pobre (Marti-Carvajal y Kwong, 2016). Otros ensayos clínicos, pero centrados únicamente en el tratamiento sintomático de la insuficiencia cardíaca asociada a la EC han probado a la amiodarona (Stein et al., 2018), el carvedilol (Botoni et al., 2007) y fármacos moduladores de la angiotensina (Souza-Silva et al., 2019). Otros fármacos estudiados en pequeños ensayos clínicos son el alopurinol, el itraconazol o el ketoconazol con resultados mixtos (Apt et al., 1998; Apt et al., 2013; Brener et al., 1993); lamentablemente, faltan más ensayos clínicos con estos fármacos. Un obstáculo importante para el desarrollo de fármacos es la escasa traslación de los datos *in vivo* a la enfermedad humana. Así, **nuestra propuesta tiene como objetivo analizar si una estatina mejora la eficacia del tratamiento antichagásico en el contexto de la Enfermedad de Chagas Crónica a través de un ensayo clínico doble ciego de fase II y evaluar los parámetros inflamatorios asociados a la mejora observada**.

Existe un acuerdo generalizado de que los adultos con EC crónica indeterminada son la población con las necesidades más urgentes para el desarrollo de nuevos tratamientos debido a la mayor carga de la enfermedad para estos pacientes. Así pues, la mejora de los factores del hospedero (por ejemplo, la reacción inmunitaria provocada) puede aumentar la eficacia del tratamiento antichagásico convencional, probablemente mediante disminución de las dosis, la reducción de su duración, o ambas cosas.

Dada la escasez de tratamientos basados en la evidencia en esta enfermedad, este estudio ofrecerá la oportunidad de evaluar el impacto de una estrategia terapéutica novedosa en una combinación de marcadores candidatos prometedores de inflamación, actividad endotelial y función cardíaca, asociados a una mejora sostenida, y correlacionar los resultados con los resultados parasitológicos. Por lo tanto, *este ensayo podría ser el primer paso para evaluar una terapia potencial que se propone modificará favorablemente el curso de la EC crónica indeterminada*.

Aunque nuestros estudios preclínicos se realizaron con simvastatina, aquí proponemos la atorvastatina (ATO) como estatina experimental. Como la simvastatina está asociada a la ezetimibe, no se tendrá en cuenta en este estudio. Los perfiles terapéuticos y de seguridad de la ATO son bien conocidos, así como su mecanismo de acción y sus acciones farmacológicas, incluidas sus propiedades antiinflamatorias, que comparten los demás miembros de la clase de las estatinas. Es importante destacar que, debido a la baja incidencia de EA graves y a su eficacia, ambas son las estatinas más utilizadas en la actualidad. Se utilizan 20-80 mg/día de ATO para disminuir el llamado colesterol LDL, implicado en la patogénesis de la enfermedad cardiovascular aterosclerótica. No obstante, es imprescindible realizar un estudio de prueba de concepto en fase clínica II porque: i) es una forma de trasladar los resultados obtenidos en el modelo animal, ii) es necesario evaluar su dosis mínima efectiva y su seguridad en el contexto del paciente en tratamiento de la EC crónica y, lo que es más importante, iii) porque las estatinas no están actualmente aprobadas para el tratamiento de la EC.

Considerando i) la elevada carga de la enfermedad; ii) el papel del endotelio y la inflamación en el inicio y la propagación de la lesión de la pared vascular en la EC; iii) que la terapia actual para la EC en la fase crónica no tiene éxito y aumenta el riesgo de toxicidad sistémica, EA y mal

cumplimiento; iv) que la mejora de la eficacia de los fármacos antichagásicos actuales mediante la modificación de las respuestas del huésped haría más eficaz el tratamiento actual; v) que las evidencias preclínicas sugieren que las estatinas pueden tener un papel en la prevención del daño cardíaco en la EC; vi) que las estatinas son seguras y con un perfil farmacológico bien conocido; entonces, una combinación de fármacos tripanocidas con aquellos que inducen la resolución del proceso inflamatorio derivado de la persistencia del parásito podría ser una estrategia terapéutica eficaz para prevenir las consecuencias crónicas de la EC. En consecuencia, se propone que la siguiente **hipótesis**:

En pacientes con Enfermedad de Chagas Crónica, las estatinas mejoran la eficacia del tratamiento antiparasitario al disminuir la inflamación y mejorar la función endotelial y cardíaca

8. OBJETIVO Y FINALIDAD DEL ENSAYO

8.1. Objetivo primario:

Evaluar el uso de la atorvastatina en combinación con la terapia antiparasitaria (NFX o BZD), es segura y más eficaz que la terapia antiparasitaria sola en prevenir la aparición de alteraciones cardiacas mediante la reducción de la inflamación general y en la mejora de las funciones endotelial y cardíaca.

Para valorar el objetivo primario se tendrá en cuenta el número de pacientes que presentan un cambio en la fase de la cardiomiopatía crónica en 12 meses desde el momento del inicio del tratamiento antichagásico.

Se va a evaluar si el efecto de la atorvastatina en combinación con la terapia antiparasitaria (NFX o BZD), es más eficaz que la terapia antiparasitaria sola (grupo placebo) en la prevención de la aparición de trastornos cardíacos determinados por la no progresión en la fase desde la fase A según las I Guías Latinoamericanas para el diagnóstico y tratamiento de la cardiomiopatía chagásica (Andrade et al, Arq Bras Cardiol 2011;97 Suppl 3:1-48.). Para hacer esta valoración se tendrán en cuenta los cambios significativos en: i) electrocardiograma (Frecuencia cardiaca, y duración del intervalo QT, así como aparición de trastornos de la conducción eléctrica, determinado por duración y morfología del segmento QRS), ii) Fracción de eyección (valorada mediante ecocardiografía), iii) Tamaño de silueta cardiaca (radiografía de tórax).

8.2. Objetivos secundarios:

8.2.1. Evaluar la eficacia de la combinación de ATO y terapia antichagásica para disminuir:

- La inflamación, medida por los niveles plasmáticos de las citocinas, TNF-α, IFN-γ, IL-10, IL-1B, IL-4 e IL-17A.
- La activación endotelial, medida por los niveles plasmáticos de sCAM: sE-selectina, sICAM-1 y sVCAM-1.
- El daño y la función cardíaca: medidos por los niveles plasmáticos de BNP, y cTnT, el ECG de 12 derivaciones en reposo (frecuencia cardiaca, duración segmento QT y cambios en conducción eléctrica determinado por duración y morfología del segmento QRS), y el ecocardiograma 2D (Fracción de eyección).

8.2.2. Determinar la seguridad y tolerabilidad de la combinación de ATO con la terapia antichagásica, medida por la incidencia de EA (por ejemplo, rabdomiólisis) y la tasa de interrupción de la terapia.
8.2.3. Evaluar la respuesta al tratamiento de la combinación de ATO con la terapia antichagásica, medida por la PCR cuantitativa y la serología, durante un período de seguimiento de diez meses.
8.2.4. Determinar el grado de cumplimiento de las terapias mediante la medición de la contabilidad de los medicamentos a lo largo del estudio.

9. Diseño del Estudio:

9.1. Resultados:

Como en cualquier ensayo clínico, los resultados deben estar claramente definidos previamente. Así, el **resultado primario** de este estudio a los doce meses es el cambio en la función cardíaca en pacientes con EC crónica. Para ello, se evaluará el número o proporción de pacientes que presentan un cambio en la fase de la cardiomiopatía crónica. Este cambio de fase se determina por la no progresión o reversión desde o hacia la fase A, según las I Guías Latinoamericanas para el diagnóstico y tratamiento de la cardiomiopatía chagásica (Andrade et al, 2011), considerando los hallazgos sugrentes de alteración estrucutral evidenciada por los cambios en los parámetros clínicos, electrocardiográficos, ecocardiográficos y, adicionalmente, de los niveles plasmáticos de los biomarcadores de función cardíaca BNP, cTnT.

Chart 1 – Clinical classification of left ventricular dysfunction in chagasic cardiopathy

Acute phase	Chronic phase				
	Indeterminate form	Cardiac form with no ventricular dysfunction	Cardiac form with ventricular dysfunction		
	A	B1	B2	C	D
Patients with findings compatible with acute Chagas disease	Patients at risk for developing CHF. They have positive serology, neither structural cardiopathy nor CHF symptoms. No digestive changes	Patients with structural cardiopathy, evidenced by electrocardiographic or echocardiographic changes, but with normal global ventricular function and neither current nor previous signs and symptoms of CHF	Patients with structural cardiopathy characterized by global ventricular dysfunction, and neither current nor previous signs and symptoms of CHF	Patients with ventricular dysfunction and current or previous symptoms of CHF (NYHA FC I, II, III or IV)	Patients with refractory symptoms of CHF at rest, despite optimized clinical treatment, requiring specialized interventions

Arq Bras Cardiol 2011; 97(2 supl.3): 1-48

El **resultado secundario** está determinado por el cambio de los niveles plasmáticos de los biomarcadores frente a los cambios en la carga parasitaria.

Medidas adicionales de valor científico: (a) el cambio en los niveles plasmáticos de los biomarcadores de inflamación y activación endotelial; (b) la incidencia y gravedad de los EA; (c) el cambio en la carga parasitaria y la respuesta serológica a lo largo del periodo de seguimiento, medido por PCR cuantitativa (qPCR); (d) los cambios en los niveles de los biomarcadores en

diferentes momentos del seguimiento y la correlación con la carga parasitaria y el régimen ATO; y (e) la incidencia de interrupción del tratamiento debido a EA graves.

9.2. Análisis estadístico: véase sección 11.
Los eventos clínicos relacionados con la EC ocurren con una incidencia baja en los pacientes con EC indeterminada debido a la patogénesis y la histéresis de la enfermedad, las características del parásito y los tiempos de respuesta del hospedero; por lo tanto, se necesitarían ensayos clínicos extensos para evaluar los cambios de incidencia. Además, los criterios de valoración serológica y clínica de curación requerirían de varios años a décadas en la EC crónica (Bern, 2015). En consecuencia, el criterio de curación como resultado primario, determinado por la seroreversión, no se considera en esta propuesta.

9.3. Descripción del estudio:
Se trata de un ensayo clínico de fase II de prueba de concepto con tres grupos diferentes: Dos grupos recibirán terapia antichagásica más 40 o 80 mg/día de ATO. El tercer grupo recibirá placebo más la terapia antichagásica. El placebo se justifica en tanto el paciente recibe el tratamiento antichagásico convencional completo y por lo tanto se asegura el manejo de su enfermedad de Chagas. En este ensayo clínico el placebo será un suplemento nutricional en comprimidos con aspecto similar al de ATO. Se eligió este producto dadas las eventuales dificultades para la fabricación de un número bajo de comprimidos, pues el ensayo no cuenta con un patrocinador de la industria farmacéutica capaz de fabricarlo bajo los estándares de las Buenas prácticas de Manufactura.

Los suplementos nutricionales no afectan los parámetros inflamatorios vasculares o cardiacos y por lo tanto, no introduce elementos de confusión en el análisis comparativo con atorvastatina.

Los pacientes recibirán primero la terapia antichagásica convencional durante dos meses y luego de un período de dos semanas (o hasta normalización de enzimas hepáticas) se iniciará el tratamiento con atorvastatina o placebo durante cuatro meses. Estas dosis se eligieron para minimizar los resultados falsos negativos, proporcionando así la mejor prueba de la hipótesis y maximizando el efecto farmacodinámico sobre la inflamación y la función cardíaca y endotelial. Además, la secuencia de los tratamientos está dirigida a minimizar las reacciones adversas que se podría originar por la eventual combinación de los fármacos antichagásicos con atorvastatina. Los sujetos del estudio serán aleatorizados, y los tres grupos serán doble ciego (tanto los investigadores clínicos como los sujetos serán ciegos) para ATO o placebo. Sin embargo, la terapia antichagásica será abierta. Además, el qPCR y las otras evaluaciones de laboratorio, así como los ECG y ecocardiogramas 2D se realizarán con los investigadores clínicos ciegos a la asignación de ATO o placebo.

Como se mencionó anteriormente, este estudio se llevará a cabo en cuatro centros: 1) Hospital San Juan de Dios y 2) Hospital Félix Bulnes en Santiago, Región Metropolitana, 3) Hospital Dr. Gustavo Fricke en Viña del Mar, 4) Hospital San Martín de Quillota, Región de Valparaíso. Cada centro admite un promedio de 60 nuevos pacientes en su Programa de Control de Chagas (PCC) cada año. Estos cuatro centros hacen parte de la red de atención del Programa Nacional de Control de la Enfermedad de Chagas.

9.4. Población de estudio:
Los pacientes mayores de 18 años y menores de 50 con EC crónica indeterminada que acudan al PCC serán inscritos de forma equitativa y aleatoria en los cuatro grupos del estudio. De acuerdo con las directrices del PCC, los pacientes mayores de 50 años no son elegibles para recibir terapia antichagásica. Para la selección de los sujetos, véase más abajo. La elección de esta población objetivo para la EC se debe principalmente a la necesidad médica no cubierta de un tratamiento nuevo, seguro y eficaz para la EC crónica indeterminada.

9.5. Duración del estudio y duración de la participación de los sujetos:
La duración total de la participación de los pacientes en el estudio será de doce meses, considerando dos semanas para el tamizaje y la evaluación según las directrices del PCC, ocho semanas de tratamiento con ATO, placebo y fármacos antichagásicos en sus respectivos grupos. La administración de ATO y placebo continuará durante un período adicional de ocho semanas. Habrá visitas de seguimiento hasta 12 meses después del inicio del tratamiento (véase en el anexo una presentación gráfica del diseño del estudio).

Tras el consentimiento informado voluntario y por escrito, los pacientes iniciarán una fase de evaluación de 14 días. Una vez que el paciente sea aleatorizado y se inicien los tratamientos, tendrá visitas de seguimiento durante la fase de tratamiento del estudio en los días 30, 60, 120 y 180, con un período de descanso de dos semanas o hasta normalización de niveles de enzimas hepáticas (ventana permitida de ± cuatro días), y tres visitas después del final del tratamiento (EOT) en los días 180, 240 y 360 (ventana permitida de ± 14 días). Además, se aconsejará a los pacientes que vuelvan en cualquier día durante el período de seguimiento si presentan alguna incidencia médica o EA.

Se espera que el reclutamiento se complete dentro de los 24 meses siguientes al inicio de este. Por lo tanto, el plazo entre el primer paciente que entra (FPI) y el último que sale (LPO) es de 36 meses. Sin embargo, la duración total del estudio se estima en 48 meses, desde la fase de inicio hasta el informe final del estudio.

9.6. Calendario de eventos para cada sujeto durante la fase de reclutamiento:

**Fase**	**Pre-Aleatorización**		**Aleatorización**					
**Período**	**Tamizaje**	**Línea de base**	**Tratamiento**				**Seguimiento**	
**Visita**	1	2	3	4	5	6	7	8
**Día(s)**	-14 to -1	0	30	60	120	180	240	360
**Procedimiento**								
Consentimiento Informado	x							
Aleatorización		x						
Ficha Clínica	x							
Fármacos previos/concurrentes	←---→							
Inclusión/Exclusión	x	x						

Signos Vitales	x	x	x	x	x	x	x	x
Examen físico completo	x				x			x
Radiografía de tórax	x				x			x
ECG	x			x	x	(x)[e]	(x)[e]	x
Ecocardiograma 2D	x							x
Prueba de Embarazo	x					x	x	
Serología	x							x
Laboratorio[a]	x	x	x	x	x	x	x	x
PCR cuantitativo	x				X[d]			x
Biomarcadores[b]		X		x	x			x
Examen Físico focalizado[c]	x	x	x	x	x	x	x	x
Eventos Adversos			←-------------------------------→					
Contabilidad de fármacos		x	x	x	x			

[a] Los parámetros de laboratorio incluirán: hemoglobina, recuento total de glóbulos blancos, recuento diferencial de glóbulos blancos y recuento de plaquetas. Los parámetros bioquímicos de laboratorio incluirán: CK, ALT, AST, GGT, fosfatasa alcalina, bilirrubina total y directa, un perfil lipídico: colesterol total, c-DHL y c-LDL, triglicéridos, glucemia en ayunas y creatinina;

[b] Biomarcadores: BNP, cTnT, IFN-γ, IL-1β, IL-4, IL-17A, e IL-10, sICAM-1, sVCAM-1, sE-selectina;

[c] Examen físico centrado sólo en la evaluación de eventos adversos;

[d] Las pruebas de PCR en estos puntos temporales se harán con una sola muestra de 10 ml;

[e]los ECG se realizarán en estas visitas sólo en caso de anomalías previamente identificadas

9.7. Evaluaciones:

Al ingresar al PCC, los pacientes serán sometidos a una historia clínica completa con énfasis en la EC; datos demográficos e historial de medicamentos; examen físico, peso y estatura corporal, signos vitales y temperatura corporal.

Se recogerán 10 ml de sangre, en tubos separados, para realizar evaluaciones hematológicas y bioquímicas: hemoglobina, recuento total y diferencial de glóbulos blancos, recuento de plaquetas, CK, ALT, AST, GGT, fosfatasa alcalina, bilirrubina total y directa, un perfil lipídico: colesterol total, c-DHL y c-LDL, triglicéridos, glucemia en ayunas y creatinina. También se realizará una prueba de embarazo en suero. Esta prueba se realizará en los laboratorios clínicos de cada centro.

Se recogerá una muestra de sangre de 3 mL para la serología convencional de la EC que se realizará en los laboratorios clínicos de cada centro, y para una prueba serológica confirmatoria que debe realizarse en el ISPCH o un laboratorio acreditado para tal efecto.

Se recogerán 5 mL de sangre para realizar el qPCR. Para el desarrollo clínico y la prueba de concepto, se ha propuesto utilizar las pruebas parasitológicas de PCR cuantitativa como marcadores de eficacia en los estudios clínicos de EC crónica indeterminada (Parrado et al., 2019). Así, en este ensayo se utilizaría la técnica de PCR en tiempo real descrita por Duffy et al. (Duffy et al., 2013). Las muestras de sangre se añadirán inmediatamente a un tubo que contenga un volumen (10mL) de una solución de tampón de guanidina/ClH 6M EDTA 0,2M pH 8,0 (GEB) (Schijman et al., 2003). Las muestras con tampón de guanidina pueden permanecer a temperatura ambiente durante un máximo de 30 días. Para periodos más largos, es necesario almacenarlas en un refrigerador. Tras la extracción de ADN, con un kit comercial, las muestras se procesarán en un sistema de RT-PCR Applied 155 Biosystems 7300 (Thermo Fisher).

Se extraerán 3 ml de sangre adicionales para los biomarcadores: BNP, cTnT, IFN-γ, IL-1β, IL-4, IL-17A, e IL-10, sICAM-1, sVCAM-1, sE-selectina. Estos marcadores se seleccionaron siguiendo la revisión de la literatura (Cortes-Serra et al., 2020; Echeverría et al., 2020; Llaguno et al., 2019). Los niveles séricos de estos marcadores se determinarán mediante un enfoque multiplex con un Luminex 200 (R&Dsystems). Para establecer la reproducibilidad biológica se realizará un estudio piloto previo a los análisis de las muestras de este ensayo.

Por último, se realizará un ECG de referencia en reposo y un ecocardiograma 2D. Ambos exámenes se realizarán en cada centro. El ECG debe ser normal o con cambios inespecíficos (bloqueo de rama derecha incompleto, bloqueo fascicular anterior izquierdo incompleto, bradicardia leve, aumento menor del intervalo PR y cambios menores del ST-T). Cualquier anormalidad clínicamente significativa encontrada en el electrocardiograma conducirá automáticamente a la exclusión del paciente de este estudio (ver criterios de exclusión).

Después de la aleatorización y antes de iniciar los tratamientos, los pacientes serán sometidos a un nuevo examen físico focalizado, y se les extraerá sangre para el qPCR y la determinación de biomarcadores.

9.8. Diagrama general del diseño del estudio:

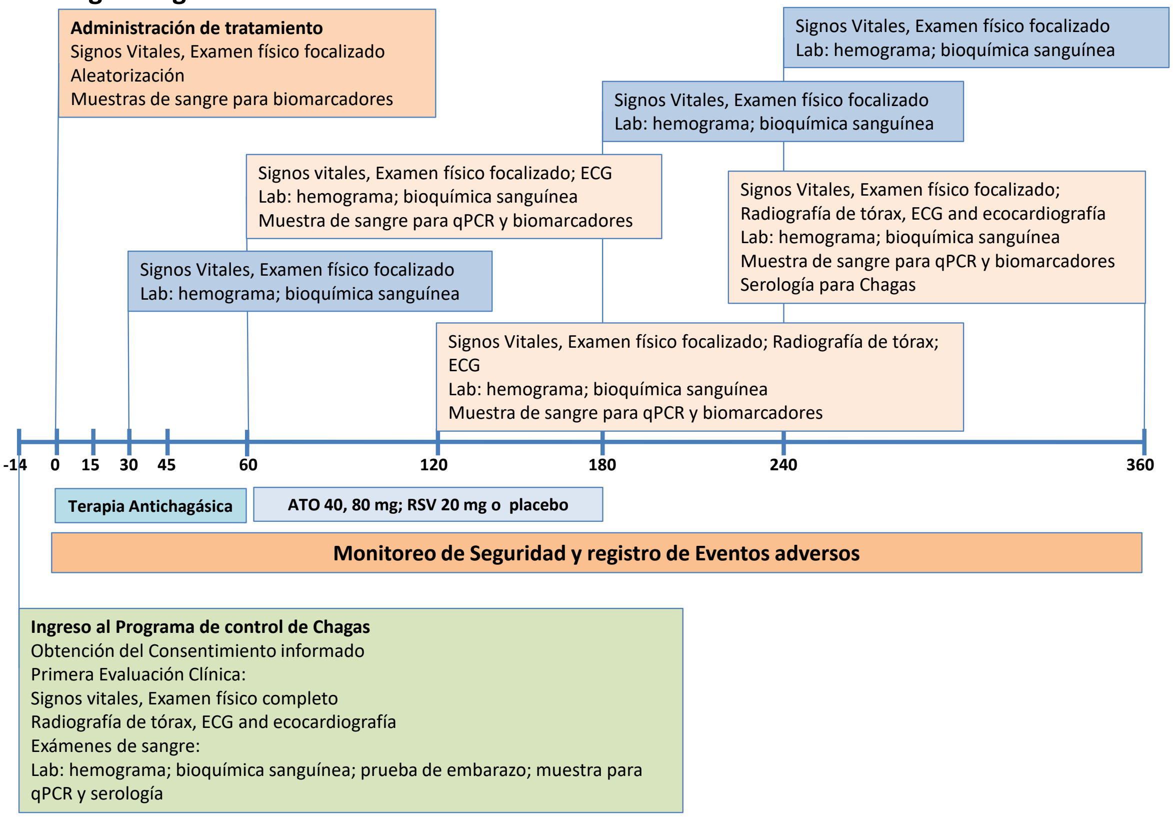

10.SELECCIÓN Y RETIRADA DE SUJETOS

10.1. Criterios de inclusión:

Los sujetos que serán elegibles para ser inscritos en este ensayo deben ser adultos mayores de 18 años y menores de 50, con un peso superior a 40 kg, con una serología confirmatoria convencional para la infección por T. cruzi del Instituto de Salud Pública de Chile (ISPCH), y un qPCR positivo serán seleccionados para participar en este ensayo. Solo se incluirá pacientes con diagnóstico nuevo de enfermedad de Chagas en la fase crónica indeterminada. Además, deben tener valores normales en las pruebas de laboratorio para los siguientes parámetros: recuento total de glóbulos blancos, recuento de plaquetas, creatina kinasa (CK), alanina aminotransferasa (ALT), aspartato aminotransferasa (AST), bilirrubina total o creatinina, o una gamma-glutamil transferasa (GGT) <= 2 veces el límite superior de la normalidad (X ULN); Las mujeres en edad reproductiva deben tener una prueba de embarazo en suero negativa, no deben estar amamantando y deben utilizar sistemáticamente un método anticonceptivo altamente eficaz durante toda la fase de tratamiento.

Los sujetos deben cumplir también todos los siguientes criterios: capacidad de cumplir con todas las pruebas y visitas de seguimiento especificadas en el protocolo y tener una dirección permanente; formulario de consentimiento informado firmado y por escrito.

10.2. Criterios de exclusión:

signos y síntomas de la forma digestiva de la EC; EC cardiaca crónica en estadio II o superior; condiciones de salud agudas o crónicas como infecciones agudas, antecedentes de infección por VIH, diabetes, enfermedades hepáticas y renales; enfermedad cardiaca preexistente no relacionada con la enfermedad de Chagas; hipotiroidismo, e historia familiar de alteraciones musculares; contraindicación formal para recibir NFX o BZD, antecedentes conocidos de hipersensibilidad, alergia o reacciones adversas graves a ATO, BZD o NFX; antecedentes de tratamiento previo para la EC; antecedentes de tratamiento previo con atorvastatina, lovastatina, rosuvastatina, simvastatina o cualquier otra estatina; cualquier uso concomitante de agentes antimicrobianos; antecedentes de abuso de alcohol o drogas; cualquier condición que impida la medicación oral; uso concomitante o previsto de modificadores del CYP3A4; antecedentes médicos de síndrome de QT corto familiar o terapia concomitante con medicamentos que puedan acortar el intervalo QT. Valores anormales en las pruebas de laboratorio para los siguientes parámetros: recuento total de glóbulos blancos, recuento de plaquetas, creatina kinasa (CK), alanina aminotransferasa (ALT), aspartato aminotransferasa (AST), bilirrubina total o creatinina, o una gamma-glutamil transferasa (GGT) > 2 veces el límite superior de la normalidad (X ULN); Las mujeres embarazadas o en período de lactancia; negarse a usar un método anticonceptivo altamente eficaz durante la fase de tratamiento.

Los pacientes que cumplan los criterios de selección serán sometidos al procedimiento de aleatorización. Sólo los sujetos inscritos en este ensayo recibirán los medicamentos del estudio. A los pacientes se les asignará, en orden ascendente, un número de identificación del ensayo (NIT) según el orden de reclutamiento. Un paciente se considera aleatorizado cuando recibe un NIT.

11.TRATAMIENTO DE LOS SUJETOS

11.1.1. Dosis y regímenes de tratamiento

Se adquirirán comprimidos de 40 y 80 mg de ATO en un laboratorio farmacéutico con registro sanitario vigente reconocido por el ISPCH y que ofrezca el principio activo en una formulación bioequivalente. Los comprimidos del placebo corresponderán a comprimidos de un complemento nutricional provistos por un distribuidor con registro sanitario vigente.

La adquisición se realizará a través de la Farmacia del Hospital Clínico de la Universidad de Chile.

Los comprimidos de ATO y placebo se re-envasarán en frascos negros de plástico de 200 ml de capacidad, previamente esterilizados con luz UV, bajo condiciones asépticas usando una campana de bioseguridad tipo II. Los frascos contendrán 120 comprimidos cada uno y todos provenientes del mismo lote para permitir la trazabilidad del medicamento.

La rotulación de cada frasco se realizará según lo requerido por las buenas prácticas de manufactura incluyendo: a) nombre y datos de contacto (teléfono y correo electrónico) del investigador principal; b) Forma farmacéutica, vía de administración, número de unidades; c) Número de lote y código (asignado por el investigador principal) del laboratorio proveedor; d) Código del proyecto; e) instrucciones de uso; g) código de aleatorización; f) La advertencia: “para uso exclusivo en estudio clínico”; h) fecha de vencimiento; i) La frase: "Manténgase fuera del alcance de los niños".

Los fármacos antichagásicos NFX o BZD serán suministrados por el Ministerio de Salud de Chile, como parte del PCC. El tratamiento antichagásico se administrará según las indicaciones del "Manual de Procedimientos para la Atención de Pacientes con Chagas", (MINSAL, 2017) y la administración de ATO, fármacos antichagásicos y tratamiento placebo se supervisará en cada una de las visitas de los pacientes para determinar la contabilidad de los comprimidos y el cumplimiento con los fármacos. La dosis de BZD será de 5 mg/kg/día cada 12 horas (BID), y la de NFX de 5-10 mg/kg/día BID. Las dosis de ATO serán de 40 y 80 mg/día para cada grupo respectivo, y se administrarán por la mañana.

En cada visita, el paciente deberá llevar todos los medicamentos restantes del estudio en las visitas de los días 30, 60, 120 y 180 para comprobar el cumplimiento del tratamiento antichagásico y experimental prescritos, efectuar la contabilidad de los medicamentos (Torrico et al., 2018). **Si un sujeto desarrolla síntomas o signos atribuibles a la intolerancia a NFX, puede ser cambiado a BZD sin ser retirado del estudio**.

El ATO, el placebo y los fármacos antichagásicos que se proporcionen no deben utilizarse para otros fines que no sean los de este ensayo.

Es necesario indicar en este punto que si durante las visitas de seguimiento, el paciente manifiesta sintomatología sugerente de insuficiencia cardíaca, será remitido a consulta cardiológica para evaluación. La adición de terapia específica para el manejo de falla cardiaca se consignará en la ficha clínica del ensayo.

11.1.2. Fármacos prohibidos antes de administrar los tratamientos del estudio y durante el estudio.

No se permite el uso concurrente de terapia sistémica antimicótica o de otros fármacos en experimentación (nuevas entidades químicas o biológicas). En caso de que el sujeto se encuentre en tratamiento sistémico antimicótico, solo podrá incluirse en el presente estudio después de un período de espera de 30 días. En el caso de los inhibidores de la bomba de protones, el período de espera deberá ser de 10 días. Se permite el uso de antagonistas H_2. El uso concomitante de fármacos que modifican los niveles de estatinas como carbamazepina, eritromicina, claritromicina, colchicina, rifampicina, hierba de san Juan (*Hypericum perforatum*), así como inmunosupresores o dosis suprafisiológicas de glucocorticoides (excepto para el tratamiento a corto plazo del asma, enfermedad pulmonar obstructiva crónica (EPOC), Hiperreactividad de las vías aéreas o reacciones de hipersensibilidad o erupciones alérgicas) tampoco está permitido.

11.2. Eventos adversos y retiro del estudio

11.2.1. Eventos adversos:

Un EA se definirá como cualquier acontecimiento médico adverso (cualquier síntoma, signo o enfermedad desfavorable e involuntario, incluyendo un hallazgo anormal de laboratorio o ECG) o el empeoramiento de cualquier condición preexistente, que ocurra durante el estudio, se considere o no relacionado causalmente con el estudio o con los medicamentos del estudio. Los resultados anormales de laboratorio (hematología y bioquímica) se notificarán como EA si: a) se producen o empeoran después del inicio de los tratamientos del estudio, b) son considerados un cambio adverso clínicamente significativo por el investigador clínico, o c) son superiores a los

Criterios Terminológicos Comunes para EA (CTCEA) de grado 1 (Instituto Nacional del Cáncer (EE.UU.), 2017), a menos que estén asociados a un EA clínico ya notificado.

Los investigadores clínicos, o el personal adecuado del centro (no implicado en el estudio), examinarán a cualquier sujeto que experimente un EA tan pronto como sea posible. El investigador hará lo que sea médicamente necesario para la seguridad y el bienestar del sujeto. El sujeto permanecerá en observación mientras reciba cualquier fármaco del ensayo, y durante los dos meses siguientes al último día de administración del fármaco, o durante más tiempo si está médicamente indicado en opinión del investigador. Todos los EA observados o notificados tras la administración de los tratamientos en investigación serán objeto de seguimiento hasta que se resuelvan o hasta que estén médicamente estables.

Para este estudio, los investigadores clínicos de cada centro informarán al investigador principal, quien, a su vez, será responsable de informar de los EA a los Comités Éticos de los Centros y al Comité de ética de la Investigación en Seres Humanos de la Facultad de Medicina de la Universidad de Chile y a la sección de estudios clínicos del ISPCH. Los investigadores clínicos deberán informar de todos los EA observados directamente y de todos los comunicados espontáneamente por los sujetos, utilizando una terminología médica concisa. Además, durante cada visita al ensayo, los sujetos serán entrevistados y sometidos a un examen físico específico para la evaluación de los EA. Todo evento adverso deberá ser registrado en la ficha clínica del sujeto y en una base *ad hoc* para tal efecto.

11.2.2. Evento adverso grave (EAG):

Un evento adverso se definirá como grave si es

- fatal
- pone en peligro la vida del paciente
- requiere o prolonga la hospitalización
- da lugar a una discapacidad persistente o significativa
- es una anomalía congénita/defecto de nacimiento
- da lugar a un evento médico importante que puede no poner en peligro la vida de forma inmediata o que no da lugar directamente a la muerte o a la hospitalización, pero que puede poner en peligro la seguridad del paciente o puede requerir una intervención para evitar los otros resultados enumerados anteriormente.

Los eventos graves también incluyen cualquier otro evento que se defina como grave para los fines específicos del protocolo o que sea definido como grave por el ISPCH.

Cada evento adverso debe ser clasificado por el investigador clínico como grave o no grave. Esta clasificación determinará el procedimiento de notificación del evento.

Todos los EAG deben ser reportados inmediatamente (dentro de las 24 horas de conocimiento del EAG por parte del investigador clínico) al Investigador Principal, utilizando el formulario de reporte de EAG. Esto incluye una descripción del evento, la fecha de inicio y el tipo, la duración, la gravedad, la relación con el medicamento del estudio, el resultado, las medidas tomadas y todos los demás datos clínicos y de laboratorio pertinentes. El informe inicial debe ir seguido de la presentación de información adicional (formulario de seguimiento de los EAG) a medida que esté

disponible. Los informes de seguimiento deben presentarse lo antes posible y, a ser posible, en un plazo de 5 días laborables.

Los EAG también deben notificarse en el formulario de notificación de evento adversos del ensayo clínico. Debe tenerse en cuenta que el formulario para la notificación de EAG no es el mismo que la sección de eventos adversos del Formulario de Registro Individual (CRF, siglas en inglés). Aunque se recogen los mismos datos, los dos formularios deben completarse de manera coherente, y debe utilizarse la misma terminología médica.

Los eventos adversos no graves deben notificarse en el CRF, que debe enviarse al investigador Principal como se especifica en la sección 14.1 de este protocolo.

Además de notificar inmediatamente los EAG al Investigador Principal, los investigadores son responsables de notificar los EAG que se produzcan en su centro a su Comité de Ética Clínica correspondiente, así como cualquier informe de seguridad periódico, siguiendo los requisitos locales del centro. Para los fines de este ensayo, el investigador clínico de cada centro y el Investigador Principal asumirán la responsabilidad de notificar los EAG al ISPCH y a los comités éticos, según sea necesario.

11.2.3. Calificación de la gravedad de los eventos adversos

La gravedad es una determinación clínica de la intensidad de un EA. La gravedad de un EA debe calificarse utilizando los CTCEA del Instituto Nacional del Cáncer (Instituto Nacional del Cáncer (EE.UU.) 2017). En caso de EA que no estén descritos en el CTCAE, el investigador utilizará la terminología LEVE, MODERADA o SEVERA para describir la gravedad máxima del acontecimiento adverso de la siguiente manera:

LEVE: No interfiere con las funciones habituales del sujeto; MODERADO: Interfiere hasta cierto punto con las funciones habituales del sujeto; SEVERO: Interfiere significativamente con las funciones habituales del sujeto

Esta información sobre la clasificación de los EA se introducirá en la sección de eventos adversos del CRF.

Hay que tener en cuenta la distinción entre severidad y gravedad de los acontecimientos adversos. Un acontecimiento adverso grave no es necesariamente un acontecimiento serio.

11.2.4. Evaluación de la causalidad de los eventos adversos

Tanto para los eventos adversos graves como para los no graves, el investigador debe evaluar la relación potencial entre el evento adverso y el medicamento del estudio, es decir, determinar si existe una posibilidad razonable de que el medicamento del estudio haya causado o contribuido al acontecimiento adverso. Para facilitar la toma de decisiones en la evaluación de la causalidad, se debe considerar lo siguiente antes de tomar una decisión:

- Historia clínica
- Falta de eficacia/empeoramiento de la enfermedad existente
- Medicamentos del estudio
- Otros medicamentos (concomitantes o anteriores)

- Retiro del fármaco del estudio, especialmente tras la interrupción del ensayo o tras la finalización de la administración del fármaco durante el estudio
- Tratamiento erróneo con la medicación del estudio (o concomitante)
- Procedimiento relacionado con el protocolo

La relación de un EA con el tratamiento en investigación es evaluada y determinada por el investigador después de una cuidadosa consideración del evento en términos de plausibilidad biológica, posibles causas no relacionadas, cualquier condición médica preexistente o medicación concomitante, relación temporal entre la administración del tratamiento en investigación y el inicio (o empeoramiento) del evento, y patrones conocidos de respuesta a estatinas en general.

La evaluación de la relación se basa en las siguientes directrices:

No relacionado: No hay relación temporal con el producto en investigación o de control o hay una explicación alternativa plausible.

Relacionado: Todos los EA se consideran relacionados si no se juzgan como no relacionados y/o no hay una etiología alternativa convincente.

La decisión de suspender y reanudar el tratamiento o de interrumpirlo permanentemente debido a un acontecimiento adverso se dejará en manos del investigador clínico correspondiente.

11.2.5. Retiro del estudio:

Se considerará que los pacientes se han retirado del estudio si han entrado en él (es decir, han dado su consentimiento informado y han recibido al menos una dosis de tratamiento) pero no han completado la fase de tratamiento del estudio ni las evaluaciones de seguimiento después del EOT.

Las siguientes razones pueden ser consideradas para indicar el retiro del paciente del estudio: reacciones cutáneas graves o reacciones cutáneas moderadas repetidas; ALT sérica superior a 3 X ULN en cualquier momento o elevación de la bilirrubina sérica >2 X ULN; debilidad muscular y aumento de CK > 1ULN; EAG o severo; cualquier condición que el investigador clínico o cualquier otro médico de cabecera no involucrado en el estudio considere médicamente necesaria para interrumpir el tratamiento y retirar a un paciente del estudio; desviación significativa del protocolo; pérdida de seguimiento; retractación del consentimiento informado por parte del paciente; finalización del estudio por parte del investigador. Si un sujeto no regresa a una visita programada, se debe hacer todo lo posible para contactar con él. En cualquier circunstancia, se debe hacer todo lo posible para documentar el resultado del sujeto, si es posible. Si el sujeto retira su consentimiento, no se deben realizar más evaluaciones y no se debe intentar recolectar datos adicionales, con la excepción de los datos de seguridad, que deben ser recolectados si es posible. Los datos obtenidos de los pacientes retirados antes de su retiro seguirán siendo considerados. Los sujetos retirados de este estudio no serán reemplazados.

La interrupción del tratamiento no implica el retiro del estudio. En estos casos, el tratamiento puede interrumpirse durante unos días; por lo tanto, el tratamiento se considerará incompleto o retrasado. Se puede reanudar el tratamiento con ATO y con fármacos antichagásicos, según la

valoración del investigador clínico a cargo del paciente. Estos pacientes deben continuar con las visitas y evaluaciones del estudio según lo previsto.

Los sujetos retirados del estudio no serán reemplazados.

11.2.6. Procedimiento de apertura del ciego

La apertura del ciego se realizará sólo en el raro caso de una emergencia médica o de EAG, EA severos e inesperados, cuando el médico a cargo del paciente (y no el investigador responsable de este proyecto) considere que el paciente no puede ser tratado adecuadamente a menos que se conozca la asignación del tratamiento. Deberá levantarse un informe adecuado de apertura de ciego junto con el llenado del formulario de notificación de EA, tal y como proporciona el sistema de farmacovigilancia del ISPCH.

Los pacientes pueden recibir un tratamiento concomitante para las incidencias médicas durante el estudio. Todo el tratamiento concomitante tomado por el paciente durante el estudio, desde la fecha de la firma del consentimiento informado hasta la última visita de seguimiento, se registrará en la sección apropiada del formulario de registro de Individual (CRF, siglas en inglés).

12.Análisis de datos y métodos estadísticos:

12.1. Tamaño de la muestra:

Un tamaño muestral de 75 pacientes por grupo es suficiente para alcanzar una potencia del 90% a un nivel de significación global del 5% (a dos bandas), suponiendo que la proporción de pacientes que mejoran los parámetros electrocardiográficos y de ecocardiografía, así como los niveles de los marcadores de función cardíaca BNP y cTnT en el EOT en el grupo de tratamiento experimental es de 0,40, y la proporción en el grupo de placebo es de 0,20 con una razón de probabilidad (*odds ratio*) de 2,5. Considerando una tasa de abandono estimada del 25%, se reclutarían un total de 100 pacientes por grupo (300 pacientes en total) (Fleiss et al., 2003). El estudio tendrá la potencia suficiente para proporcionar pruebas de la eficacia superior de cualquiera de las dosis de estatina más el tratamiento antichagásico en relación con el tratamiento antichagásico solo.

12.2. Aleatorización y asignación del tratamiento:

Se preparará una lista de aleatorización generada por computador, estratificada por centro utilizando un tamaño de bloque de doce. Cada centro recibirá una lista de números de aleatorización y los correspondientes paquetes de ATO o placebo. Tras la inscripción del paciente, se asignará el siguiente número de aleatorización disponible en el centro (por orden cronológico) y se entregará el paquete de tratamiento correspondiente.

12.3. Poblaciones a analizar:

Se incluirán tres poblaciones en el análisis: i) la población por intención de tratar (ITT) que comprende todos los pacientes aleatorizados por sus grupos de tratamiento asignados, ii) la población por protocolo compuesta por todos los pacientes ITT sin ninguna desviación significativa del protocolo, y iii) la población de seguridad que comprende todos los pacientes aleatorizados y que han recibido al menos una dosis del tratamiento del estudio.

Para los datos demográficos y las características basales, se presentarán estadísticas descriptivas tanto de la población por protocolo como de la población ITT.

12.4. Análisis de eficacia:

Para el análisis del resultado primario, se utilizará una prueba exacta de Fisher unilateral de la proporción de pacientes con una disminución significativa de los niveles de biomarcadores en las poblaciones ITT (análisis primario), y por protocolo (análisis secundario). Se realizará la prueba ANOVA para analizar las diferencias de las varianzas entre los distintos marcadores. Para todas las comparaciones secundarias de proporciones entre ATO frente a placebo, incluyendo la respuesta parasitológica al tratamiento, se realizará una prueba exacta. Se utilizarán análisis de clases latentes y multivariantes para evaluar la asociación entre la respuesta parasitológica, los cambios en los biomarcadores y las dosis de ATO.

12.5. Análisis de seguridad:

Se describirá la proporción de pacientes que presentan al menos un EA. Se presentará la tasa de incidencia y el intervalo de confianza del 95% por grupo del estudio. Por lo demás, sólo se presentarán estadísticas descriptivas. Los parámetros de seguridad de laboratorio (hematología y bioquímica) también se describirán individualmente por cada grupo del estudio, mostrando la proporción de pacientes por grado de elevación en relación con el ULN y con los valores iniciales, y los cambios de los niveles sanguíneos a lo largo del tiempo.

12.6. Criterios Término anticipado del estudio

El investigador principal se reserva el derecho de terminar el estudio en cualquier momento antes de la inclusión del número previsto de sujetos, por razones científicas o administrativas válidas. Las razones para la terminación del estudio pueden incluir, pero no limitarse a, las siguientes:

- La tasa de enrolamiento es extremadamente baja o falta de pacientes elegibles
- Alta frecuencia de violaciones del protocolo
- Datos inexactos o incompletos
- Prácticas inseguras o poco éticas
- Siguiendo la recomendación de los comités de ética clínica

En el caso de que un estudio sea terminado anticipadamente, el investigador clínico tiene que

- Completar todos los CRF en la mayor medida posible y devolver todos los implementos, CRF y materiales de estudio relacionados que se le proporcionó.
- Responder a todas las preguntas del investigador principal relacionadas con los datos de los sujetos inscritos en el centro antes de la finalización del estudio.
- Asegurarse de que los sujetos inscritos en el estudio que aún no hayan alcanzado un punto de seguimiento reciban la atención médica necesaria

El investigador deberá proporcionar por escrito las razones de su decisión a la autoridad sanitaria nacional y al patrocinador

13. Ética:

El protocolo experimental de este estudio se ha diseñado de acuerdo con los principios éticos generales recogidos en la Declaración de Helsinki (2013).

Los protocolos del ensayo (y sus enmiendas y correcciones) han sido aprobados por el Comité de Ética de la Investigación en Seres Humanos de la Facultad de Medicina de la Universidad de Chile, del Comité de Ética Clínica del Hospital San Juan de Dios y del Hospital Félix Bulnes y aprobados por los directores de los Hospitales Gustavo Fricke de Viña del Mar y San Martin de Quillota, de

acuerdo con la ley N° 20120, y su respectivo reglamento. Cada paciente dará su consentimiento informado, para lo cual se dejará constancia en el formulario de consentimiento

14. Proceso de consentimiento informado

La inclusión en el estudio sólo se producirá si el sujeto da su consentimiento informado por escrito. Es responsabilidad del investigador obtener el consentimiento informado voluntario y por escrito de cada individuo que participe en este estudio, tras la presentación adecuada de los objetivos, métodos, beneficios previstos y posibles peligros del estudio. El paciente tendrá tiempo para discutir la información recibida con miembros de la comunidad o de la familia antes de decidir su consentimiento. Se pedirá al sujeto que dé su consentimiento por escrito y firmado.

Si el sujeto es analfabeto, incluirá una marca en el formulario y deberá firmar un testigo alfabetizado (esta persona no debe tener ninguna relación con el equipo de investigación y, si es posible, debe ser seleccionada por el participante).

Si nueva información de seguridad da lugar a cambios significativos en la evaluación de riesgos/beneficios, el formulario de consentimiento debe ser revisado y actualizado si es necesario. Todos los sujetos (incluidos los que ya están siendo tratados) deben ser informados de la nueva información, recibir una copia del formulario revisado y dar su consentimiento para continuar en el estudio.

14.1. Costes de los pacientes:

Los pacientes podrán ser reembolsados por el viaje de ida y vuelta al lugar del estudio, pero no recibirán ningún pago por la participación en el ensayo.

15. Acceso directo a los datos y documentos fuente:

El investigador principal permitirá el acceso directo a los datos o documentos fuente para la realización de la monitorización, la auditoria, la revisión por los Comités de Ética, así como la inspección del ensayo por las autoridades sanitarias.

16. Control y garantía de calidad

El investigador mantendrá registros adecuados y precisos que documenten completamente la realización del estudio y permitan verificar posteriormente los datos de este. Estos documentos incluyen el Manual del Investigador, el Protocolo del estudio y sus modificaciones, el formulario de consentimiento informado, el CRF y las aprobaciones por parte de los comités de ética y autoridades sanitarias. Además contendrá los registros de contabilidad de medicamentos, los CV de los coinvestigadores y otros documentos/correspondencia apropiados.

16.1. Formularios de Registro Individual (CRF)

Los datos de cada participante en el estudio serán recolectados por el investigador clínico responsable (ICR). La información específica del estudio será ingresada en un *Formulario de Registro Individual* (CRF, siglas en inglés) electrónico, ambientado en la plataforma RedCap de la Facultad de Medicina de la Universidad de Chile, la cual es una plataforma electrónica cifrada diseñada para el resguardo seguro de datos de investigación clínica y biomédica.

Todos los datos ingresados al CRF serán anónimos e identificados exclusivamente por el numero único del paciente (NUP), el cual se asigna de manera consecutiva para cada centro en la medida

que van siendo reclutados los participantes y que es independiente del código de aleatorización del tratamiento asignado. Este NUP es proporcionado por el Investigador Principal (IP) del estudio a solicitud del ICR en cada centro en el momento de reclutar al participante y después de la firma del Formulario de Consentimiento Informado. En la plataforma RedCap, el NUP será reemplazado por un código generado aleatoriamente, rompiendo el vínculo entre el NUP y la plataforma. De la misma manera, el centro y el investigador clínico recibirán un código único, generado electrónicamente, de manera que el código único del paciente no sea asociado al nombre del investigador clínico o del centro de estudio.

El investigador Principal del proyecto no recopilará información personal de los sujetos del estudio en el FRC. Se entiende por información personal el nombre (incluidas sus iniciales), dirección de residencia incluyendo la comuna en la que vive y solo se registrará la Región de procedencia, Tampoco se hará registro del número de teléfono de contacto, fecha exacta de nacimiento, o cualquier otra información que conduzca a la identificación del sujeto. Tampoco se registrarán datos como ingreso familiar, ocupación o nivel educativo, por no ser variables atingentes a este estudio. No obstante, el investigador clínico debe mantener la información de contacto de cada participante, para que todos puedan ser contactados rápidamente por el investigador clínico, si es necesario.

El investigador clínico del proyecto debe asegurar la exactitud, integridad, legibilidad y el llenado oportuno de todos los datos comunicados al IP en los CRF y cualquier otra información adicional que se requiera. El IP debe mantener los documentos fuente (como la ficha clínica del estudio, los informes de laboratorio y de interconsulta), para su posible revisión y/o auditoría por parte de los Comités de Ética Científica o las Autoridades Reguladoras. El IP es el responsable de mantener todos los formularios de consentimiento, los CRF y la lista de NUP de los sujetos en un lugar seguro. Los documentos en formato físico serán guardados bajo llave en dependencias del programa de Farmacología Molecular y Clínica de la Facultad de Medicina de la Universidad de Chile, con acceso restringido a personal autorizado exclusivamente.

16.2. Documentos fuente

La verificación de los datos del CRF debe realizarse mediante la inspección directa de los documentos fuente. Los documentos fuente incluyen la ficha clínica del sujeto, las notas del Investigador clínico, los informes de laboratorio, informes de radiología, ECG, y ecocardiografía, informes de evaluación especiales, los formularios de consentimiento informado firmados, y los registros de selección e inscripción de los sujetos. Los registros y en general, toda la documentación relacionada con el estudio será resguardada durante cinco años después de la fecha del LPO o de acuerdo con la regulación vigente al momento de la aprobación del presente protocolo por las autoridades sanitarias.

El investigador debe mantener los documentos fuente (como los informes de laboratorio y de consulta, los informes de historia y examen físico), para una posible revisión y/o auditoría por parte de los Comités de Ética o por las Autoridades Reguladoras. El Investigador clínico registrará la fecha de la visita de cada sujeto junto con un resumen de su estado y progreso en el estudio.

17. Manejo de los datos y archivo de los registros

Debe completarse un CRF para todos los pacientes que hayan dado su consentimiento informado. El presente ensayo clínico utilizará un CRF electrónico alojado en la plataforma RedCap de la Facultad de Medicina de la Universidad de Chile, que además, permite el almacenamiento de los datos del estudio manteniendo la confidencialidad de acuerdo con la legislación nacional vigente sobre datos de los pacientes.

Todas las entradas en el CRF son responsabilidad del investigador o de un miembro cualificado del personal designado. El investigador certificará por escrito al comienzo del ensayo que su firma electrónica es el equivalente legalmente vinculante de una firma escrita.

Los datos serán revisados continuamente por un monitor clínico designado para tal efecto. Las consultas de datos se generarán, documentarán y resolverán de forma continua durante el ensayo.

Los investigadores deben asegurar que se mantendrá el anonimato de los sujetos y que sus identidades están protegidas de partes no autorizadas. En los CRF u otros documentos, los sujetos no deben ser identificados por sus nombres, sino exclusivamente por un código de identificación. El investigador principal debe mantener una lista de inscripción de sujetos que muestre los códigos, nombres y direcciones. El investigador principal deberá mantener en estricta confidencialidad los documentos que se presenten para auditoria y los formularios de consentimiento escrito firmados por el sujeto.

18. Informes y publicación

Todos los ensayos clínicos se registrarán en un registro de ensayos clínicos reconocido, como www.clinicaltrials.gov.

Los resultados de este estudio pueden ser publicados o presentados en reuniones científicas.

De acuerdo con la práctica editorial y ética habitual, la publicación de los ensayos multicéntricos sólo en su totalidad y no como datos de centros individuales.

19. Bibliografía

Acevedo, G.R., M.C. Girard, and K.A. Gomez. 2018. The Unsolved Jigsaw Puzzle of the Immune Response in Chagas Disease. Front Immunol. 9:1929.

Alonso-Padilla, J., M. Gallego, A.G. Schijman, and J. Gascon. 2017. Molecular diagnostics for Chagas disease: up to date and novel methodologies. Expert Rev Mol Diagn. 17:699-710.

Andrade JP, Marin-Neto JA, Paola AA, Vilas-Boas F, Oliveira GM, Bacal F, Bocchi EA, Almeida DR, Fragata Filho AA, Moreira M da C, Xavier SS, Oliveira Junior WA, Dias JC. 2011. Sociedade Brasileira de Cardiologia. I Diretriz Latino Americana para o Diagnóstico e Tratamento da Cardiopatia Chagásica. Arq bras Cardiol. 97(2 Suppl 3):1-48.

Apt, W., X. Aguilera, A. Arribada, C. Perez, C. Miranda, G. Sanchez, I. Zulantay, P. Cortes, J. Rodriguez, and D. Juri. 1998. Treatment of chronic Chagas' disease with itraconazole and allopurinol. Am J Trop Med Hyg. 59:133-138.

Apt, W., A. Arribada, I. Zulantay, J. Rodriguez, M. Saavedra, and A. Munoz. 2013. Treatment of Chagas' disease with itraconazole: electrocardiographic and parasitological conditions after 20 years of follow-up. J Antimicrob Chemother. 68:2164-2169.

Barizon, G.C., M.V. Simoes, A. Schmidt, L.P. Gadioli, and L.O. Murta Junior. 2020. Relationship between microvascular changes, autonomic denervation, and myocardial fibrosis in Chagas cardiomyopathy: Evaluation by MRI and SPECT imaging. J Nucl Cardiol. 27:434-444.

Bern, C. 2015. Chagas' Disease. N Engl J Med. 373:456-466.

Bestetti, R.B., and C.B. Restini. 2014. Precordial chest pain in patients with chronic Chagas disease. Int J Cardiol. 176:309-314.

Booney, K.M., D.J. Luthringer, S.A. Kim, N.J. Garg, and D.M. Engman. 2019. Pathology and Pathogenesis of Chagas Disease Heart. Annu. Rev. Pathol. Mech. Dis. 14:421-447.

Borges, J.P., F. Mendes, G.O. Lopes, A.S. Sousa, M.F.F. Mediano, and E. Tibirica. 2018. Is endothelial microvascular function equally impaired among patients with chronic Chagas and ischemic cardiomyopathy? Int J Cardiol. 265:35-37.

Botoni, F.A., P.A. Poole-Wilson, A.L. Ribeiro, D.O. Okonko, B.M. Oliveira, A.S. Pinto, M.M. Teixeira, A.L. Teixeira, Jr., A.M. Reis, J.B. Dantas, C.S. Ferreira, W.C. Tavares, Jr., and M.O. Rocha. 2007. A randomized trial of carvedilol after renin-angiotensin system inhibition in chronic Chagas cardiomyopathy. Am Heart J. 153:544 e541-548.

Brener, Z., J.R. Cancado, L.M. Galvao, Z.M. da Luz, S. Filardi Lde, M.E. Pereira, L.M. Santos, and C.B. Cancado. 1993. An experimental and clinical assay with ketoconazole in the treatment of Chagas disease. Mem Inst Oswaldo Cruz. 88:149-153.

Brown BG, Zhao XQ, Chait A, Fisher LD, Cheung MC, Morse JS, Dowdy AA, Marino EK, Bolson EL, Alaupovic P, Frohlich J, Albers JJ. Simvastatin and niacin, antioxidant vitamins, or the combination for the prevention of coronary disease. N Engl J Med. 2001 Nov 29;345(22):1583-92

Campos-Estrada, C., A. Liempi, F. Gonzalez-Herrera, M. Lapier, U. Kemmerling, B. Pesce, J. Ferreira, R. Lopez-Munoz, and J.D. Maya. 2015. Simvastatin and Benznidazole-Mediated Prevention of Trypanosoma cruzi-Induced Endothelial Activation: Role of 15-epi-lipoxin A4 in the Action of Simvastatin. PLoS Negl Trop Dis. 9:e0003770.

Cardillo, F., J.C. Voltarelli, S.G. Reed, and J.S. Silva. 1996. Regulation of Trypanosoma cruzi infection in mice by gamma interferon and interleukin 10: role of NK cells. Infect Immun. 64:128-134.

Cortes-Serra, N., I. Losada-Galvan, M.J. Pinazo, C. Fernandez-Becerra, J. Gascon, and J. Alonso-Padilla. 2020. State-of-the-art in host-derived biomarkers of Chagas disease prognosis and early evaluation of anti-Trypanosoma cruzi treatment response. Biochim Biophys Acta Mol Basis Dis. 1866:165758.

Crespillo-Andujar, C., S. Chamorro-Tojeiro, F. Norman, B. Monge-Maillo, R. Lopez-Velez, and J.A. Perez-Molina. 2018a. Toxicity of nifurtimox as second-line treatment after benznidazole intolerance in patients with chronic Chagas disease: when available options fail. Clin Microbiol Infect. 24:1344 e1341-1344 e1344.

Crespillo-Andujar, C., E. Venanzi-Rullo, R. Lopez-Velez, B. Monge-Maillo, F. Norman, A. Lopez-Polin, and J.A. Perez-Molina. 2018b. Safety Profile of Benznidazole in the Treatment of Chronic Chagas Disease: Experience of a Referral Centre and Systematic Literature Review with Meta-Analysis. Drug Saf. 41:1035-1048.

Duffy, T., C.I. Cura, J.C. Ramirez, T. Abate, N.M. Cayo, R. Parrado, Z.D. Bello, E. Velazquez, A. Munoz-Calderon, N.A. Juiz, J. Basile, L. Garcia, A. Riarte, J.R. Nasser, S.B. Ocampo, Z.E. Yadon, F. Torrico, B.A. de Noya, I. Ribeiro, and A.G. Schijman. 2013. Analytical performance of a multiplex Real-Time PCR assay using TaqMan probes for quantification of Trypanosoma cruzi satellite DNA in blood samples. PLoS Negl Trop Dis. 7:e2000.

Dzikowska-Diduch, O., J. Domienik-Karlowicz, E. Gorska, U. Demkow, P. Pruszczyk, and M. Kostrubiec. 2017. E-selectin and sICAM-1, biomarkers of endothelial function, predict recurrence of venous thromboembolism. Thromb Res. 157:173-180.

Echeverria, L.E., L.Z. Rojas, M.C. Villamizar, C. Luengas, A.M. Chaves, J.A. Rodriguez, R. Campo, C. Clavijo, A.M. Redondo, L.A. Lopez, S.A. Gomez-Ochoa, C.A. Morillo, O.L. Rueda-Ochoa, and O.H. Franco. 2020. Echocardiographic parameters, speckle tracking, and brain natriuretic peptide levels as indicators of progression of indeterminate stage to Chagas cardiomyopathy. Echocardiography. 37:429-438.

Fleiss, J.L., B. Levin, and M.C. Paik. 2003. Statistical Methods for Rates and Proportions. Willey, New York, USA. 800 pp.

Gonzalez-Herrera, F., A. Cramer, P. Pimentel, C. Castillo, A. Liempi, U. Kemmerling, F.S. Machado, and J.D. Maya. 2017. Simvastatin Attenuates Endothelial Activation through 15-Epi-Lipoxin A4 Production in Murine Chronic Chagas Cardiomyopathy. Antimicrob Agents Chemother. 61.

Guzman-Rivera, D., A. Liempi, F. Gonzalez-Herrera, S. Fuentes, I. Carrillo, P. Abarca, C. Castillo, U. Kemmerling, B. Pesce, and J.D. Maya. 2020. Simvastatin improves cardiac function through Notch1 activation in BALB/c mice with chronic Chagas cardiomyopathy. Antimicrob Agents Chemother.

Hasegawa, S., T. Miura, S. Sasaki, H. Madarame, and A. Nakane. 2002. Dysregulation of interleukin-10 and interleukin-12 are involved in the reduced host resistance to Listeria monocytogenes infection in alymphoplastic aly mutant mice. FEMS Immunol Med Microbiol. 32:111-117.

Hernandez, M., S. Wicz, M.H. Santamaria, and R.S. Corral. 2018. Curcumin exerts anti-inflammatory and vasoprotective effects through amelioration of NFAT-dependent endothelin-1 production in mice with acute Chagas cardiomyopathy. Mem Inst Oswaldo Cruz. 113:e180171.

Hiss, F.C., T.F. Lascala, B.C. Maciel, J.A. Marin-Neto, and M.V. Simoes. 2009. Changes in myocardial perfusion correlate with deterioration of left ventricular systolic function in chronic Chagas' cardiomyopathy. JACC Cardiovasc Imaging. 2:164-172.

Jackson, Y., B. Wyssa, and F. Chappuis. 2020. Tolerance to nifurtimox and benznidazole in adult patients with chronic Chagas' disease. J Antimicrob Chemother. 75:690-696.

Jefferies, J.L., and J.A. Towbin. 2010. Dilated cardiomyopathy. Lancet. 375:752-762.

Jenkins DJA, Spence JD, Giovannucci EL, Kim YI, Josse RG, Vieth R, Sahye-Pudaruth S, Paquette M, Patel D, Blanco Mejia S, Viguiliouk E, Nishi SK, Kavanagh M, Tsirakis T, Kendall CWC, Pichika SC, Sievenpiper JL. 2021. Supplemental Vitamins and Minerals for Cardiovascular Disease Prevention and Treatment: JACC Focus Seminar. J Am Coll Cardiol. Feb 2;77(4):423-436

Jercic, M.I., and A. Oyarce. 2019. Recomendaciones técnicas para la selección de método para el tamizaje serológico de la enfermedad de Chagas. D.B.N.y.d.R.I.d.S.P.d.C. Sección Parasitología, editor. Instituto de Salud Publica, Ministerio de Salud, Gobierno de Chile.

Lee, B.Y., K.M. Bacon, M.E. Bottazzi, and P.J. Hotez. 2013. Global economic burden of Chagas disease: a computational simulation model. Lancet Infect Dis. 13:342-348.

Lemos de Oliveira, L.F., J.T. Thackeray, J.A. Marin Neto, M.M. Dias Romano, E.E. Vieira de Carvalho, J. Mejia, D.M. Tanaka, G. Kelly da Silva, D.R. Abdalla, C. Malamut, F.M. Bengel, M. de Lourdes Higuchi, A. Schmidt, E. Cunha-Neto, and M.V. Simoes. 2018. Regional Myocardial Perfusion Disturbance in Experimental Chronic Chagas Cardiomyopathy. J Nucl Med. 59:1430-1436.

Lenk, E.J., W.K. Redekop, M. Luyendijk, C. Fitzpatrick, L. Niessen, W.A. Stolk, F. Tediosi, A.J. Rijnsburger, R. Bakker, J.A.C. Hontelez, J.H. Richardus, J. Jacobson, E.A. Le Rutte, S.J. de Vlas, and J.L. Severens. 2018. Socioeconomic benefit to individuals of achieving 2020 targets for four neglected tropical diseases controlled/eliminated by innovative and intensified disease management: Human African trypanosomiasis, leprosy, visceral leishmaniasis, Chagas disease. PLoS Negl Trop Dis. 12:e0006250.

Llaguno, M., M.V. da Silva, L.R. Batista, D.A.A. da Silva, R.C. de Sousa, L. de Resende, V.J.D. da Silva, E. Lages-Silva, C.J.F. Oliveira, J.R. Machado, D.B.R. Rodrigues, D. Correia, and V. Rodrigues. 2019. T-Cell Immunophenotyping and Cytokine Production Analysis in Patients with Chagas Disease 4 Years after Benznidazole Treatment. Infect Immun. 87.

Lopez-Munoz, R., M. Faundez, S. Klein, S. Escanilla, G. Torres, D. Lee-Liu, J. Ferreira, U. Kemmerling, M. Orellana, A. Morello, A. Ferreira, and J.D. Maya. 2010. Trypanosoma cruzi: In vitro effect of aspirin with nifurtimox and benznidazole. Exp Parasitol. 124:167-171.

Marin-Neto, J.A., M.V. Simoes, and A. Rassi Junior. 2013. Pathogenesis of chronic Chagas cardiomyopathy: the role of coronary microvascular derangements. Rev Soc Bras Med Trop. 46:536-541.

Marti-Carvajal, A.J., and J.S. Kwong. 2016. Pharmacological interventions for treating heart failure in patients with Chagas cardiomyopathy. Cochrane Database Syst Rev. 7:CD009077.

MINSAL. 2017. Manual de procedimiento para la atención de pacientes con enfermedad de Chagas. D.d.P.y.C.E.M.d.S. Departamento de Enfermedades Transmisibles, editor. Ministerio de Salud Publica, Gobierno de Chile.

Molina-Berrios, A., C. Campos-Estrada, N. Henriquez, M. Faundez, G. Torres, C. Castillo, S. Escanilla, U. Kemmerling, A. Morello, R.A. Lopez-Munoz, and J.D. Maya. 2013a. Protective role of acetylsalicylic acid in experimental Trypanosoma cruzi infection: evidence of a 15-epi-lipoxin A(4)-mediated effect. PLoS Negl Trop Dis. 7:e2173.

Molina-Berrios, A., C. Campos-Estrada, M. Lapier, J. Duaso, U. Kemmerling, N. Galanti, J. Ferreira, A. Morello, R. Lopez-Munoz, and J.D. Maya. 2013b. Protection of vascular endothelium by aspirin in a murine model of chronic Chagas' disease. Parasitol Res. 112:2731-2739.

Molina-Berrios, A., C. Campos-Estrada, M. Lapier, J. Duaso, U. Kemmerling, N. Galanti, M. Leiva, J. Ferreira, R. Lopez-Munoz, and J.D. Maya. 2013c. Benznidazole prevents endothelial damage in an experimental model of Chagas disease. Acta Trop. 127:6-13.

Molina, I., J. Gomez i Prat, F. Salvador, B. Trevino, E. Sulleiro, N. Serre, D. Pou, S. Roure, J. Cabezos, L. Valerio, A. Blanco-Grau, A. Sanchez-Montalva, X. Vidal, and A. Pahissa. 2014. Randomized trial of posaconazole and benznidazole for chronic Chagas' disease. N Engl J Med. 370:1899-1908.

Molina, I., F. Salvador, A. Sanchez-Montalva, M.A. Artaza, R. Moreno, L. Perin, A. Esquisabel, L. Pinto, and J.L. Pedraz. 2017. Pharmacokinetics of Benznidazole in Healthy Volunteers and Implications in Future Clinical Trials. Antimicrob Agents Chemother. 61.

Morillo, C.A., J.A. Marin-Neto, A. Avezum, S. Sosa-Estani, A. Rassi, Jr., F. Rosas, E. Villena, R. Quiroz, R. Bonilla, C. Britto, F. Guhl, E. Velazquez, L. Bonilla, B. Meeks, P. Rao-Melacini, J. Pogue, A. Mattos, J. Lazdins, A. Rassi, S.J. Connolly, S. Yusuf, and B. Investigators. 2015. Randomized Trial of Benznidazole for Chronic Chagas' Cardiomyopathy. N Engl J Med. 373:1295-1306.

Moroni, S., M.E. Marson, G. Moscatelli, G. Mastrantonio, M. Bisio, N. Gonzalez, G. Ballering, J. Altcheh, and F. Garcia-Bournissen. 2019. Negligible exposure to nifurtimox through breast milk during maternal treatment for Chagas Disease. PLoS Negl Trop Dis. 13:e0007647.

Nunes, M.C.P., A. Beaton, H. Acquatella, C. Bern, A.F. Bolger, L.E. Echeverria, W.O. Dutra, J. Gascon, C.A. Morillo, J. Oliveira-Filho, A.L.P. Ribeiro, J.A. Marin-Neto, E. American Heart Association Rheumatic Fever, Y. Kawasaki Disease Committee of the Council on Cardiovascular Disease in the, C. Council on, N. Stroke, and C. Stroke. 2018. Chagas Cardiomyopathy: An Update of Current Clinical Knowledge and Management: A Scientific Statement From the American Heart Association. Circulation. 138:e169-e209.

OPS. 2018. Guía para el diagnóstico y el tratamiento de la enfermedad de Chagas. Organización Panamericana de la Salud, Washington D.C. U.S.A.

Parrado, R., J.C. Ramirez, A. de la Barra, C. Alonso-Vega, N. Juiz, L. Ortiz, D. Illanes, F. Torrico, J. Gascon, F. Alves, L. Flevaud, L. Garcia, A.G. Schijman, and I. Ribeiro. 2019. Usefulness of Serial Blood Sampling and PCR Replicates for Treatment Monitoring of Patients with Chronic Chagas Disease. Antimicrob Agents Chemother. 63.

Pengue, C., G. Cesar, M.G. Alvarez, G. Bertocchi, B. Lococo, R. Viotti, M.A. Natale, M.D. Castro Eiro, S.S. Cambiazzo, N. Perroni, M. Nunez, M.C. Albareda, and S.A. Laucella. 2019. Impaired frequencies and function of platelets and tissue remodeling in chronic Chagas disease. PLoS One. 14:e0218260.

Perez-Anton, E., A. Egui, M.C. Thomas, M. Simon, M. Segovia, and M.C. Lopez. 2020. Immunological exhaustion and functional profile of CD8(+) T lymphocytes as cellular biomarkers of therapeutic efficacy in chronic Chagas disease patients. Acta Trop. 202:105242.

Rassi, A., J.M. de Rezende, A.O. Luquetti, and A. Rassi. 2017a. 28 - Clinical phases and forms of Chagas disease. In American Trypanosomiasis Chagas Disease (Second Edition). J. Telleria and M. Tibayrenc, editors. Elsevier, London. 653-686.

Rassi, A., Jr., J.A.N. Marin, and A. Rassi. 2017b. Chronic Chagas cardiomyopathy: a review of the main pathogenic mechanisms and the efficacy of aetiological treatment following the BENznidazole Evaluation for Interrupting Trypanosomiasis (BENEFIT) trial. Mem Inst Oswaldo Cruz. 112:224-235.

Rassi, A., Jr., A. Rassi, W.C. Little, S.S. Xavier, S.G. Rassi, A.G. Rassi, G.G. Rassi, A. Hasslocher-Moreno, A.S. Sousa, and M.I. Scanavacca. 2006. Development and validation of a risk score for predicting death in Chagas' heart disease. N Engl J Med. 355:799-808.

Rassi, A., Jr., A. Rassi, and J.A. Marin-Neto. 2010. Chagas disease. Lancet. 375:1388-1402.

Rassi, A.J.R., A; Marin-Neto 2010. Chagas disease. Lancet. 375:1388-1402.

Requena-Mendez, A., S. Bussion, E. Aldasoro, Y. Jackson, A. Angheben, D. Moore, M.J. Pinazo, J. Gascon, J. Munoz, and E. Sicuri. 2017. Cost-effectiveness of Chagas disease screening in Latin

American migrants at primary health-care centres in Europe: a Markov model analysis. Lancet Glob Health. 5:e439-e447.

Ribeiro, V., N. Dias, T. Paiva, L. Hagstrom-Bex, N. Nitz, R. Pratesi, and M. Hecht. 2020. Current trends in the pharmacological management of Chagas disease. Int J Parasitol Drugs Drug Resist. 12:7-17.

Rojas, L.Z., M. Glisic, L. Pletsch-Borba, L.E. Echeverria, W.M. Bramer, A. Bano, N. Stringa, A. Zaciragic, B. Kraja, E. Asllanaj, R. Chowdhury, C.A. Morillo, O.L. Rueda-Ochoa, O.H. Franco, and T. Muka. 2018. Electrocardiographic abnormalities in Chagas disease in the general population: A systematic review and meta-analysis. PLoS Negl Trop Dis. 12:e0006567.

Rossi, M.A., H.B. Tanowitz, L.M. Malvestio, M.R. Celes, E.C. Campos, V. Blefari, and C.M. Prado. 2010. Coronary microvascular disease in chronic Chagas cardiomyopathy including an overview on history, pathology, and other proposed pathogenic mechanisms. PLoS Negl Trop Dis. 4.

Sales Junior, P.A., I. Molina, S.M. Fonseca Murta, A. Sanchez-Montalva, F. Salvador, R. Correa-Oliveira, and C.M. Carneiro. 2017. Experimental and Clinical Treatment of Chagas Disease: A Review. Am J Trop Med Hyg. 97:1289-1303.

Schijman, A.G., J. Altcheh, J.M. Burgos, M. Biancardi, M. Bisio, M.J. Levin, and H. Freilij. 2003. Aetiological treatment of congenital Chagas' disease diagnosed and monitored by the polymerase chain reaction. J Antimicrob Chemother. 52:441-449.

Schmunis, G. 2013. Status of and cost of Chagas disease worldwide. Lancet Infect Dis. 13:283-284.

Serhan, C.N. 2017. Discovery of specialized pro-resolving mediators marks the dawn of resolution physiology and pharmacology. Mol Aspects Med. 58:1-11.

Serhan, C.N., and N. Chiang. 2013. Resolution phase lipid mediators of inflammation: agonists of resolution. Curr Opin Pharmacol. 13:632-640.

Sguassero, Y., K.N. Roberts, G.B. Harvey, D. Comande, A. Ciapponi, C.B. Cuesta, C. Aguiar, A.M. Castro, E. Danesi, A.L. de Andrade, M. de Lana, J.M. Escriba, D.L. Fabbro, C.D. Fernandes, M. Flores-Chavez, A.M. Hasslocher-Moreno, Y. Jackson, C.D. Lacunza, G.F. Machado-de-Assis, M. Maldonado, W.S.F. Meira, I. Molina, M.M. Monje-Rumi, C. Munoz-San Martin, L. Murcia, C. Nery de Castro, O. Sanchez Negrette, M. Segovia, C.A.N. Silveira, A. Solari, M. Steindel, M.L. Streiger, N. Vera de Bilbao, I. Zulantay, and S. Sosa-Estani. 2018. Course of serological tests in treated subjects with chronic Trypanosoma cruzi infection: A systematic review and meta-analysis of individual participant data. Int J Infect Dis. 73:93-101.

Sousa, G.R., J.A. Gomes, M.P. Damasio, M.C. Nunes, H.S. Costa, N.I. Medeiros, R.C. Fares, A.T. Chaves, R. Correa-Oliveira, and M.O. Rocha. 2017. The role of interleukin 17-mediated immune response in Chagas disease: High level is correlated with better left ventricular function. PLoS One. 12:e0172833.

Souza-Silva, T.G., L.F. Diniz, A. Lia Mazzeti, A.A.S. Mendonca, R.V. Goncalves, and R.D. Novaes. 2019. Could angiotensin-modulating drugs be relevant for the treatment of Trypanosoma cruzi infection? A systematic review of preclinical and clinical evidence. Parasitology. 146:914-927.

Spite, M., and C.N. Serhan. 2010. Novel lipid mediators promote resolution of acute inflammation: impact of aspirin and statins. Circ Res. 107:1170-1184.

Stein JH, Carlsson CM, Papcke-Benson K, Aeschlimann SE, Bodemer A, Carnes M, McBride PE. The effects of lipid-lowering and antioxidant vitamin therapies on flow-mediated vasodilation of the brachial artery in older adults with hypercholesterolemia. J Am Coll Cardiol. 2001 Dec;38(7):1806-13.

Stein, C., C.B. Migliavaca, V. Colpani, P.R. da Rosa, D. Sganzerla, N.E. Giordani, S. Miguel, L.N. Cruz, C.A. Polanczyk, A.L.P. Ribeiro, and M. Falavigna. 2018. Amiodarone for arrhythmia in patients with Chagas disease: A systematic review and individual patient data meta-analysis. PLoS Negl Trop Dis. 12:e0006742.

Stone PH, Lloyd-Jones DM, Kinlay S, Frei B, Carlson W, Rubenstein J, Andrews TC, Johnstone M, Sopko G, Cole H, Orav J, Selwyn AP, Creager MA; Vascular Basis Study Group. Effect of intensive lipid lowering, with or without antioxidant vitamins, compared with moderate lipid lowering on myocardial ischemia in patients with stable coronary artery disease: the Vascular Basis for the Treatment of Myocardial Ischemia Study. Circulation. 2005 Apr 12;111(14):1747-55

Tanaka, D.M., L.F.L. de Oliveira, J.A. Marin-Neto, M.M.D. Romano, E.E.V. de Carvalho, A.C.L. de Barros Filho, F.F.F. Ribeiro, J.M. Cabeza, C.D. Lopes, C.G. Fabricio, N. Kesper, H.T. Moreira, L. Wichert-Ana, A. Schmidt, M.L. Higuchi, E. Cunha-Neto, and M.V. Simoes. 2019. Prolonged dipyridamole administration reduces myocardial perfusion defects in experimental chronic Chagas cardiomyopathy. J Nucl Cardiol. 26:1569-1579.

Torrico, F., J. Gascon, L. Ortiz, C. Alonso-Vega, M.J. Pinazo, A. Schijman, I.C. Almeida, F. Alves, N. Strub-Wourgaft, I. Ribeiro, and E.S. Group. 2018. Treatment of adult chronic indeterminate Chagas disease with benznidazole and three E1224 dosing regimens: a proof-of-concept, randomised, placebo-controlled trial. Lancet Infect Dis. 18:419-430.

Varela, M.T., and J.P.S. Fernandes. 2020. Natural Products: Key Prototypes to Drug Discovery Against Neglected Diseases Caused by Trypanosomatids. Curr Med Chem. 27:2133-2146.

WHO. 2011. Causes of Death in 2008. In Mortality and global health estimates. Vol. 2013. World Health Organization, Global Health Observatory Data Repository.

WHO. 2015. Investing to overcome the global impact of neglected tropical diseases: third WHO report on neglected tropical diseases. World Health Organization, Geneva. 191 p. pp.

WHO. 2018. Global Health Estimates 2016: Disease burden by Cause, Age, Sex, by Country and by Region, 2000-2016, Geneva.

Zabihi M, Askarian F, Hekmatimoghaddam S, Rashidi Nooshabadi M, Zabihi MS, Mousavinasab SR. Ascorbic Acid Significantly Decreases Creatine Kinase Plasma Levels in an Animal Model of Statin/Fibrate-Induced Myopathy. Adv Pharmacol Pharm Sci. 2021 Dec 29;2021:5539595
