# Supplementary material for: Effect of statins on inflammation and cardiac function in patients with chronic Chagas disease: A protocol for pathophysiological studies in a multicenter, placebo-controlled, proof-of-concept phase II trial
Source: PLoS One. 2023 Jan 13;18(1):e0280335. doi: 10.1371/journal.pone.0280335 (PMC9838836; doi:10.1371/journal.pone.0280335)
Supplement: S4 File — (PDF) [file pone.0280335.s004.pdf]

Santiago, May 17th 2022

Dr. Juan Diego Maya
Instituto de Ciencias Biomédicas
Facultad de Medicina
Universidad de Chile
Presente

Ref.: Proyecto 009-2021

Dear Dr. Maya,

The Committee on Ethics in Human Research (CEISH), dated May 17, 2022, informs you of the letter received dated 05/17/2022 regarding your project: "**EFFECT OF STATINS ON INFLAMMATION AND CARDIAC FUNCTION IN PATIENTS WITH CHRONIC CHAGAS DISEASE: PHYSIOPATHOLOGICAL STUDIES IN A CONCEPT TESTING MULTICENTRIC CLINICAL TRIAL**".

Ref.: Amendment.

- This Committee takes cognizance of the amendment to the Informed Consent and the protocol, in which specifications were included concerning assessing the primary endpoint.

- The following documents are approved:

  1. Protocol version 3.2 of May 11, 2022.
  2. Informed Consent Form (version of May 17, 2022)

Without further ado, Yours faithfully,

JAVIERA DE LOS ANDES COBO RIVEROS

Nta. Javiera Cobo Riveros
Executive Secretary CEISH

Proyecto Nº 009-2021
Archivo.002

Teléfono 22-978.95.36 - Email: ceish.med@uchile.cl
