# Supplementary material for: Effect of statins on inflammation and cardiac function in patients with chronic Chagas disease: A protocol for pathophysiological studies in a multicenter, placebo-controlled, proof-of-concept phase II trial
Source: PLoS One. 2023 Jan 13;18(1):e0280335. doi: 10.1371/journal.pone.0280335 (PMC9838836; doi:10.1371/journal.pone.0280335)
Supplement: Attachment — Submitted filename: Answer to reviewers.docx [file pone.0280335.s005.docx]

Answer to the reviewers

On behalf of all the authors of the present protocol, we would like to thank the reviewers for their thorough work, which undoubtedly improved the new version of the manuscript.

Below you will find the point-by-point responses to each indication or suggestion made by the reviewers.

6. Review Comments to the Author

Please use the space provided to explain your answers to the questions above and, if applicable, provide comments about issues authors must address before this protocol can be accepted for publication. You may also include additional comments for the author, including concerns about research or publication ethics.

You may also provide optional suggestions and comments to authors that they might find helpful in planning their study.


(Please upload your review as an attachment if it exceeds 20,000 characters)

Reviewer #1: This is an interesting protocol to assess effect of statins on inflammation and cardiac function in patients with chronic Chagas disease from a multicenter, placebo-controlled, proof-of-concept phase II trial. Well written and followed by the SPIRIT 2013 statement. I have a few comments to the authors.

1. The primary outcome of this study is the change in cardiac function, but sample size calculation was done by change of inflammation biomarkers. Why not the authors conduct sample size calculation by the primary outcome? Could the authors comment on it?

We thank the reviewer for drawing attention to this point. A transcription issue was maintained from the other document versions that remained. Nonetheless, the sample size calculation was made considering the improvement in cardiac function. For this purpose, the composite analysis of the plasma levels of BNP, and cTnT, the resting 12-lead ECG (cardiac rate, QT segment duration, changes in electrical conduction as determined by QRS segment duration and morphology), and ejection fraction determined by a 2D-echocardiogram is proposed as an indicator. Thus, line 356-357 vas corrected, as can be seen in the marked version.

2. Please check figure and table numbers throughout manuscript. E.g. page 12. line 247, "Inclusion and exclusion criteria for subject recruitment are defined in figure 2". Page 14, line 293-4 "Other withdrawal criteria are indicated in Fig. 2." These should be Figure 3.

Thank you for this indication. The proper call for figure 3 was corrected.

Reviewer #2: I have reviewed the study protocol paper entitled “Effect of statins on inflammation and cardiac function in patients with chronic Chagas disease: A protocol for pathophysiological studies in a multicenter, placebo-controlled, proof-of-concept phase II trial” with great interest.

This study will provide an important contribution to the treatment of Chagas disease.
The protocol has been developed well.
I feel this paper would be appropriate for the journal.

Again, thank you very much for your kind comments.
